# Rapid discovery of monoclonal antibodies by microfluidics-enabled FACS of single pathogen-specific antibody-secreting cells

**DOI:** 10.1038/s41587-024-02346-5

**Published:** 2024-08-14

**Authors:** Katrin Fischer, Aleksei Lulla, Tsz Y. So, Pehuén Pereyra-Gerber, Matthew I. J. Raybould, Timo N. Kohler, Juan Carlos Yam-Puc, Tomasz S. Kaminski, Robert Hughes, Gwendolyn L. Pyeatt, Florian Leiss-Maier, Paul Brear, Nicholas J. Matheson, Charlotte M. Deane, Marko Hyvönen, James E. D. Thaventhiran, Florian Hollfelder

**Affiliations:** 1 Department of Biochemistry, University of Cambridge, Cambridge, UK; 2 MRC Toxicology Unit, Gleeson Building, Cambridge, UK; 3 Cambridge Institute for Therapeutic Immunology and Infectious Disease (CITIID), Department of Medicine, University of Cambridge, Cambridge, UK; 4 Oxford Protein Informatics Group, Department of Statistics, University of Oxford, Oxford, UK; 5 Department of Molecular Biology, Institute of Biochemistry, Faculty of Biology, University of Warsaw, Warsaw, Poland; 6 NHS Blood and Transplant, Cambridge, UK

**Keywords:** Biotechnology, High-throughput screening

## Abstract

Monoclonal antibodies are increasingly used to prevent and treat viral infections and are pivotal in pandemic response efforts. Antibody-secreting cells (ASCs; plasma cells and plasmablasts) are an excellent source of high-affinity antibodies with therapeutic potential. Current methods to study antigen-specific ASCs either have low throughput, require expensive and labor-intensive screening or are technically demanding and therefore not widely accessible. Here we present a straightforward technology for the rapid discovery of monoclonal antibodies from ASCs. Our approach combines microfluidic encapsulation of single cells into an antibody capture hydrogel with antigen bait sorting by conventional flow cytometry. With our technology, we screened millions of mouse and human ASCs and obtained monoclonal antibodies against severe acute respiratory syndrome coronavirus 2 with high affinity (<1 pM) and neutralizing capacity (<100 ng ml^−1^) in 2 weeks with a high hit rate (>85% of characterized antibodies bound the target). By facilitating access to the underexplored ASC compartment, the approach enables efficient antibody discovery and immunological studies into the generation of protective antibodies.

## Abstract (web summary)

Antigen-specific antibodies secreted by plasma cells are rapidly identified using microfluidics and fluorescence-activated cell sorting (FACS).

## Main

Antibodies produced by our immune system in response to infection or vaccination are invaluable tools in therapy, diagnostics and research. Human immune repertoires are an excellent source for the generation of potent antibody therapeutics and have been successfully used in the fight against viral infections, such as COVID-19 (refs. ^1,2^). Human-derived antibodies have advantages over animal-derived or in vitro-selected binders—they are human-compatible without optimization and are thought to have less off-target binding, a more favorable safety profile and better overall developability^3^. Until now, human-derived therapeutic monoclonal antibodies (mAbs) against various infectious diseases have been primarily sourced from memory B cells^1,4–6^. These cells can be readily selected in a high-throughput manner with fluorescently labeled antigen baits by fluorescence-activated cell sorting (FACS) because they retain the membrane-bound version of their immunoglobulins (Igs) on the cell surface^6^. Single-sorted cells are then sequenced to obtain the antibody genes for recombinant expression.

In contrast, antibody-secreting cells (ASCs; plasma cells and plasmablasts) have been undervalued as a rich source of highly specific antibodies because of technological limitations and their limited accessibility in peripheral blood^3^. Antibodies isolated from ASCs are thought to have on average higher affinity than those derived from memory B cells^7–9^. Additionally, ASCs are responsible for the active humoral immune response, that is, they secrete the circulating antibodies that confer protection against invading pathogens^10^. Interrogating this compartment therefore brings us closer to understanding the secreted antibody repertoire and can help bridge the gap between proteomic profiling of plasma antibodies and bulk sequencing of the B cell repertoire^11^.

However, studying the specificity of single ASCs is difficult because they secrete their antibodies and express few or no Igs on the surface^12^. For this reason, ASCs cannot be interrogated by conventional FACS in an antigen-specific manner with high throughput. Traditionally, the ASC compartment has therefore been interrogated by either unbiased plasmablast sorting^13,14^, which does not give information about the antigen specificity of single cells, or enzyme-linked immunospot (ELISpot) assays, which enumerate the frequency of antigen-specific cells but are not able to recover their genotype^15^.

Because the percentage of suitable antigen binders within the plasmablast population can be low, unbiased sorting can require substantial time and resource investment to screen antibodies from all cells for validation of binding. Recently, approaches based on compartmentalization of single cells in water-in-oil emulsion droplets have been applied in antibody discovery^16–20^ and sequencing^21–23^. Alternatively, antibody discovery from ASCs can be performed with commercial optofluidic systems^24^. However, functional assays in droplets are technically demanding, and standalone machines are prohibitively expensive, therefore both approaches are not accessible to the wider research community.

We address the limitations of current ASC screening methods by enabling the high-throughput interrogation of antigen-specific ASCs by conventional FACS. In our workflow, we first use droplet microfluidics to encapsulate single cells into an antibody capture hydrogel at 10^7^ cells per h, creating a stable capture matrix around the cell that enables the concentration of secreted antibodies and simple addition and removal of detection reagents. We then use the multiplexed detection and high-throughput sorting capabilities of FACS to isolate antigen-specific ASCs for single-cell sequencing and recombinant antibody expression. The modular nature of the method enables its extension to other secreted molecules by simple replacement of capture and detection reagents.

We demonstrate the utility of this approach by screening millions of primary immune cells to isolate mAbs against severe acute respiratory syndrome coronavirus 2 (SARS-CoV-2) from mouse and human ASCs. Tapping into these repertoires allowed us to collect a diverse pool of sequences of secreted antibodies. Of a representative subset of human antibodies, 95% bound the respective antigen, many with subnanomolar affinities and high neutralizing capacities (<100 ng ml^−1^), highlighting the benefits of interrogating the locus of the active humoral response. Our technology can generate pathogen-specific antibodies within 2 weeks, both democratizing and fast-tracking the development of antibody drug candidates.

## Results

### Antibody capture system and screening workflow

Identification of single antigen-specific ASCs presents a considerable challenge—target cells secreting antibodies with desirable properties have to be selected from a vast pool of cells producing nonspecific binders while maintaining the link between the phenotype of the secreted antibody and the cell that encodes its sequence (genotype).

**Fig. 1:**
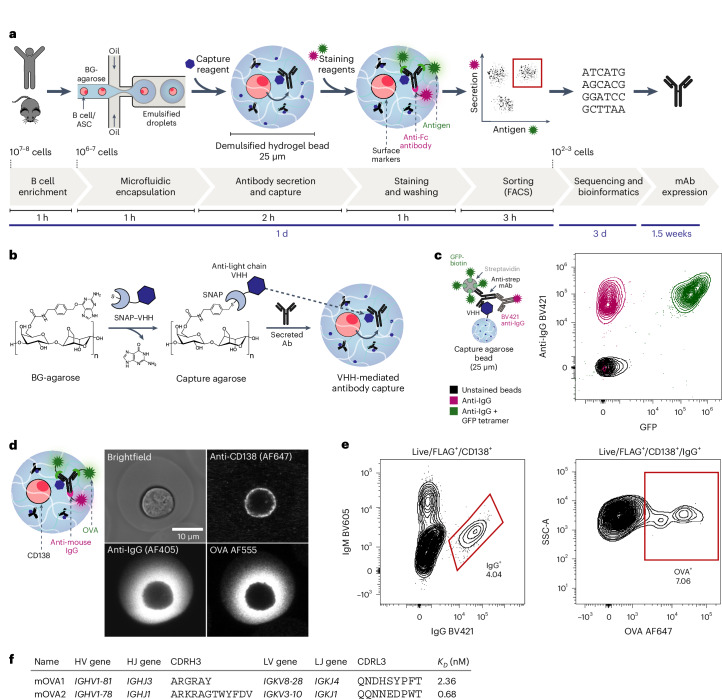
Workflow for high-throughput functional analysis of antibodies secreted by single cells. **a**, Overview of the workflow—B cells (or enriched ASCs) are isolated from mice (bone marrow or spleen) or human PBMCs. Cells are mixed with liquid BG-agarose at 37 °C and encapsulated into picolitre water-in-oil emulsion droplets using a flow-focusing junction. Droplets are collected on ice for agarose gelation and demulsified, creating stable hydrogel beads around each cell. The BG-agarose is converted into an antibody capture matrix by the addition of recombinant capture reagents that are fused to the SNAP-tag, an enzyme that reacts with BG moieties. During incubation, antibodies secreted by a single cell are captured in the hydrogel surrounding the cell. Cells that have secreted antigen-specific antibodies are identified with fluorescently labeled detection reagents (antigens, secondary antibodies and antibodies against cell-surface markers), sorted using flow cytometry and sequenced. Antibody sequences can be obtained within 4 days, and recombinant antibodies for testing are generated in 2 weeks. **b**, Agarose-based antibody capture matrix. Agarose is chemically modified to contain BG moieties that react covalently with the SNAP-tag. Single-domain antibodies (VHHs) against the constant region of antibody light chains are expressed as SNAP-tag fusions and immobilized in the BG-agarose hydrogel, creating the capture matrix. **c**, Antibody capture by BG-agarose hydrogel beads functionalized with VHH–SNAP. Antibody capture (anti-streptavidin mouse IgG) and antigen binding (streptavidin–GFP) were analyzed by flow cytometry. The plot shows at least 220 events per condition at a 5% contour level. **d**, Antibody secretion by single OVA-specific mouse bone marrow plasma cells. Representative confocal microscopy image from two independent experiments showing a single cell encapsulated in VHH-functionalized BG-agarose stained with fluorescently labeled OVA (AF555), anti-CD138 antibodies (AF647) and anti-mouse IgG antibodies (AF405). **e**, Sorting of OVA-specific mouse bone marrow plasma cells. Hydrogel beads containing plasma cells that secreted OVA-specific IgG were sorted by FACS (gated as live/FLAG^+^/CD138^+^/IgM^−^/IgG^+^). The plots show 10,168 (CD138^+^) and 411 (IgG^+^) events at 2% contour level. **f**, Characteristics of mouse anti-OVA antibodies—variable domain genes (V and J), third complementarity-determining region amino acid sequences (CDR3s) and equilibrium dissociation constants (*K*_*D*_).

We have addressed this challenge by compartmentalizing single ASCs into an antibody capture hydrogel by automated droplet microfluidics (at a rate of up to 10^7^ cells per h), followed by the selection of the secreted antibody specificity with fluorescently labeled antigens by FACS at a similar speed (Fig. 1a). This combination of microfluidics and FACS enables the high throughput that is crucial for the success of antibody discovery campaigns.

**Extended Data Fig. 1:**
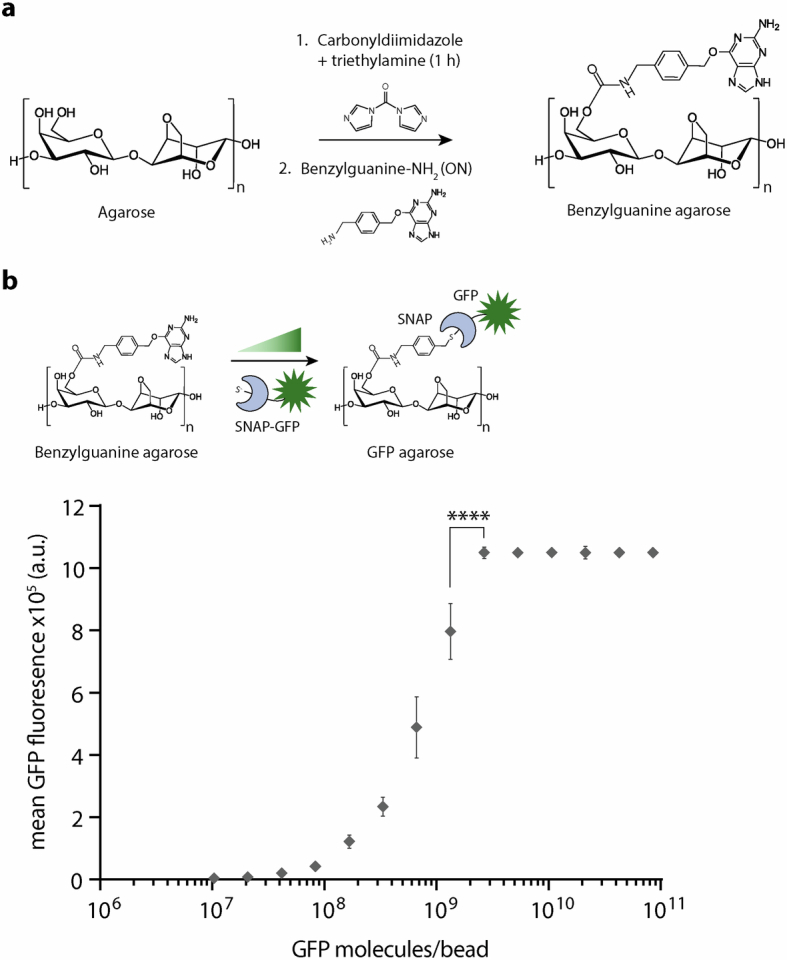
Synthesis and characterization of benzylguanine (BG)-agarose. (**a**) Synthesis of BG-agarose. (**b**) Assessment of 1.5% (wt/vol) BG-agarose capture valency. BG-agarose beads were incubated with different amounts of GFP-SNAP, and the GFP fluorescence of the beads was analyzed by flow cytometry. The fluorescence signal saturates between 1.3 and 2.7 × 10^9^ GFP molecules/bead. Data are represented as mean values ± SD, and at least 18,827 beads were analyzed per condition (unpaired *t*-test, two-sided p < 0.0001). Data from one independent experiment is shown. Source data

**Extended Data Fig. 2:**
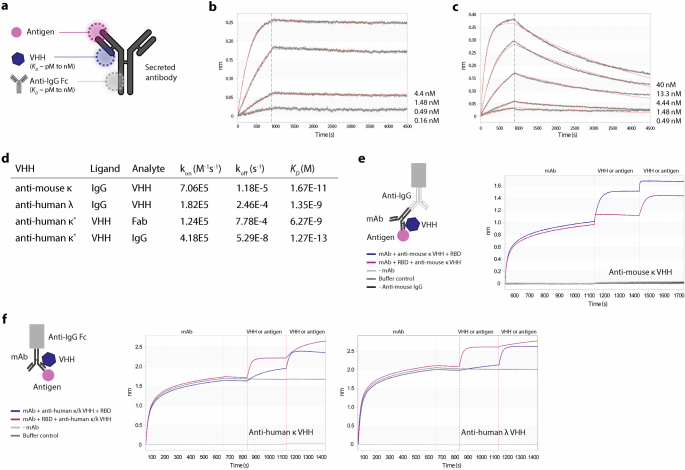
Requirements for capture/detection of secreted proteins and characterization of anti-light chain VHHs for antibody capture. (**a**) Capture matrix requirements for secreted proteins: epitopes of capture reagent (for example, VHH), detection reagent (for example, fluorescently labeled anti-IgG antibody) and antigen should not overlap. VHHs bind to the light-chain constant region, enabling simultaneous binding to the antigen binding site of V_H_/V_L_. To avoid interfering with antibody capture, the detection antibody should bind to the constant region of the heavy chain, preferably the Fc part (C_H_2/C_H_3). Binding affinities should be in the low nM to pM range. (**b**) Determination of binding affinity of anti-mouse κ VHH against recombinant mouse IgG1κ antibody by biolayer interferometry with antibody as ligand and VHH as analyte: K_D_ = 0.0167 ± 0.001 nM, χ^2^ = 0.1417, R^2^ = 0.9991. Tested VHH concentrations: 0.16–4.4 nM. (**c**) Determination of binding affinity of anti-human λ VHH against recombinant human IgG1λ antibody by biolayer interferometry with antibody as ligand and VHH as analyte: K_D_ = 1.35 ± 0.004 nM, χ^2^ = 0.7345, R^2^ = 0.9967. Tested VHH concentrations: 0.49–40 nM. (**d**) Association rates (k_on_), dissociation rates (k_off_) and affinities (K_D_) of VHHs showcasing the benefit of multivalent binding. Values for the anti-human κ VHH have been determined previously by biolayer interferometry for the monovalent (VHH as ligand and Fab as analyte) and bivalent (VHH as ligand and IgG as analyte) interaction and are denoted by an asterisk (*)^82^. (**e**) Biolayer interferometry assay to determine epitope overlap of capture and detection reagents. Binding of anti-mouse κ VHH, anti-mouse IgG1 and RBD antigen to a recombinant mouse anti-RBD antibody. Data of VHH binding followed by RBD binding is shown in blue, while the reverse order of binding is shown in purple. (**f**) Biolayer interferometry assay to determine epitope overlap of capture and detection reagents. Binding of anti-human κ or λ VHHs, anti-human IgG Fc and RBD antigen to recombinant human anti-RBD antibodies. Data of VHH binding followed by RBD binding are shown in blue, while the reverse order of binding is shown in purple.

Specifically, B cells are mixed with liquid benzylguanine (BG)-agarose (at 37 °C) and encapsulated into monodisperse water-in-oil emulsion droplets of 25 µm diameter at kilohertz rates. The droplets are collected on ice (to solidify the agarose) and demulsified, creating a stable agarose microcompartment around each cell. BG-modification creates covalent attachment sites for the SNAP-tag, a 20 kDa engineered human O6-alkylguanine-DNA alkyltransferase that reacts specifically, quickly and irreversibly with different BG-containing substrates^25^. We synthesized BG-agarose by chemical modification of commercial low-melting-point agarose in a simple, two-step procedure (Extended Data Fig. 1a). To create an antibody capture matrix, we functionalized the BG sites with recombinant SNAP-tag fusion proteins of single-domain antibodies (VHHs) that bind to the constant region of antibody light chains (Fig. 1b; see Extended Data Fig. 2a for detailed considerations regarding the design of the capture matrix)^26,27^. This capture approach is highly modular, as any protein fused to the SNAP-tag can be immobilized in the agarose matrix. Through covalent functionalization with antibody capture reagents, antibodies secreted by encapsulated cells are immobilized around the cell, physically linking the secreted antibody and the cell of origin. With two different VHHs, recognizing either κ or λ light chains, any secreted antibody (for example, IgG, IgM and IgA) from any ASC can be captured efficiently. Light-chain-mediated capture enables multivalent binding in the hydrogel (one IgG molecule has two constant light chains). This capture mechanism leads to substantial avidity effects that significantly decrease the apparent rate constant for the dissociation (*k*_off_; Extended Data Fig. 2b–d) and increase the half-life of the VHH–antibody complex. Consequently, we expect minimal dissociation of captured antibodies from the VHHs during the course of the experiment. We have chosen VHHs as antibody capture reagents due to their small size, high affinity, stability and straightforward expression as SNAP-tag-fusions in *Escherichia coli*. Additionally, the chosen VHHs do not exhibit binding to antibodies from other species, which ensures their compatibility with antibody-based flow cytometry stainings^26,27^.

In the hydrogel, antibodies and cells are accessible for staining with fluorescently labeled detection reagents and antigens due to the large pore size of the agarose (>100 nm^28,29^), while unbound reagents can be removed by washing. Additionally, the small size (25 µm diameter) and monodispersity of the hydrogel beads enable sorting by conventional FACS devices. Hydrogel beads can therefore essentially be stained, washed and sorted like single cells, but they also provide additional information about the secreted antibody (secreted amount, isotype and antigen specificity). Finally, single-sorted cells in hydrogel beads are sequenced to enable recombinant expression and characterization of antigen-specific antibodies. FACS allows the detection of several phenotypic parameters or different antigens in one experiment and the correlation of the sequence of single ASCs with phenotypic antibody information through index sorting.

We estimated the capture capacity of the BG-agarose by reacting BG-agarose beads (25 µm diameter, 1.5% (wt/vol) agarose) with different quantities of a GFP-SNAP-tag fusion protein (GFP-SNAP), followed by the analysis of the GFP fluorescence of the beads by flow cytometry (Extended Data Fig. 1b). We observed saturation of the GFP signal at over 10^9^ immobilized GFP molecules per bead. In the literature, average secretion rates of single ASCs range from 10^3^ to 10^5^ antibodies per s^30,31^, indicating that a completely functionalized capture matrix would not become saturated even after several hours of antibody secretion. Notably, in our system, the number of antibody capture sites is a function of bead size and the amount of added VHH–SNAP and is therefore well-defined and controllable. In contrast to capture on the cell surface^32,33^, antibody capture capacity does not depend on cell-intrinsic properties such as cell-surface molecules and should therefore be relatively uniform across the ASC population.

Next, we showed that light-chain-mediated capture enables both the interrogation of antigen binding and the detection of immobilized antibodies by flow cytometry (Fig. 1c). We captured a commercial anti-streptavidin antibody (IgG) in VHH-functionalized BG-agarose beads and stained the beads with both streptavidin–biotin–GFP and anti-IgG antibodies. Analysis by flow cytometry confirmed that captured antibodies can simultaneously bind to the functionalized agarose, the antigen, and the detection antibodies. Consistent with these findings, the epitopes of VHHs, anti-IgG antibodies and antigens do not overlap (Extended Data Fig. 2e,f).

### Antibody capture from single mouse bone marrow plasma cells

**Extended Data Fig. 3:**
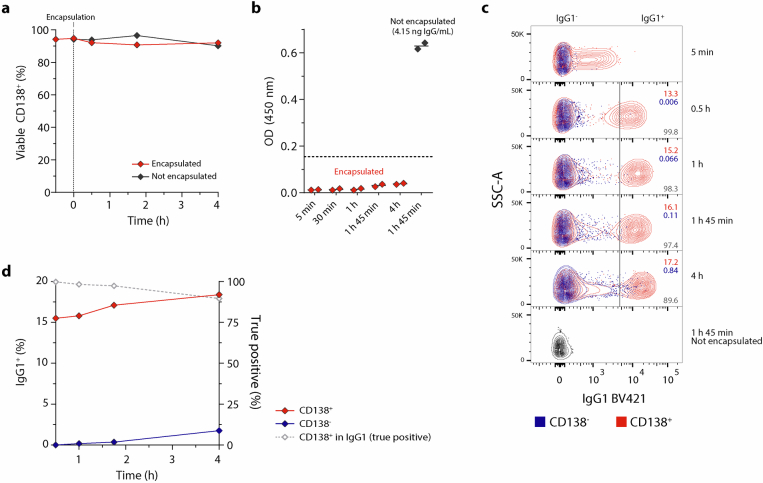
Cell survival and IgG secretion of encapsulated mouse bone marrow plasma cells over time. (**a**) Cell viability of encapsulated and non-encapsulated cells after incubation in culture medium for different time periods. Viability was analyzed by flow cytometry (gated on single cells or single beads/CD138^+^/live; gating strategy in Supplementary Fig. 2). (**b**) Detection of IgG in the culture supernatant of encapsulated (red) and nonencapsulated cells (dark gray) by ELISA at different time points. The dashed line indicates the sensitivity of the assay (absorbance corresponding to 1.56 ng/mL IgG). The solid lines denote the mean of two technical replicates. (**c**) IgG1 secretion of encapsulated and non-encapsulated cells over time was analyzed by flow cytometry. To determine the extent of cross-contamination between beads, the IgG1 signal of plasma cells (CD138^+^) was compared to the signal of empty beads or cells that lack the plasma cell marker (CD138^−^; gating strategy in Supplementary Fig. 3). The numbers in the top right corner indicate the percentage of IgG^+^ events in the CD138^+^ (red) and CD138^−^ (blue) populations (IgG^+^ gate indicated by a gray line). The number in the bottom right corner indicates the percentage of ‘true positives’ (percentage of IgG^+^ events originating from CD138^+^ based on the sum of IgG^+^ events from both CD138^+^ and CD138^−^ populations). The plots show a minimum of 10,019 (CD138^−^) and 2,533 (CD138^+^) events at 2% contour level. (**d**) Time course of ASC IgG secretion and background based on **c**. Left axis: percentage of IgG1-positive events in the CD138^+^ (red diamonds) and CD138^−^ (blue diamonds) populations for each analyzed time point. Right axis (gray diamonds): percentage of events in the IgG1 gate that originate from CD138^+^ and not from CD138^−^. Source data

With a capture system for secreted proteins at hand, we established an antibody discovery workflow with primary immune cells. To determine cell survival in the hydrogel and optimal duration of antibody secretion, we isolated mouse bone marrow plasma cells (CD138^+^) by positive magnetic selection and encapsulated them into BG-agarose. We found that the encapsulation process did not affect the viability of CD138^+^ cells and that cells remained highly viable (92%) in the hydrogel even 4 h after encapsulation (Extended Data Fig. 3a). To establish the time window for secretion, we functionalized the BG-agarose hydrogel with anti-mouse κ VHH–SNAP and monitored the immunoglobulin G1 (IgG1) secretion signal over time by flow cytometry (Extended Data Fig. 3c,d). We found that the percentage of IgG-secreting plasma cells increased over time, while the background IgG signal of beads that did not contain a plasma cell (CD138^−^) increased only considerably after 1 h and 45 min of incubation. Non-encapsulated CD138^+^ cells were IgG1-negative because mouse bone marrow plasma cells do not express surface IgG^34^. We measured IgG levels in the supernatant by ELISA and found that the antibody levels generated by encapsulated cells slightly increased over time compared to background but remained outside of the quantifiable range of the assay and were considerably lower than in the supernatant of not encapsulated cells (Extended Data Fig. 3b). Based on the secretion time course data, we believe that the method is robust for a time window of 30 min to 1 h and 45 min as it is possible to identify a distinct IgG^+^ population in these samples while still maintaining a low level of background. For antibody selections, we chose 1 h and 45 min as this longer secretion time enables the detection of a larger proportion of ASCs while still being short enough to fit the antibody selection experiment into a single day.

**Extended Data Fig. 4:**
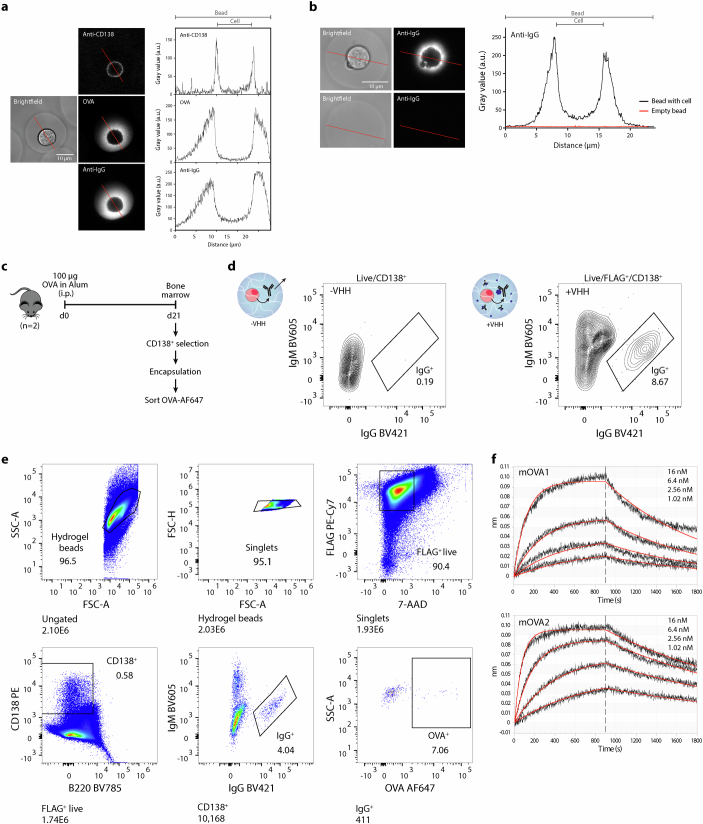
Generation of anti-OVA antibodies from encapsulated mouse bone marrow plasma cells and analysis of confocal images. (**a**) Analysis of confocal microscopy image of an encapsulated OVA-specific bone marrow plasma cell showing the spatial distribution of CD138 (AF647), IgG (AF405) and OVA (AF555) within the VHH-functionalized BG-agarose bead. The graphs display the pixel intensity along the red line from left to right. IgG and OVA signal intensity decrease as a function of distance from the encapsulated cell. A representative image of over 40 images obtained from two biological replicates is shown. Additional confocal images can be found in Supplementary Fig. 4. (**b**) Comparison of the IgG signal distribution of a hydrogel bead containing a cell with a neighboring empty bead. The cross-section for analysis is indicated by a red line. A representative image from two biological replicates is shown. Additional confocal images can be found in Supplementary Fig. 4. (**c**) Mouse immunization and analysis scheme. Bone marrow plasma cells (CD138^+^) were magnetically enriched. Cells were encapsulated into VHH-functionalized BG-agarose. OVA-specific plasma cells were sorted with fluorescently labeled monomeric OVA (OVA-AF647). (**d**) Comparison of IgG signal originating from plasma cells in BG-agarose beads incubated with (+VHH) or without VHH–SNAP–FLAG (−VHH; different experiment than shown in Fig. 1). The plots show 527 (−VHH) and 1,280 (+VHH) events at 2% contour level. (**e**) Representative gating strategy for identifying OVA-specific plasma cells from bone marrow. Live plasma cells inside hydrogel beads were gated as live/FLAG^+^/CD138^+^ (VHH–SNAP contains a FLAG tag). (**f**) Determination of binding affinity of mOVA1 and mOVA2 by biolayer interferometry: mOVA1: K_D_ = 2.36 ± 0.02 nM, χ^2^ = 0.0705, R^2^ = 0.985. mOVA2: K_D_ = 0.684 ± 0.004 nM, χ^2^ = 0.0388, R^2^ = 0.9919. Tested OVA concentrations: 1.02–16 nM.

We then immunized mice with hen egg ovalbumin (OVA) to obtain OVA-specific B cells (Extended Data Fig. 4). To visualize secretion and capture of OVA-specific antibodies, we stained cells compartmentalized into VHH-functionalized hydrogels with fluorescently labeled antibodies against CD138, secondary antibodies against IgG and fluorescently labeled OVA. By confocal fluorescence microscopy, we observed colocalization of IgG and OVA signal in the hydrogel around the cell, indicating secretion of OVA-specific IgG (Fig. 1d). The IgG and OVA signal distribution in the bead and the lack of IgG signal on empty beads suggest no substantial cross-contamination (Extended Data Fig. 4a,b and Supplementary Fig. 4).

To obtain OVA-specific antibody sequences for recombinant expression, we encapsulated bone marrow-derived plasma cells for sorting by FACS (Extended Data Fig. 4c). After incubation, hydrogel beads were stained with fluorescently labeled monomeric OVA, antibodies against B cell and ASC surface markers (B220 and CD138), an anti-FLAG antibody for labeling of VHH-functionalized hydrogels, as well as anti-IgG and IgM secondary antibodies. We hypothesized that in the absence of VHH, secreted antibodies are removed during washing steps due to the low background binding and large pore size of the agarose. Only samples to which VHH–SNAP had been added contained an IgG^+^ population, confirming VHH-specific retention of antibodies and excluding the staining of cell-surface Igs (Extended Data Fig. 4d).

**Extended Data Fig. 5:**
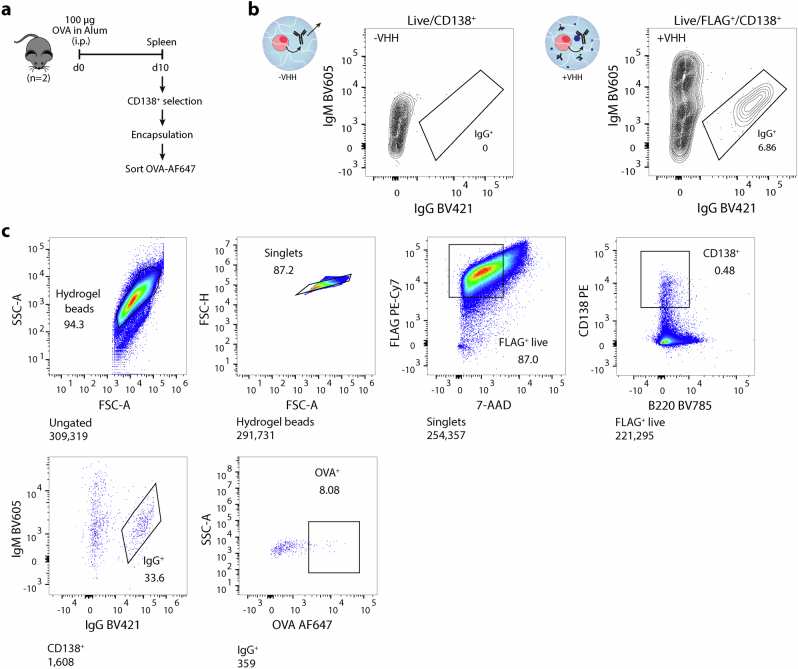
Workflow with spleen-derived ASCs. (**a**) Mouse immunization and analysis scheme. (**b**) Comparison of IgG signal originating from ASCs in BG-agarose beads incubated with (+VHH) or without VHH–SNAP–FLAG (−VHH; different experiment than shown in **c**). The plots show 3,663 (−VHH) and 2,290 (+VHH) events at 2% contour level. (**c**) Representative gating strategy for identifying OVA-specific ASCs from spleen. Live ASCs inside hydrogel beads were gated as live/FLAG^+^/CD138^+^ (VHH–SNAP contains a FLAG tag).

Plasma cells secreting OVA-specific antibodies (Fig. 1e and representative gating strategy in Extended Data Fig. 4e) were sorted into single wells of 96-well plates, and antibody variable genes were obtained by RT–PCR followed by Sanger sequencing. We recombinantly expressed two antibodies and found that they both bound OVA with high affinity using biolayer interferometry (BLI) (*K*_*D*_ values of 2.36 nM and 0.68 nM, respectively; Fig. 1f and Extended Data Fig. 4f), suggesting that high-affinity binders can be identified by our workflow. In addition to bone marrow-derived plasma cells, we also analyzed OVA-specific spleen-derived ASCs (Extended Data Fig. 5). With this setup, we can therefore screen millions of cells in a matter of hours and generate high-affinity mAbs from primary mouse immune cells.

### Direct discovery of mouse anti-SARS-CoV-2 receptor-binding domain (RBD) antibodies

**Fig. 2:**
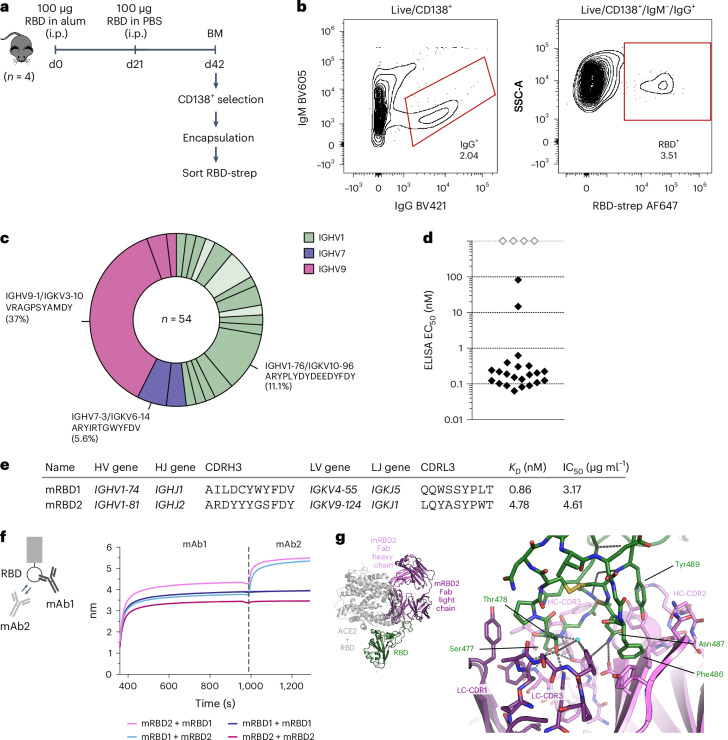
Generation of mouse anti-SARS-CoV-2 RBD antibodies. **a**, Mouse immunization and analysis scheme. Bone marrow plasma cells (CD138^+^) were magnetically enriched and then used in our workflow. RBD-specific plasma cells were sorted with fluorescently labeled RBD-streptavidin tetramers. **b**, Sorting of RBD-specific mouse plasma cells. Cells were gated as live/CD138^+^, and IgG-secreting RBD-specific plasma cells were sorted by FACS. The plots show 79,629 (CD138^+^) and 1,623 (IgG^+^) events at 2% contour level. **c**, Overview of antibody sequences of sorted plasma cells. In total, 54 paired heavy- and light-chain sequences were obtained. The pie chart shows the 21 observed HV and LV gene combinations (HV–LV). HV–LV pairings are colored by the HV gene. Combinations that were characterized are shown in darker shades, while combinations that were not expressed are shown in lighter shades. The three most expanded expressed HV–LV combinations are highlighted with their CDRH3 amino acid sequence and frequency. **d**, Summary of anti-RBD ELISA. The plot shows the EC_50_ with an antibody concentration range of 0.0002–400 nM. Antibodies that did not bind RBD at 400 nM are shown at an arbitrary EC_50_ of 1,000 nM (gray diamonds). **e**, Characteristics of mouse anti-SARS-CoV-2 RBD antibodies with neutralizing capacity—variable domain sequences (V and J genes), third complementarity-determining region amino acid sequences (CDR3), equilibrium dissociation constants (*K*_*D*_) and IC_50_ against WT SARS-CoV-2. **f**, In-tandem epitope binning experiment with mRBD1 and mRBD2. **g**, Crystal structure of mRBD2 with SARS-CoV-2 RBD (PDB: 8BE1). Top left, the RBD (green) in complex with mRBD2 Fab fragment (purple and pink for light and heavy chains) is superimposed with RBD complexed with ACE2 (gray; PDB: 6M0J), showing how the Fab fragment overlaps significantly with ACE2. The main figure shows details of the RBD loop (green carbon atoms) binding to the CDRs of the mRBD2 Fab fragment. Source data

**Extended Data Fig. 6:**
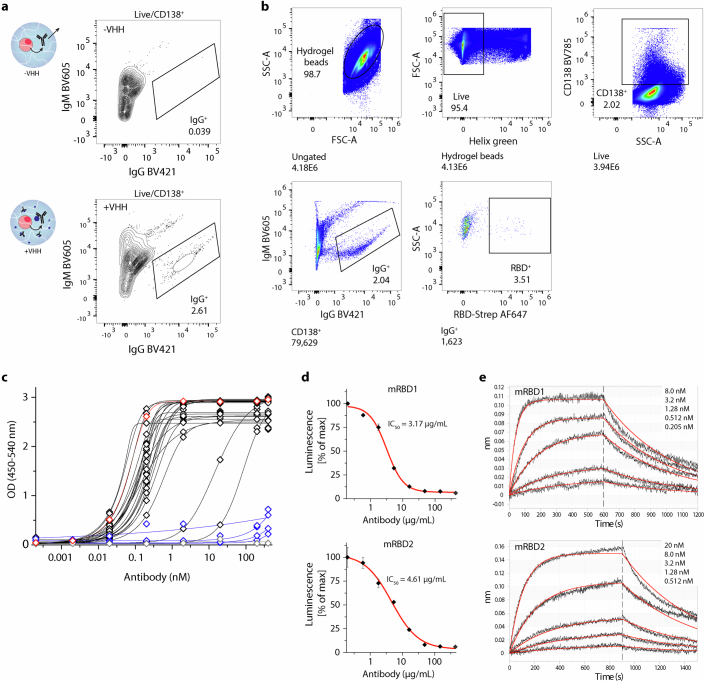
Sorting of mouse RBD-specific plasma cells and characterization of anti-RBD antibodies. (**a**) Comparison of IgG signal originating from plasma cells in BG-agarose beads incubated with (+VHH) or without VHH–SNAP (−VHH; different experiment than shown in Fig. 2). The plots show 2,536 (−VHH) and 14,606 (+VHH) events at 2% contour level. (**b**) Representative gating strategy for identifying RBD-specific plasma cells from mouse bone marrow (gated as live/CD138^+^/IgM^−^/IgG^+^/RBD^+^). (**c**) Anti-RBD ELISA. Workflow-derived antibodies are shown in black (binders) or blue (non-binders), a commercial positive control (clone 1035753, R&D Systems) is shown in red and an isotype control (mouse IgG1k, clone MG1-45; BioLegend) is shown in gray. The plot shows the mean of two technical replicates, representative of two independent experiments. (**d**) Neutralization of SARS-CoV-2 by mRBD1 and mRBD2. Wild-type SARS-CoV-2 (MOI = 0.01) was pre-incubated with a 3-fold dilution series of each antibody and then used to infect luminescent reporter cells. Levels of infection after 24 h were quantified as % of maximum luminescence. Mean values ± SD of three technical replicates are shown, representative of three independent experiments. IC_50_, half-maximal inhibitory concentration. (**e**) Determination of binding affinity of mRBD1 and mRBD2 by biolayer interferometry: mRBD1: K_D_ = 0.864 ± 0.07 nM, χ^2^ = 0.0547, R^2^ = 0.991. mRBD2: K_D_ = 4.78 ± 0.04 nM, χ^2^ = 0.1111, R^2^ = 0.9928. Source data

We then assessed our ability to isolate high-affinity antibodies against a relevant pathogen antigen. We immunized mice with the RBD of the wild-type (WT) SARS-CoV-2 spike protein, enriched plasma cells from bone marrow and used them in our workflow (Fig. 2a). Plasma cells secreting RBD-specific IgG antibodies were sorted with fluorescently labeled RBD-streptavidin tetramers into single wells of 96-well plates for Sanger sequencing (Fig. 2b and gating strategy in Extended Data Fig. 6b). Secreted antibodies could be identified by comparing the IgG signal of samples with and without VHH addition (Extended Data Fig. 6a).

We found 54 antibody sequences, belonging to 21 different heavy variable (HV) and light variable (LV) gene pairs (Fig. 2c) and representing 20 distinct VH clonotypes (Methods). Within this diversity, there were five highly expanded clones (≥3 members), including an *IGHV9-1* clone (20 members, 37% of all, representative CDRH3 VRAGPSYAMDY) and an *IGHV1-76* clone (7 members, 13% of all, representative CDRH3 ARYPLYDYDEEDYFDY). This observation of exceptional clonality among an antigen-specific plasma cell-derived population of antibodies is consistent with a recent mass-spectrometry-based proteomic study of resting and postinfection human IgG antibody repertoires^11^ and highlights that our method can offer insight into the most dominant antibodies in an organism’s active antigen response. Clonal comparisons to the Coronavirus Antibody Database (CoV-AbDab)^35^ revealed that two of our antibodies belonged to the same VH clonotype as antibodies from a separate survey^36^ into the mouse lymph-node-derived plasmablast response to SARS-CoV-2 spike (mRBD1 clustered with 2C02 and 2D01 and mRBD10 clustered with 1A10), suggesting that immunodominance can be detected among ASC-derived antibodies. Compared to the surface Ig-based sorting of plasma cell precursors^36^, our study adds insight into the secreted antibody response by fully differentiated plasma cells.

To confirm SARS-CoV-2 RBD binding and perform further functional profiling, we recombinantly expressed 26 representative antibodies as full-length mouse IgG1 (Supplementary Table 1 and full-length sequences in Supplementary Table 2). In an indirect ELISA against RBD, we found that 22 antibodies (84.6%) bound RBD at the tested concentrations (up to 400 nM antibody; Fig. 2d and Extended Data Fig. 6c), including the representatives of all five of the most expanded clones.

To determine whether the RBD-specific binders were also able to neutralize SARS-CoV-2, we used luminescent reporter cells to perform live virus neutralization assays^37–39^. Despite the high apparent affinity of our plasma cell clones, only two antibodies (mRBD1 and mRBD2 (belonging to the fifth most expanded clone)) were able to neutralize WT SARS-CoV-2 with an IC_50_ of 3.17 µg ml^−1^ and 4.61 µg ml^−1^, respectively (Fig. 2e and Extended Data Fig. 6d), highlighting that selection of an antibody in an immune response does not guarantee neutralizing capacity.

To further characterize mRBD1 and mRBD2, we determined their binding affinities to RBD (Extended Data Fig. 6e) and performed in-tandem epitope binning assays by BLI (Fig. 2f). The binning assay indicated that the two antibodies do not share the same binding site on RBD, as an increase in BLI signal was observed when antibodies were added sequentially to immobilized RBD.

To provide further insights into the binding mechanism of mouse ASC-derived RBD-binding antibodies^36,40^, we obtained a co-crystal structure of the Fab fragment of mRBD2 with RBD. The structure shows that mRBD2 binds to a long disulfide-bonded loop (residues 474–489) on the RBD that is part of the angiotensin-converting enzyme 2 (ACE2)-binding interface (Fig. 2g). In the antibody, the binding site is formed of CDR2 and CDR3 from both light and heavy chains. Phe487 and Tyr489 of the RBD interact with CDR2 of the heavy chain, and Asn487 is hydrogen bonded to both heavy and light chains. Ser477 hydrogen bonds to heavy-chain CDR3, while Thr478 hydrogen bonds to light-chain CDR3. The conformation of the RBD loop itself is almost identical to that seen in the ACE2:RBD complex while moving through a hinge-like motion by a few Ångström relative to the rest of the RBD (Supplementary Fig. 5). Comparison of mRBD2 with the ACE2-blocking mouse plasmablast-derived antibody 2B04 (ref. ^40^) showed that their binding sites on RBD partially overlap, hinting at similar neutralization mechanisms.

### Direct discovery of human anti-SARS-CoV-2 RBD antibodies

To demonstrate that our technology has utility in pandemic response efforts, we adapted our protocol to isolate antibodies directly from humans. mAbs derived from humans after infection or vaccination have been shown to be efficacious and well-tolerated in the human body, representing a fast track to therapeutic development^3^. Furthermore, studying antigen-specific plasmablasts gives unique insights into the postinfection and vaccine responses of different individuals^41,42^.

**Extended Data Fig. 7:**
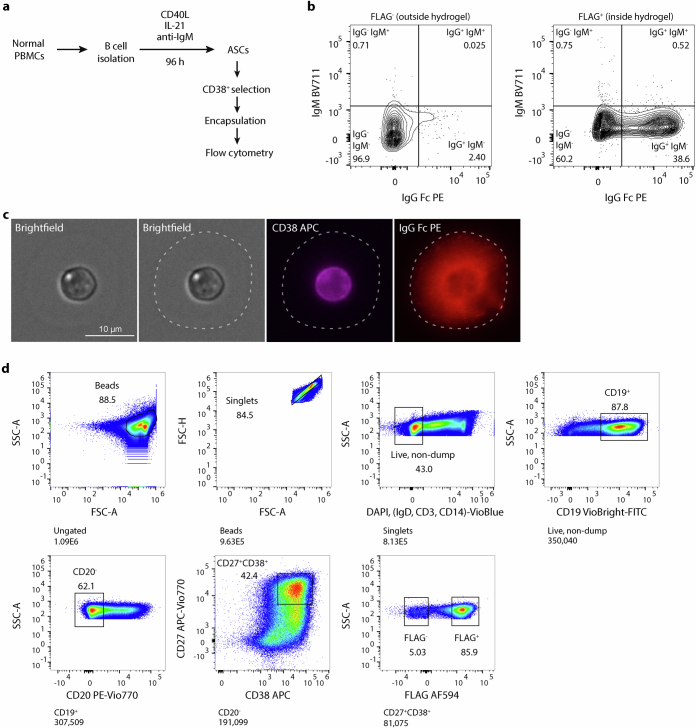
Stimulation of normal human PBMCs into ASCs and analysis of antibody secretion by flow cytometry and microscopy. (**a**) Stimulation conditions and workflow for proof-of-concept in normal PBMCs. To obtain ASCs from normal PBMCs, B cells were stimulated with CD40L, IL-21 and anti-IgM antibodies. (**b**) IgG secretion of stimulated PBMCs encapsulated into hydrogel beads. The plot shows 66,173 events at a contour level of 2%; the gating strategy is shown in **d**. (**c**) Analysis of encapsulated stimulated PBMCs by epifluorescence microscopy. Hydrogel beads were stained with fluorescently labeled anti-CD38 (APC) and anti-human IgG antibodies (PE). The hydrogel bead boundary is shown as a dotted line. A representative image from one independent experiment is shown. (**d**) Gating strategy for identifying IgG-secreting human ASCs from stimulated B cells. ASCs were gated as DAPI^−^/CD14^−^/CD3^−^/IgD^−^/FLAG^+^/CD19^+^/CD20^−^/CD27^+^/CD38^+^ (VHH–SNAP contains a FLAG tag).

To adjust the workflow for the capture of human antibodies, we exploited the modular setup of the capture system and exchanged the anti-mouse VHH with anti-human κ and λ VHHs^27^. First, we activated normal peripheral blood mononuclear cells (PBMCs) in vitro to stimulate antibody secretion^43^ and were able to detect antibody secretion and capture from single cells (Extended Data Fig. 7). The compatibility of the workflow with ex vivo-activated cells highlights the versatility of the technology and expands the scope of the method to the analysis of activated memory B cells.

**Fig. 3:**
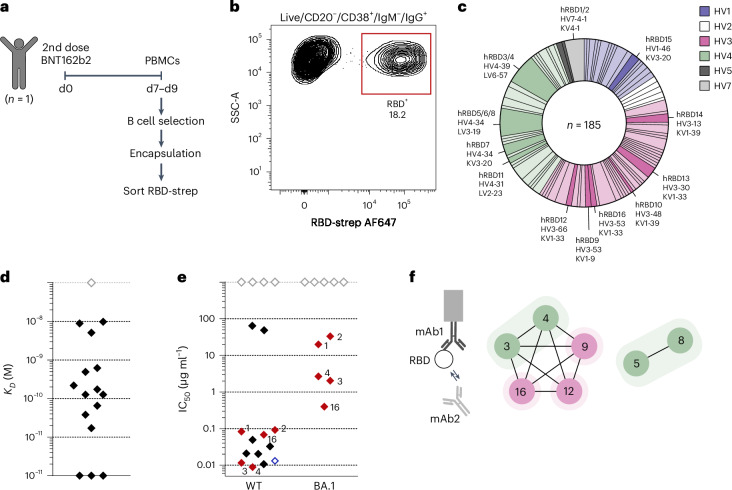
Generation of human anti-SARS-CoV-2 RBD antibodies. **a**, Human anti-RBD antibody discovery. B cells were negatively selected from fresh or frozen PBMCs 7–9 days after the second BNT162b2 vaccine dose and then used in the workflow. RBD-specific ASCs were sorted with fluorescently labeled RBD-streptavidin tetramers. **b**, Sorting of RBD-specific human ASCs. Representative layout for sorting of IgG^+^/RBD^+^ hydrogel beads obtained from the encapsulation of fresh B cells on d9 post vaccination. Hydrogel beads were gated as live/CD20^−^/CD38^+^/IgM^−^/IgG^+^. The plot shows 1,484 events at 2% contour level. **c**, Overview of sequences of human ASCs sorted with RBD. In total, 185 paired heavy- and light-chain sequences were obtained. The pie chart shows the observed HV–LV combination colored by the HV gene. HV–LV combinations that were expressed recombinantly are highlighted with their clone names and shown in a darker shade. **d**, Affinities (*K*_*D*_) of human anti-RBD antibodies. One antibody (gray diamond) did not show binding to RBD at the tested concentrations. **e**, Neutralization of WT and Omicron BA.1 SARS-CoV-2 by human anti-RBD antibodies. IC_50_ was calculated from experiments performed in duplicate. Antibodies with no quantifiable neutralizing capacity at the highest concentration tested (100 μg ml^−1^) are shown at an arbitrary IC_50_ of 1,000 μg ml^−1^ (gray diamonds). The ten antibodies with the lowest IC_50_ against WT SARS-CoV-2 were also tested against Omicron BA.1 SARS-CoV-2. Antibodies neutralizing both variants are highlighted in red with their clone names. Neutralization of WT SARS-CoV-2 by the REGEN-COV (Ronapreve) mAb cocktail (casirivimab and imdevimab) was tested in the same assay (blue diamond). **f**, Sandwich epitope binning experiment with pairs of neutralizing human anti-RBD antibodies. In the network plot, the nodes show the antibody clones, the connections indicate pairwise blocking and the shaded areas indicate whether the antibodies belong to the same clonotype. Colors indicate the VH gene as in **c**. Source data

**Extended Data Fig. 8:**
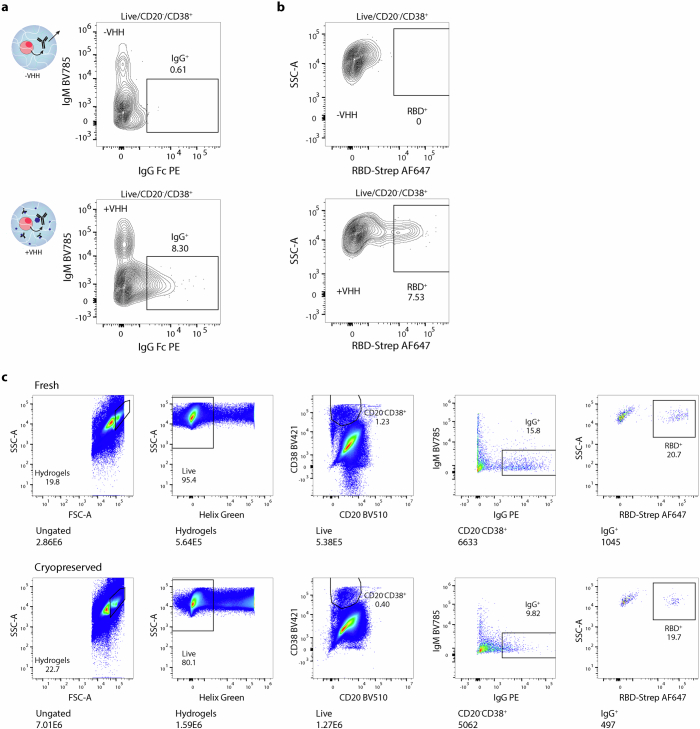
Sorting of SARS-CoV-2 RBD-specific human ASCs. (**a**) Comparison of IgG signal originating from cryopreserved ASCs on d7 post vaccination in BG-agarose beads incubated with (+VHH) or without VHH–SNAP (−VHH; different experiment than shown in Fig. 3). The plots show 1,139 (−VHH) and 1,289 (+VHH) events at 2% contour level. (**b**) Comparison of RBD-specific signal of cryopreserved ASCs on d7 post vaccination in BG-agarose beads incubated with (+VHH, secreted antibodies) or without VHH–SNAP (−VHH, surface antibodies). The plots show 1,139 (−VHH) and 1,289 (+VHH) events at 2% contour level. (**c**) Representative gating strategy for identifying RBD-specific human ASCs (gated as live/CD20^−^/CD38^+^/IgM^−^/IgG^+^/RBD^+^) and comparison of fresh and cryopreserved samples (day 8 post vaccination). Cells in hydrogels could be easily identified by forward and side scatter; therefore, the gating is different compared to other experiments.

Next, we identified SARS-CoV-2 RBD-specific antibodies directly from human antibody-secreting plasmablasts. We isolated PBMCs 7–9 days after the second dose of the mRNA vaccine BNT162b2 and enriched for B cells by negative selection (Fig. 3a). After encapsulation into BG-agarose and functionalization with anti-human κ and λ VHH–SNAP, we observed a distinct IgG-secreting, RBD-positive ASC population (Fig. 3b and Extended Data Fig. 8a,c). We then compared the antigen-specific signal of all plasmablasts in the sample with added VHH (enabling analysis of secreted antibodies) with the same sample incubated and stained in the same way but without the capture construct (−VHH, only RBD-specific antibodies on the cell surface can be investigated; Extended Data Fig. 8b). We did not observe an antigen-specific plasmablast population (RBD^+^) in the sample without capture reagent, providing evidence that the antibodies we discovered could not have been identified by FACS surface staining alone under these experimental conditions. Our results do not exclude the possibility that cells with surface Ig that specifically bind to RBD occur in the population, but our approach specifically targets ASCs and would thus not retain them a priori.

We sorted IgG-secreting RBD-positive cells from fresh or cryopreserved samples into single wells of 96-well plates for sequencing. Compared to freshly isolated PBMCs, ASC recovery and IgG secretion from cryopreserved samples were decreased, but no difference in the frequency of RBD-specific ASCs was observed (Extended Data Fig. 8c). Reduced ASC recovery from cryopreserved samples compared to fresh samples has been reported in the literature^44^.

From just one individual, we obtained 185 plasmablast sequences, belonging to a broad spread of HV–LV gene pairs (Fig. 3c). The 185 antibodies spanned 156 different VH sequences and 111 different VH clonotypes (Methods). As in the mouse study, we observed a population of extremely expanded VH clonotypes. Fifteen clonotypes had occupancy of ≥3 members, including an *IGHV4-34* clone (10 members, 5.4% of total, representative CDRH3 ARAHLIGDCGGGSCYSGPDPSNWFDP), an *IGHV4-39* clone (8 members, 4.3% of total, representative CDRH3 ARRRAGSYFKDLFDY) and an *IGHV7-4-1* clone (8 members, 4.3% of total, representative CDRH3 ARVGRAAIAALDDAFDI).

Clonal clustering against the thousands of human SARS-CoV-2 response antibodies in CoV-AbDab showed that 17 (9%) of our plasmablast sequences belonged to the same VH clonotype as antibodies already characterized to be a SARS-CoV-2 RBD binder^35^. Several of these belonged to immunodominant VH clonotypes based on their identification across numerous independent studies on alternative B cell compartments from SARS-CoV-2 convalescent patients (such as *IGHV3-53* with representative CDRH3 ARDLGEAGGMDV—four sequences in our study and six sequences from six other studies as of February 2022 (refs. ^45–50^)). This clustering demonstrates the presence of RBD-reactive bulk B cell and memory B cell clones among a postvaccination ASC population, confirming their differentiation/expansion as part of the active immune response against SARS-CoV-2.

Additionally, we performed Fv region structural clustering of our sequences alongside CoV-AbDab using the Structural Profiling of Antibodies to Cluster by Epitope (SPACE) protocol^51^, which clusters anti-coronavirus antibodies by predicted structure and can group them into sets that bind the same antigenic site. This enables an orthogonal consideration of immunodominant epitopes because antibodies that have the same topology but belong to a different VH clonotype can recognize the antigen with the same binding mode. SPACE yielded an additional 13 shared clusters for CoV-AbDab, highlighting cases where the same epitope was likely to be targeted, although the sequences did not belong to the same clonotype.

We then expressed 16 antibodies that either belonged to the most prevalent clonotypes not yet present in the CoV-AbDab (hRBD1-8, belonging to three different clonotypes) or were implicated in RBD binding based on SPACE structural clustering with antibodies found in the CoV-AbDab (hRBD9-16, belonging to eight different clonotypes) as full-length human IgG1 (Supplementary Table 3 and full-length sequences in Supplementary Table 4). Of these antibodies, 94% (15/16) bound RBD at the tested concentrations with affinities in the low picomolar to nanomolar range (Fig. 3d). Of the RBD binders, 80% (12/15) were also able to neutralize WT SARS-CoV-2. Among these, ten antibodies showed high neutralizing capacity, with IC_50_ values comparable to the REGEN-COV (Ronapreve) mAb cocktail (range approximately 10–100 ng ml^−1^; Fig. 3e). As observed for many other antibodies induced by WT SARS-CoV-2 (refs. ^52,53^), neutralizing capacity against Omicron BA.1 SARS-CoV-2 was generally reduced. Nonetheless, it was still quantifiable for five of our antibodies (Fig. 3e), and one antibody (hRBD16) retained most of its neutralizing capacity (IC_50_ of 68 ng ml^−1^ against WT compared to 398 ng ml^−1^ against Omicron BA.1). hRBD16 is highly homologous to CAB-B37, a memory B cell-derived antibody that can neutralize a wide range of SARS-CoV-2 variants^54^, suggesting that hRBD16 might also be broadly neutralizing. We used epitope binning by BLI to further characterize seven strongly neutralizing antibodies and determined that they bound to two distinct competition regions on WT RBD (hRBD3/4/9/12/16 and hRBD5/8 bound to the same regions; Fig. 3f). Conversely, the differences in neutralizing capacity against Omicron BA.1 (hRBD3/4/16 neutralized Omicron BA.1, while hRBD9/12 did not) indicate that although these antibodies bind to the same region on the RBD, they interact with different residues.

### Selection of specific binding sites during ASC screening

**Fig. 4:**
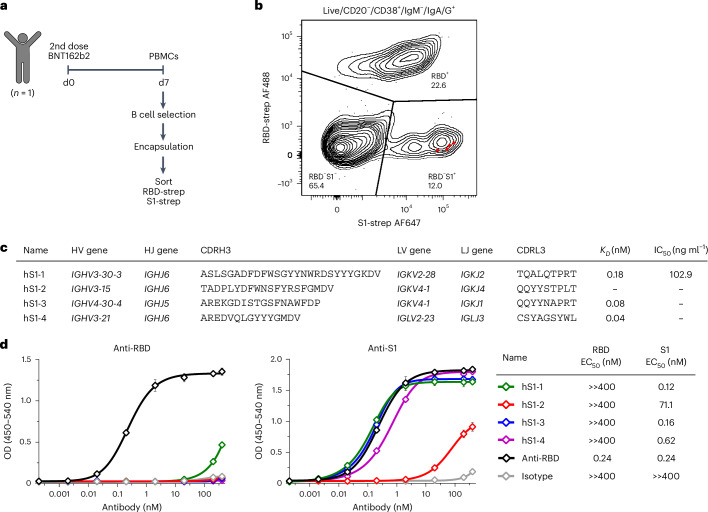
Generation of human anti-SARS-CoV-2 S1 antibodies that do not bind the RBD. **a**, Human anti-S1 antibody discovery. B cells were negatively selected from PBMCs 7 days after the second BNT162b2 vaccine dose and then used in the workflow. RBD/S1-specific ASCs were sorted with fluorescently labeled RBD- and S1-streptavidin tetramers. **b**, Index sorting of S1-specific human ASCs. Layout for sorting of IgG- or IgA-secreting ASCs isolated on day 7 post vaccination with fluorescently labeled S1- and RBD-streptavidin tetramers. Based on index sorting, the ratio of the S1/RBD fluorescence signal was calculated for all sequenced cells, and four sequences that corresponded to events with the highest S1/RBD fluorescence signal ratio were selected for expression (highlighted as red squares). The plot shows 719 events at a 5% contour level, and the percentage of events in each window is indicated. **c**, Characteristics of human anti-S1 antibodies—variable domain genes (V and J), third complementarity-determining region amino acid sequences (CDR3), equilibrium dissociation constants (*K*_*D*_) and IC_50_ against WT SARS-CoV-2. En dashes (–) denote that binding or inhibition was not quantifiable at the tested concentrations. **d**, Anti-S1 and anti-RBD ELISA of anti-S1 antibodies. An RBD-binding positive control (human IgG1κ, clone AM001414; BioLegend) is shown in black, and an isotype control (human IgG1κ, clone QA16A12; BioLegend) is shown in gray. The table shows the EC_50_ with an antibody concentration range of 0.0002–400 nM. The plots show mean values ± s.d. of two independent experiments performed in duplicate. Source data

**Extended Data Fig. 9:**
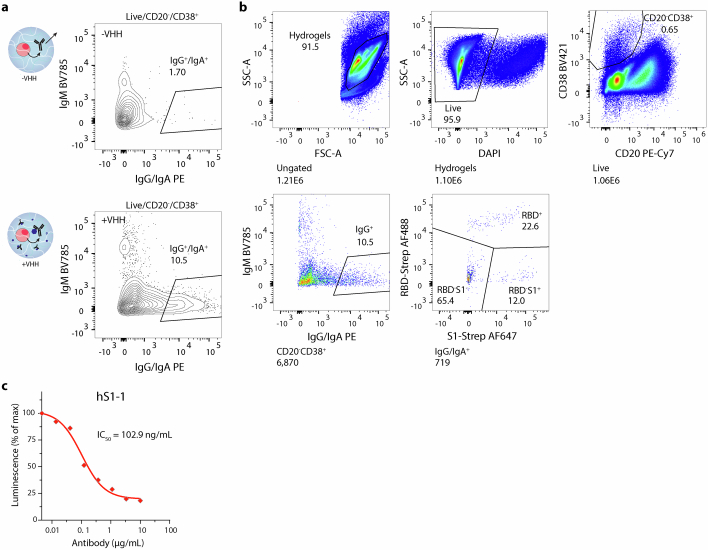
Sorting of SARS-CoV-2 S1-specific human ASCs. (**a**) Comparison of IgG signal originating from ASCs in BG-agarose beads incubated with (+VHH) or without VHH–SNAP (−VHH). The plots show 823 (−VHH) and 6,870 (+VHH) events at 2% contour level. (**b**) Representative gating strategy for identifying S1/RBD-specific human ASCs. (**c**) Neutralization of SARS-CoV-2 by hS1-1. Wild-type SARS-CoV-2 (MOI = 0.01) was pre-incubated with a 3-fold dilution series of hS1-1 and then used to infect luminescent reporter cells. Levels of infection after 24 h were quantified as % of maximum luminescence. Mean values of two technical replicates are shown, representative of two independent experiments. IC_50_, half-maximal inhibitory concentration. Source data

To take advantage of the multiplexed readout of FACS, we then aimed to select specific binding properties detectable during the single-cell screen. As a proof of concept, we isolated antibodies that bind to the S1 region of the spike protein but do not bind to the RBD. We encapsulated B cells isolated from PBMCs 7 days after the second BNT162b2 dose and stained hydrogel beads with fluorescently labeled S1-streptavidin and RBD-streptavidin (Fig. 4a). We observed a distinct S1-positive but RBD-negative population and sorted and sequenced all antigen-specific IgG- or IgA-positive ASCs (Fig. 4b and secretion control/gating strategy in Extended Data Fig. 9a,b). We selected four sequences that showed the largest S1/RBD FACS signal ratio for expression as full-length human IgG1 (highlighted as red squares in Fig. 4b and sequence information in Supplementary Tables 3 and 4). We were able to obtain the binding affinities of three antibodies and found that they bound S1 with affinities in the picomolar range (Fig. 4c). One of the antibodies (hS1-1) was able to neutralize WT SARS-CoV-2 with an IC_50_ of 102.9 ng ml^−1^ (Fig. 4c and Extended Data Fig. 9c) but did not show neutralizing capacity against the Omicron BA.1 variant (Supplementary Fig. 7). We verified binding of all antibodies to S1 but not RBD by ELISA (Fig. 4d), suggesting that we successfully selected for S1 but not RBD binding in our screen. These results highlight the advantages of the multiplexed, FACS-based readout as it enables us to preselect relevant candidates (for example, binders to specific epitopes or multiple viral variants) and focus attention on the downstream characterization of antibodies with specific properties rather than investing resources in the expression of many potentially unsuitable candidates.

## Discussion

Our work establishes a fast, simple, high-throughput approach for the selection of functional, high-affinity antibodies from an underexplored B cell compartment. We generated antibodies directly from ASCs without the need to prepare a DNA library, using straightforward microfluidic equipment and a commercial flow cytometer. Antibodies are screened in the full-length IgG format, and functional characterization can be achieved as quickly as 2 weeks after obtaining samples by a single researcher without automation. We have illustrated the versatility of this approach by analyzing different species (mouse and human) and cells from different tissues (bone marrow plasma cells, splenic ASCs, ex vivo-stimulated PBMCs and PBMCs post vaccination).

**Extended Data Fig. 10:**
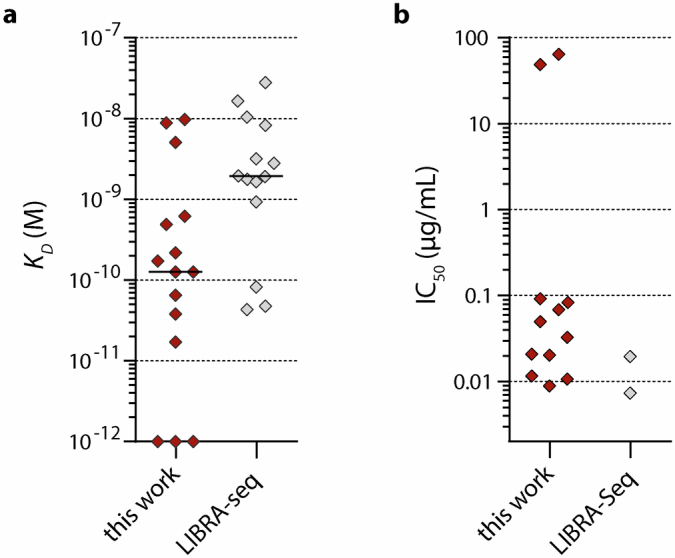
Comparison of human anti-SARS-CoV-2 RBD antibodies isolated in this work with antibodies isolated by LIBRA-seq. (**a**) Comparison of affinities (K_D_). All characterized RBD binders were included in the comparison. For antibodies isolated in this work, the same K_D_ values are depicted in Fig. 3d. Antibody affinity measurements by BLI were performed once but using at least four different analyte concentrations. For antibodies isolated by LIBRA-seq^55^, previously reported values are shown. Horizontal lines denote the median. (**b**) Comparison of neutralizing capacities. Only antibodies tested against authentic SARS-CoV-2 were included in the comparison. For antibodies isolated in this work, the same IC_50_ is depicted in Fig. 3e. Mean values of two technical replicates are shown. For antibodies isolated by LIBRA-seq, previously reported values are shown. Different neutralization assays were used in the two studies. Source data

Our antibody discovery campaigns showcase the ability of our technology to generate high-quality mouse and human antibodies. The best postvaccination antibodies we characterized have exceptionally high binding affinities (<10^−12^ M) and neutralizing capacities (10–100 ng ml^−1^) comparable to a clinical-stage antibody cocktail. Affinities and neutralizing capacities were also equivalent to those of memory B cell-derived antibodies isolated by linking B cell receptor to antigen specificity through sequencing (LIBRA-seq) after natural infection^55^, highlighting that the results obtained with our technology match those of an established protocol but provide insights into a compartment not yet studied with LIBRA-seq (Extended Data Fig. 10). Five of our human antibodies also showed neutralizing capacity against Omicron BA.1, demonstrating that cross-neutralizing activity can be isolated from the plasmablast population after vaccination against WT SARS-CoV-2. These binders were isolated from a single human donor without prior screening of serum or plasma for neutralizing capacity. In addition, our discovery campaigns were highly efficient, with a true positive rate of >85% for all antibodies and 95% for plasmablast-derived antibodies. In contrast, a recent study performing unbiased sorting of plasmablasts after BNT162b2 vaccination yielded on average 20.3% (42 binders of 206 expressed antibodies) of true hits^42^. By enabling antigen-specific sorting, our technology is therefore much more efficient than sorting based on cell-surface markers alone.

Antigen-specific sorting of human peripheral blood plasmablasts based on surface Ig has been performed previously^56^, but we believe that targeting secreted antibodies rather than relying solely on surface Ig staining is valuable in antibody discovery because a much larger proportion of the antigen-specific ASC repertoire can be accessed. Plasmablasts exhibit heterogeneity in surface Ig levels and, in particular, show little or no expression of surface IgG, the most relevant therapeutic antibody format^57–60^. The majority of plasmablasts (up to 80%)^57^ have lost Ig surface expression and can therefore not be selected based on cell-surface staining. Additionally, antigen-specific staining of downregulated surface Ig is less sensitive than staining of antibodies that are secreted in large quantities. Because only a small fraction of the antibody repertoire is accessible and the amount of primary human material is usually limited, screening a larger proportion of the population increases the chances of finding effective binders.

Leveraging the high throughput of FACS for secreted antibody screening enables the analysis of >10^7^ hydrogel beads per h, so even rare antigen-specific ASCs in heterogeneous cell populations can be identified. As highly potent and broadly protective binders can be rare, our high screening capacity increases the likelihood of isolating powerful drug candidates^1^. Another advantage of a flow-cytometry-based readout is that multicolor sorts are a standard procedure, enabling us to test binding to several different antigens or the detection of different antibody isotypes. We have demonstrated sorting with different antigens by selecting antibodies that bind to SARS-CoV-2 spike S1 but not the RBD. In the future, screening with several antigen variants could be used to identify antibodies against highly conserved epitopes, which increases the likelihood of isolating broadly neutralizing antibodies and can help define rational antigens for vaccination^61^. Furthermore, multicolor FACS enables counter-selection in the presence of inhibitors or competitors (such as ACE2)^55^ or screening for orthologue cross-reactivity for testing in animal models^62^.

More generally, this methodology could be used for antibody discovery outside of the context of infectious diseases, for example, by harnessing (humanized) mice for the development of therapeutic antibodies against cancer. Due to its high throughput, our technology could serve as a fast, economical and scalable alternative to hybridoma screening. Generation of hybridoma is time consuming (months) and is usually performed in microtiter plates, which have very low throughput and use more resources than our screening format^63^.

The concept of hydrogel-mediated capture of secreted proteins followed by FACS has been proposed before but has not been applied to the selection of functional, recombinant antibodies from natural immune responses^64–66^. Additionally, in contrast to a previously described microdroplet generation method that created polydisperse hydrogel beads of up to 55 µm in diameter^67^, controlled microfluidic encapsulation of cells creates smaller, monodisperse hydrogel beads, which facilitates sorting. The combination of single-cell microfluidic encapsulation with FACS also has advantages over methods solely based on microfluidics. Compared to fully microfluidic workflows, we offer a simpler alternative that achieves higher throughput without extensive setup or optimization. An advantage of microfluidic sorting is that custom software solutions enable real-time processing of the fluorescence signal time trace and therefore allow differentiation of signal localization, which can be used to select for specific binding properties such as binding to target cells^17^. However, commercial FACS instruments achieve sorting rates more than ten times higher than droplet microfluidic sorters previously used for antibody discovery (200–600 Hz)^17,18,68^ and enable more complex, multicolor sorts and retrospective identification of (antibody) phenotype through index sorting. The throughput of our method is also orders of magnitude higher than that of microchamber-based microfluidic systems (1,000–10,000s of cells per chip)^69–71^. While some of these approaches have delivered functional antibodies, they are less accessible to researchers owing to cost or proprietary protocols. Compared to methods that use cell-surface capture combined with FACS^32,33,72^, we propose a defined system in which we can control the amount of capture reagent independently of cell-surface molecules and without modifying the cell surface. For a given density of capture reagent, capture capacity in the 3D volume of the hydrogel is higher than on the 2D cell surface, enabling longer secretion times and therefore inclusion of slow secretors and better signal-to-noise ratios in populations with many highly secreting cells. For example, the average number of CD45 molecules available for CD45-mediated capture is 5 × 10^5^ on peripheral blood B cells (CD19^+^)^73^ compared to a capture capacity of up to 10^9^ in our system. The agarose hydrogel around the cell captures enough antibodies to be used with both monomeric (OVA) and tetrameric (RBD-streptavidin and S1-streptavidin) antigens without the signal amplification required in previously described methods^33^. Additionally, covalent cell-surface modification^32,72^ can impact cell viability and functionality in downstream assays.

Our technology can also offer insights into immunobiology. By facilitating access to the ASC compartment, we can now look into contrasting the affinities, variant specificities and biophysical properties of ASC-derived antibodies relative to those of other compartments. In this initial study, we have outlined how our antigen-specific plasmablast sequence profiles can be compared with anti-SARS-CoV-2 antibodies reported in studies on different individuals and B cell compartments to offer finer-grain insights into immunodominance—the phenomenon that underpins convergent viral mutation. This understanding can be used to prioritize candidates tactically, considering both their functional efficacy and their likelihood of withstanding viral natural selection pressures^74^.

Finally, the experimental framework introduced in our study can be extended to other secreted proteins, such as antibodies from other species^26^ or cytokines, because the modular design of our capture system (BG-agarose and SNAP-tag protein fusions) allows rapid adaptation. We hypothesize that light-chain-mediated capture, as used in our system, could also enable interrogation of other characteristics of the secreted antibodies, such as glycosylation^75^.

Our technology is currently limited by the throughput of cell encapsulation (up to 10^7^ cells per h) and low sequencing yield. Cell encapsulation can be further optimized by adjusting the mean number of cells per droplet and by running more microfluidic chips in parallel. While we have used full-length Sanger sequencing for simplicity, our workflow can easily be adapted to next-generation sequencing technologies^76,77^. We aim to increase sequencing output by optimizing single-cell sequencing protocols and by combining our sorting technology with the LIBRA-seq 10X Genomics workflow that uses DNA-barcoded antigens^23^, which should also yield single-cell transcriptomic datasets. These improvements will give us the ability to construct sequence-function landscapes for thousands of ASC-derived antibody sequences^78^.

In summary, we present a tool for rapid isolation of (human) mAbs that enabled the discovery of hundreds of antibodies within weeks and had a high recovery rate of true positives (>85%). Through this more efficient hit discovery, resources can be shifted from binder identification to more thorough experimental characterization and computational assessment^79^. Additionally, as disease outbreaks become more likely in the future^80^, this technology should increase our pandemic preparedness by enabling faster access to antibody reagents.

## Methods

### Ethics statement

Animal experiments were licensed by the UK Home Office according to the Animals Scientific Procedures Act 1986 (license PP6047951) and approved by local ethics committees from the University of Cambridge. Human sample collection and analysis were conducted in accordance with the principles of Good Clinical Practice and following approved protocols of the National Institute for Health and Care Research (NIHR) National Bioresource. Samples were collected with the written informed consent of all study participants under the NIHR National BioResource-Research Tissue Bank (NBR-RTB) ethics (REC: 17/EE/0025).

### Microfluidic methods

The microfluidic chip was designed using AutoCAD (Autodesk) and printed on a high-resolution photomask (Micro Lithography Services). The channel layout of the microfluidic chip (Supplementary Fig. 1) is available as a.dxf file from https://openwetware.org/wiki/DropBase, to manufacture a chip in-house or by outsourcing. Polydimethylsiloxane (PDMS) microfluidic chips were fabricated using standard soft lithography^81^. For replica molding, 20 g of PDMS (Sylgard 184; Dow Corning) monomer and curing agent were mixed at a ratio of 10:1, degassed, poured onto the master and solidified overnight at 65 °C. PDMS slabs were cut from the master, inlets and outlets were created using a 1 mm biopsy punch (Kai Medical) and the PDMS slab was permanently bonded to glass slides through plasma treatment (Diener Femto). After bonding, microfluidic channels were flushed with 1% (vol/vol) trichloro(1H,1H,2H,2H-perfluoro-octyl)silane (Sigma-Aldrich) in HFE-7500 (3M Novec) to make them more hydrophobic. Flow rates in microfluidic workflows were controlled using neMESYS 290N syringe pumps (Cetoni) and gas-tight syringes (Hamilton) connected to PTFE tubing (inner diameter 0.5 mm; BOLA). Droplet formation was monitored using a high-speed Mini UX-100 camera (Photron) mounted onto an inverted microscope (Brunel SP981 Microscope). In all experiments, 1% (wt/wt) RAN surfactant (RAN Biotechnologies) in HFE-7500 was used.

### Protein expression and purification

#### VHH and VHH–SNAP

Sequences of VHHs (TP1170 (ref. ^26^) as anti-mouse κ VHH, HuFab kappa 1 (refs. ^27,82^) as anti-human κ VHH and HuFab lambda 1 (refs. ^27,82^) as anti-human λ VHH) were obtained as gene fragments (Integrated DNA Technologies) and cloned into pET28a plasmids (Novagen) using Gibson assembly^83^ (see Supplementary Table 5 for amino acid sequences of VHH constructs). For the capture of mouse antibodies, we focused on anti-κ VHH, as 95% of mouse B cells express κ light chains^84^. Proteins were expressed in *E. coli* BL21(DE3) in 2xYT with 50 µg ml^−1^ kanamycin. Cultures were grown at 30 °C, protein expression was induced at an optical density at 600 nm (OD_600_) of 0.6–0.8 with 0.25 mM isopropyl β-D-1-thiogalactopyranoside (IPTG) and proteins were expressed for 5 h at 24 °C. After collection, the cell pellet was resuspended in His-wash buffer (50 mM Tris–HCl (pH 7.5), 300 mM NaCl and 20 mM imidazole) with a protease inhibitor (Protease Inhibitor Cocktail; cOmplete Roche) and 1 µl benzonase nuclease (Sigma-Aldrich). Cells were lysed using 10× BugBuster (Merck). Cleared lysate was added to a Ni-NTA column (Ni-NTA affinity resin; Generon) pre-equilibrated with His-wash buffer. Proteins were eluted with 50 mM Tris–HCl (pH 7.5), 300 mM NaCl and 500 mM imidazole. VHH constructs were not further purified and directly rebuffered into a storage buffer (10% glycerol in PBS) using PD-10 columns (GE Healthcare). To achieve higher purity, a reverse nickel column purification was performed with the VHH–SNAP constructs. Proteins were rebuffered into tobacco etch virus (TEV) protease cleavage buffer (25 mM Tris–HCl, pH 8.0, 150 mM NaCl) and subjected to overnight cleavage at 4 °C with His-tagged TEV protease (Sigma-Aldrich) according to the manufacturer’s instructions. Cleaved proteins were then rerun on a Ni-NTA column to remove the His-tagged TEV protease and the cleaved epitope tag. Flow-through and wash fractions were collected, and the buffer was exchanged for 10% glycerol in PBS using PD-10 desalting columns. Proteins were aliquoted, snap-frozen in liquid nitrogen and stored at −80 °C.

#### Production of SARS-CoV-2 RBD-CHis for immunization

Synthetic, codon-optimized DNA encoding the RBD of WT SARS-CoV-2 was cloned into the pExp-CHis vector, which introduces a C-terminal octa-His-tag (RBD-CHis; sequences in Supplementary Table 5). RBD-CHis protein was expressed in *E. coli* BL21(DE3) in 2xYT medium with 100 µg ml^−1^ ampicillin at 37 °C. Protein expression was induced at OD_600_ of 0.8–1.0 by the addition of 0.4 mM IPTG and progressed for 16 h. Cells were pelleted by centrifugation and lysed using Avestin EmulsiFlex C-5 homogenizer in 50 mM Tris–HCl (pH 8.0) and 5 mM EDTA supplemented with 250 µg ml^−1^ of DNase I. Inclusion bodies were isolated by centrifugation and washed first with 50 mM Tris–HCl (pH 8.0), 5 mM EDTA and 1% Triton X-100 and then with 50 mM Tris–HCl (pH 8.0), 1 M NaCl and 5 mM EDTA. Inclusion bodies were solubilized in 50 mM Tris–HCl (pH 8.0), 5 mM EDTA, 10 mM tris(2-carboxyethyl)phosphine (TCEP) and 6 M guanidine hydrochloride. For refolding, the protein solution was added slowly to 50 mM Tris–HCl (pH 8.5), 50 mM ethanolamine, 1 M pyridinium propyl sulfobetaine (PPS), 2 mM EDTA, 2 mM cysteine and 0.2 mM cystine and incubated for 96 h at 4 °C. The refolded protein was first purified by reverse-phase chromatography using a 10 ml source RPC column and applying a 10–90% acetonitrile/0.1% TFA gradient. The peak fractions were pooled, and the protein was lyophilized. Then the protein was dissolved in 20 mM sodium phosphate (pH 7.2), 300 mM NaCl and 0.5 M urea and further purified by size-exclusion chromatography using Superdex 200 Increase 10/300 GL column (Cytiva) equilibrated in 20 mM sodium phosphate (pH 7.2) and 300 mM NaCl.

#### Production of SARS-CoV-2 RBD for crystallization

DNA encoding WT SARS-CoV-2 RBD was cloned into the pExp-His-Zbasic plasmid to obtain a fusion of RBD with an N-terminal His-tag and Zbasic fusion partner followed by a TEV protease cleavage site (His-Zbasic-TEV-RBD; Addgene, plasmid 194999; sequence in Supplementary Table 5). The protein was expressed in *E. coli* BL21(DE3) in 2xYT media with 100 µg ml^−1^ ampicillin. Cells were grown to OD_600_ 0.8–1.0, and expression was induced with 0.4 mM IPTG. Cells were pelleted by centrifugation after 5 h of expression at 37 °C and resuspended in 20 mM Tris–HCl (pH 8.0) and 100 mM NaCl. Before lysis by sonication, 250 µg ml^−1^ of DNase I and 1 mM phenylmethylsulfonyl fluoride (PMSF) were added to the cells. The lysate was cleared by centrifugation, the supernatant was discarded and homogenized inclusion bodies were washed twice—first with 20 mM Tris–HCl (pH 8.0), 10 mM EDTA, 1 mM TCEP and 1 % Triton X-100, and then with 20 mM Tris–HCl (pH 8.0), 1 M NaCl and 1 mM TCEP. The pellet was dissolved in 20 mM Tris–HCl (pH 8.0), 200 mM NaCl, 10 mM imidazole, 1 mM TCEP and 6 M guanidine-HCl, and the insoluble material was removed by centrifugation. Solubilized, denatured protein was purified using a PureCube Ni-NTA agarose column. After loading the protein, the column was washed with ten column volumes (CVs) of 20 mM Tris–HCl (pH 8.0), 500 mM NaCl, 10 mM imidazole, 6 M deionized urea and 0.5 mM TCEP, and the protein was eluted with five CVs of 20 mM Tris–HCl (pH 8.0), 100 mM NaCl, 250 mM imidazole, 6 M deionized urea and 0.5 mM TCEP. The fusion protein was refolded by diluting it 1:20 in a dropwise fashion into refolding buffer (100 mM Tris, 500 mM Arg-HCl, 0.5 g l^−1^ polyethylene glycol (PEG) 3350, 1 M urea, 2 mM cysteine and 0.2 mM cysteine) with stirring at 4 °C. After 72 h of refolding, the solution was diluted twofold with 50 mM MES–NaOH (pH 6.0) and filtered through a Whatman GF/B microfiber filter. The clarified protein solution was loaded onto a HiTrap SP HP cation-exchange column (Cytiva). The column was washed with five CVs of 20 mM HEPES–NaOH (pH 7.2) and 50 mM NaCl and eluted with a 25 CV linear gradient to 20 mM HEPES–NaOH (pH 7.2) and 1,000 mM NaCl. Peak fractions were pooled, and NHis-TEV protease (produced in-house) was added overnight at 4 °C. Cleaved protein was passed through a Ni-NTA column to remove the His-Zbasic fusion partner, uncleaved fusion protein and NHis-TEV protease. RBD from the flow-through was concentrated using a 10 kDa MWCO Amicon Ultra concentrator and loaded onto HiLoad Superdex 75 pg 16/60 size-exclusion column (Cytiva) equilibrated with 20 mM Tris–HCl (pH 7.2) and 200 mM NaCl. Pooled peak fractions were concentrated, flash-frozen in aliquots and stored at −80 °C.

#### Production of biotinylated RBD

Synthetic DNA encoding for WT SARS-CoV-2 RBD was cloned into the pExp-His-Zbasic-Avi plasmid to result in a fusion of RBD with an N-terminal His-Zbasic-TEV fusion partner and a C-terminal Avi-tag (His-Zbasic-TEV-RBD-Avi; Addgene, plasmid 195000; sequence in Supplementary Table 5). The Avi-tagged RBD was produced following essentially the same procedure as untagged RBD except for the addition of 5 mM MgCl_2_, 2 mM ATP, 150 μM biotin and 50 μg ml^−1^ of biotin ligase (BirA-CHis, produced in-house) during the TEV protease cleavage step for the site-specific biotinylation at the Avi-tag. The reaction proceeded for 16 h at 22 °C with gentle mixing.

### Antigen tetramerization

For the streptavidin-RBD complex (strep-RBD) formation, a 10× molar excess of biotinylated RBD-Avi protein was incubated with 0.5 mg ml^−1^ of streptavidin-Alexa Fluor 488 or streptavidin-Alexa Fluor 647 (BioLegend) for 1 h at room temperature. Streptavidin-RBD complexes were separated by size-exclusion chromatography using a Superdex 200 Increase 10/300 GL column (Cytiva) equilibrated in 20 mM sodium phosphate (pH 7.2) and 200 mM NaCl buffer.

Biotinylated S1 protein was obtained commercially (BioLegend). A 4× molar excess was incubated with streptavidin-Alexa Fluor 647 (0.5 mg ml^−1^; BioLegend) for 30 min at room temperature directly before use. Practical guidelines on how antigen concentration can affect affinity selection can be found in Supplementary Fig. 9.

### Synthesis of BG-agarose

BG-agarose was synthesized by dissolving 500 mg of low-melting-point agarose (Sigma-Aldrich, A5030), 46 mg of 1,1ʹ-carbonyldiimidazole (Sigma-Aldrich) and 236 μl of triethylamine (Sigma-Aldrich) in 50 ml of DMSO (Sigma-Aldrich) and stirring the solution at room temperature under an argon atmosphere (adapted from ref. ^85^). After 1 h, 250 mg of O-(4-(aminomethyl)benzyl)guanine (Generon) was added, and the solution was stirred overnight under argon. The solution was diluted with 450 ml of warm distilled water and dialyzed against water using snakeskin dialysis tubing (3,500 Da molecular weight cut-off; Thermo Fisher Scientific). After six water exchanges, the product was lyophilized yielding a white, spongy material.

### Determination of agarose functionalization efficiency

BG-agarose (1.5%, wt/vol) was dissolved in PBS, melted by heating to 85 °C using a heated shaker and then cooled to 37 °C. BG-agarose beads (25 µm diameter) were generated by microfluidics (flow rates: aqueous phase, 6–7 μl min^−1^ and oil phase, 16–18 μl min^−1^). The emulsion was collected on ice to solidify the agarose. BG-agarose beads were demulsified by the addition of 800 μl PBS and 200 μl 1H,1H,2H,2H-perfluoro-1-octanol (PFO; Alfa Aesar), filtered through a 50 μm strainer and washed once in 15 ml FACS buffer (1% ultra-low IgG FBS (Thermo Fisher Scientific) in PBS). Beads (200,000 per condition) were incubated with serial 1:2 dilutions of GFP-SNAP overnight at room temperature, starting from 8.55 × 10^15^ GFP molecules per condition. Beads were washed twice in FACS buffer, and the GFP fluorescence was analyzed on an Attune Nxt flow cytometer (Thermo Fisher Scientific). Single beads were gated on side scatter versus forward scatter (log scale) and forward scatter height versus forward scatter (log scale). The mean GFP fluorescence and s.d. for each condition were determined in FlowJo (version 10.7; BD Biosciences).

### Antibody capture on agarose beads

BG-agarose beads (25 μM diameter, 1.5% (wt/vol) agarose) were generated by microfluidics as described previously and functionalized with 50 µM anti-mouse κ VHH for 1 h or anti-human κ and λ VHH for 15 min at room temperature. For the mouse antibodies, after washing twice in FACS buffer, an anti-streptavidin antibody (mouse IgG2bκ, clone 3A20.2; BioLegend) was added to 10^6^ beads at a concentration of 0.25 mg ml^−1^ (1:2 dilution) and incubated for 1 h at room temperature. GFP-biotin was tetramerized by incubation with APC-streptavidin (BioLegend) for 1 h at 10× molar excess. The tetramer (428 kDa) was purified from excess GFP-biotin (25 kDa) with a 100 kDa cut-off Amicon Ultra centrifugal filter (Merck). Functionalized agarose beads were washed twice and then incubated with 20 nM of the GFP–streptavidin tetramers and anti-mouse IgG2a/b BV421 antibody (1:10 dilution; clone R2-40, BD Bioscience) for 30 min on ice. After two washing steps in FACS buffer, the beads were analyzed on an Attune Nxt flow cytometer (Thermo Fisher Scientific). For the human antibodies, the beads were washed once in FACS buffer, and 400,000 beads were each incubated with 6.63 nM of either hRBD2, hRBD8 or hRBD10 for 20 min at 37 °C. Beads were stained with strep-RBD Alexa Fluor 647 (1 nM final concentration) for 30 min on ice. After washing once in FACS buffer, the beads were analyzed on a BD LSRFortessa.

### Biolayer interferometry competition assay

Biotinylated anti-mouse IgG1 antibody (10 µg ml^−1^; clone RMG1-1, BioLegend) was immobilized on streptavidin (SA) biosensors (Sartorius) for 300 s. Subsequently, 100 nM of mRBD1 antibody (IgG1κ) was captured for 600 s and then probed with either anti-mouse κ VHH–SNAP (100 nM, 300 s) followed by RBD (100 nM, 300 s), or vice versa. Human mAbs (100 nM of hRBD1 (IgG1κ) and hRBD3 (IgG1λ)) were directly immobilized on anti-human IgG Fc-capture (AHC) biosensors (Sartorius) for 600 s and then probed with either anti-human κ or λ VHH (100 nM, 300 s) followed by RBD (100 nM, 300 s), or vice versa. For controls, sensors were incubated in buffer instead of anti-mouse IgG1 (−anti-mouse IgG), buffer instead of recombinant mAb (−mAb), buffer instead of mAb/VHH/RBD (buffer control mouse sample) or buffer instead of VHH/RBD (buffer control human samples). All dilutions were performed in kinetics buffer (0.1% BSA and 0.02% Tween in PBS).

### Mouse experiments

C57BL/6 mice (8–14 weeks old) were immunized with EndoFit OVA (Invivogen) or SARS-CoV-2 RBD. For viability and secretion time course experiments, 33–38-week-old nonimmunized mice were used. Antigens were prepared as 2 mg ml^−1^ sterile-filtered solutions in PBS, mixed with an equal volume of Alhydrogel adjuvant (Invivogen) and incubated on a tube roller for 30 min at 4 °C. Each mouse was injected intraperitoneally with 100 μl of 1 mg ml^−1^ antigen. For confocal experiments and RBD antibody generation, mice were additionally injected intraperitoneally with 100 μl of 0.5 mg ml^−1^ OVA or RBD in PBS 21 days after the first immunization. Spleens were collected 10 days after the first immunization, and bone marrow cells were collected 21 days after the first or second immunization.

### Extraction of splenocytes and bone marrow

A single-cell suspension of splenocytes was prepared by mashing the spleens through a 100 μm cell strainer into wash buffer (PBS, 0.1% FBS and 2 mM EDTA). Splenocytes were washed in ice-cold wash buffer and then filtered again through a 70 μm strainer. For bone marrow extraction, the femur and tibias were removed from both legs, the ends of the bones were cut open with scissors and the bone marrow was flushed out using a 23-gauge needle and a syringe filled with wash buffer. A single-cell suspension was prepared by filtering through a 100 μm cell strainer. Red blood cells of both spleen and bone marrow were lysed by incubation in 1 ml of RBC lysis buffer (155 mM NH_4_Cl, 12 mM NaHCO_3_ and 0.1 mM EDTA) for 5 min at room temperature. Cells were washed once in wash buffer and used for ASC enrichment. All centrifugation steps were carried out at 300*g* for 5 min at 4 °C.

### Mouse ASC enrichment

Plasma cells were enriched using direct magnetic labeling with anti-CD138 (syndecan-1) microbeads (Miltenyi Biotec). Enriched cells were washed once in wash buffer, and an Fc blocking step was performed using the TruStain FcX (anti-mouse CD16/32) antibody (BioLegend).

### Mouse ASC encapsulation

BG-agarose (3–6%, wt/vol) was dissolved in PBS, melted by heating to 85 °C using a heated shaker and then cooled to 37 °C. For FACS experiments following OVA immunization, 6% BG-agarose was mixed with anti-mouse κ VHH–SNAP before encapsulation to obtain 100 μM VHH–SNAP and 3% BG-agarose. BG-agarose can be functionalized with VHH–SNAP before or after encapsulation, but in our hands, VHH addition after hydrogel bead formation and demulsification leads to a slightly better signal-to-noise ratio. For all other experiments, a 3% (wt/vol) BG-agarose solution was used directly without prior VHH–SNAP addition. ASCs obtained through magnetic selection (on average >2 × 10^6^ cells per spleen and >1.5 × 10^6^ cells for bone marrow) were washed, resuspended in encapsulation buffer (PBS, 10% ultra-low IgG FBS, 18% OptiPrep, 2 mM EDTA and 0.1% Pluronic F-68) at a concentration of 3 × 10^7^ cells per ml and filtered through a 40 μM strainer. Cells were then mixed with an equal volume of 3% BG-agarose in PBS. The solution was aspirated into microfluidic tubing and encapsulated with flow rates of 6–7 μl min^−1^ for the aqueous phase (cells in agarose) and 16–18 μl min^−1^ for the oil phase (mean number of cells per droplet *λ* = 0.12). During encapsulation, the microfluidic chip was heated to 37 °C using a heated glass microscope plate (Bioscience Tools), although this is not strictly necessary as the low-melting-point agarose will remain liquid for at least 1 h. We routinely ran two microfluidic chips in parallel, enabling encapsulation of up to 1.26 × 10^7^ cells per hour. Alternatively, the mean number of cells per droplet can be increased up to 0.3. The emulsion was collected on ice to solidify the agarose. Hydrogel beads were demulsified by the addition of 800 μl PBS and 200 μl PFO, filtered through a 50 μm strainer and washed once in 15 ml cold FACS buffer. If VHH–SNAP had not been added before encapsulation, the hydrogel beads were resuspended in 50 µM anti-mouse κ VHH–SNAP in PBS, incubated for 15 min at room temperature and then washed once in FACS buffer. For IgG secretion control experiments (designated as −VHH) and viability time course experiments, no VHH–SNAP was added. Hydrogel beads were then resuspended in prewarmed medium at a cell concentration of ≤10^6^ cells per ml (RPMI supplemented with 10% ultra-low IgG FBS, 20 mM HEPES, 50 μM ß-mercaptoethanol, 1 mM sodium pyruvate, 2 mM l-glutamine and 100 U penicillin/streptomycin) and incubated at 37 °C in 5% CO_2_ for the indicated time frames (1 h and 45 min for antibody selections and confocal experiments). For secretion and viability time course experiments, a fraction of the cells was not encapsulated but kept on ice during the encapsulation/functionalization process and was then incubated and stained in the same way as encapsulated cells. The medium supernatant of samples from the secretion time course was collected for IgG detection by ELISA. All centrifugation steps were carried out at 300*g* for 10 min at 4 °C.

To reduce potential ‘false positive’ events because of cross-contamination between beads, the sample can be incubated in a larger volume and slowly agitated/rotated during the secretion process. Sample agitation distributes contaminating antibodies across the whole bead population, of which 90% are empty beads and will be gated out during the sort.

### Staining of encapsulated mouse ASCs for flow cytometry

After incubation, samples were washed once in FACS buffer. For viability time course experiments, hydrogel beads and non-encapsulated ASCs were first stained with Calcein AM (Invitrogen) according to the manufacturer’s instructions. Samples were incubated with anti-CD138 BV785, anti-IgG1 PerCP-Cy5.5 and anti-CD45R/B220 AF647 for 30 min on ice and then washed once in FACS buffer. Before the acquisition, DAPI (2.5 µg ml^−1^; Sigma-Aldrich) was added to each tube. For secretion time course experiments, hydrogel beads and non-encapsulated ASCs were stained with anti-CD138 phycoerythrin (PE), anti-IgG1 BV421, anti-IgM BV605 and anti-CD45R/B220 BV785 for 30 min on ice and then washed once in FACS buffer. Before the acquisition, helix green (2.5 µM; BioLegend) was added to each tube.

For OVA antibody selection experiments, hydrogel beads were stained with anti-CD138 PE, anti-FLAG PE-Cy7, OVA-Alexa Fluor 647 (3 μg ml^−1^; Thermo Fisher Scientific), anti-IgG1 BV421, anti-IgG2a/2b BV421, anti-IgG2a BV421, anti-IgG3 BV421, anti-IgM BV605 and anti-CD45R/B220 BV785 for 30 min on ice and then washed once in FACS buffer. Before the acquisition, 5 μl of 7-aminoactinomycin D (7-AAD, 50 µg ml^−1^; BioLegend) or helix green (1 µM) were added to each tube. For RBD experiments, hydrogel beads were stained with strep-RBD Alexa Fluor 647 (40 nM), anti-IgG1 BV421, anti-IgG2a/2b BV421, anti-IgG2a BV421, anti-IgG3 BV421, anti-IgM BV605 and anti-CD138 BV785 for 30 min on ice and then washed once in FACS buffer. Before the acquisition, helix green (1 µM) was added to each tube. For antibody clones and dilutions, see Supplementary Table 6.

### Analysis of encapsulated mouse ASCs by confocal microscopy

Hydrogel beads were stained with OVA-Alexa Fluor 555 (1.5 μg ml^−1^; Thermo Fisher Scientific), anti-CD138 Alexa Flour 647 (1:80 dilution; BioLegend, clone 281-2), donkey anti-mouse IgG Alexa Fluor Plus 405 (Thermo Fisher Scientific, A48257; 1:200 dilution) for 30 min on ice and then washed once in FACS buffer. For confocal imaging, 5 µl of unfixed, unpermeabilized encapsulated cells were pipetted into microscopy-compatible 35 mm dishes (ibidi, 81156) and overlayed with 500 µl anti-evaporation oil (ibidi, 50051). Imaging was performed on a Leica TCS SP8 confocal microscope. Fluorophores were excited with a 405 nm, a 552 nm and a 638 nm laser, respectively. Images were analyzed with Fiji^86^. The plot profile function was used to determine the signal distribution across the hydrogel beads.

### Analysis and sorting of mouse ASCs by flow cytometry

For viability and secretion time course experiments, samples were analyzed on a BD LSRFortessa. Before sorting, hydrogel beads were filtered through a 50 μm strainer. Hydrogel beads were sorted on a four-laser BD FACSAria III using a 100 μm nozzle into 96-well plates (Frame Star, 4titude) that contained 4 μl of lysis buffer (3 U μl^−1^ RNAseOUT RNase inhibitor and 1 mM DTT in 0.5× PBS^87^). Plates were spun down for 30 s at 400*g* immediately after the sort, placed on dry ice and then stored at −80 °C until processing. Hydrogel beads were gated to contain live ASCs (CD138^+^) that were IgG-positive but IgM-negative. The antigen signal (OVA or RBD) of hydrogel beads that were IgG^−^/IgM^−^ was used as guidance for setting the antigen-positive gate. For OVA experiments, an anti-FLAG antibody was used to identify cells encapsulated into the functionalized BG-agarose hydrogel as the VHH–SNAP construct contained a FLAG tag. The secretion control sample, to which no VHH–SNAP–FLAG was added, was therefore gated as FLAG^−^.

### PBMC isolation from peripheral blood

Peripheral blood (40 ml) was collected from two study participants in lithium heparin tubes. PBMCs were isolated by density gradient centrifugation using Histopaque-1077 (Sigma-Aldrich). Blood was diluted 1:1 with PBS (room temperature), and 35 ml of blood was layered onto 15 ml of Histopaque and centrifuged (acceleration: 1 and deceleration: 0, 800*g* for 20 min at room temperature). The buffy coat was removed, and the PBMCs were washed twice in ice-cold wash buffer (0.1% FBS and 2 mM EDTA in PBS). PBMCs were counted, and one half was used directly for encapsulation experiments while the other half was resuspended in FBS and then mixed 1:1 with freezing medium (50% FBS and 10% DMSO in RPMI). PBMCs were put into a freezing container (cooling rate: −1 °C min^−1^) and stored at −80 °C before transfer to liquid nitrogen. Unless stated otherwise, all centrifugation steps were carried out at 400*g* for 10 min at 4 °C.

### PBMC stimulation

Normal cryopreserved human PBMCs (10 million cells per vial; Lonza, 4W-270) were thawed quickly at 37 °C and added dropwise to 10 ml of prewarmed PBMC medium (RPMI 1640 supplemented with 10% FBS, 2 mM GlutaMAX, 1 mM sodium pyruvate, 10 mM HEPES (pH 7.4), 1× MEM nonessential amino acids (Sigma-Aldrich), 50 μM ß-mercaptoethanol, 0.25 μg ml^−1^ amphotericin B and 100 U ml^−1^ penicillin/streptomycin) with 100 μg ml^−1^ DNase (STEMCELL Technologies). Cells were spun down at 500*g* for 10 min at 4 °C, resuspended in 2 ml of medium and rested for 1 h in the incubator (37 °C in 5% CO_2_). Stimulation medium (2× concentration) was prepared by adding 400 ng ml^−1^ HA-tagged rhCD40L (R&D Systems) and 100 ng ml^−1^ anti-HA tag antibody (R&D Systems, clone 543851) to PBMC medium and incubating for 15 min at room temperature to cross-link the proteins. Afterward, 100 ng ml^−1^ human interleukin (IL)-21 (Peprotech) and 20 μg ml^−1^ anti-IgM antibody (polyclonal F(ab′)_2_ fragment goat anti-human; Jackson ImmunoResearch, AB_2337553) were added. Cells were washed once in magnetic-activated cell sorting (MACS) buffer (2 mM EDTA, 0.5% BSA in PBS), and B cells were negatively selected with the Human Pan-B Cell Isolation Kit (Miltenyi Biotec). Enriched B cells were spun down and resuspended in PBMC medium supplemented with 80 μg ml^−1^ apotransferrin (Sigma-Aldrich) at 10^6^ cells per ml. Cells were then mixed 1:1 (vol/vol) with stimulation medium and incubated for 96 h.

### Encapsulation of stimulated PBMCs

A preheated 6% (wt/vol) BG-agarose solution in PBS was mixed 1:1 (vol/vol) with anti-human κ VHH–SNAP (final concentration 100 μM) and left at 37 °C for 15 min before use. Cells were washed once in MACS buffer, and CD38^+^ cells were positively selected using the CD38 MicroBead kit (Miltenyi Biotec). CD38^+^ cells were washed once, and a blocking step using FcR Blocking Reagent (Miltenyi Biotec) was performed. Cells were then resuspended in encapsulation buffer and filtered through a 40 μM strainer to obtain a concentration of 3 × 10^7^ cells per ml. Cells were then mixed with an equal volume of VHH-functionalized 3% BG-agarose in PBS, encapsulated into agarose and demulsified as previously described. Beads were filtered through a 50 μm strainer and washed once in 15 ml cold FACS buffer. Afterward, hydrogel beads were resuspended in prewarmed PBMC medium (supplemented with 10% ultra-low IgG FBS instead of normal FBS) and incubated for 1 h and 45 min at 37 °C in 5% CO_2_. All centrifugation steps were carried out at 300*g* for 10 min at 4 °C.

### Staining of encapsulated normal PBMCs for FACS/microscopy

After incubation, hydrogel beads were washed once in FACS buffer and then stained with anti-CD14 VioBlue, anti-CD3 VioBlue, anti-IgD VioBlue, anti-CD19 VioBright-FITC, anti-CD27 APC-Vio770, anti-CD20 PE-Vio770, anti-CD38 APC, anti-IgG Fc PE, anti-IgM BV711 and anti-FLAG Alexa Fluor 594. The sample was incubated on ice for 30 min and washed once in FACS buffer. DAPI (1:50,000) was added before analysis on an Attune NxT flow cytometer. Hydrogel beads were examined for APC (anti-CD38) and PE fluorescence (IgG secretion) under an EVOS FL fluorescence microscope (Thermo Fisher Scientific) using filters for Cy5 (ex, 628/40 nm and em, 692/40 nm) and RFP (ex, 531/40 nm and em, 593/40 nm), respectively. For antibody clones and dilutions, see Supplementary Table 6.

### Encapsulation of PBMCs post vaccination

Blood was collected from study participants 7–9 days after the second BNT162b2 vaccine dose. Freshly isolated PBMCs were used directly for B cell enrichment. Cryopreserved PBMCs were thawed quickly at 37 °C and added dropwise to 10 ml of prewarmed medium (RPMI 1640 supplemented with 10% ultra-low IgG FBS, 2 mM GlutaMAX, 1 mM sodium pyruvate, 10 mM HEPES (pH 7.4), and 100 U ml^−1^ penicillin/streptomycin) with 100 μg ml^−1^ DNase (STEMCELL Technologies). Cells were spun down (400*g* for 10 min at 4 °C) and washed once in MACS buffer. B cells were negatively enriched with the Human Pan-B Cell Isolation Kit (Miltenyi Biotec). On average, 5–8 × 10^6^ cells were obtained per purification. Enriched B cells were spun down, and a blocking step using FcR Blocking Reagent (Miltenyi Biotec) was performed. Cells were resuspended in encapsulation buffer and filtered through a 40 μM strainer to obtain a concentration of 3 × 10^7^ cells per ml. Cells were mixed with an equal volume of 3% BG-agarose in PBS and encapsulated and demulsified as previously described for mouse ASC encapsulation (*λ* = 0.12; two microfluidic chips run in parallel). Hydrogel beads were filtered through a 50 μm strainer and washed once in 15 ml cold FACS buffer. Beads were resuspended in PBS with anti-human VHH-SNAPs (anti-κ and anti-λ; 50 µM of each) and incubated for 15 min at room temperature. Hydrogel beads were washed once in cold FACS buffer and resuspended in prewarmed medium supplemented with 10 ng ml^−1^ human IL-6 and incubated for 1 h and 45 min at 37 °C in 5% CO_2_.

### Staining and sorting of encapsulated PBMCs post vaccination

For sorting with RBD, hydrogel beads were washed once in FACS buffer and stained with strep-RBD Alexa Fluor 647 (40 nM), anti-IgG Fc PE, anti-IgM BV785, anti-CD38 BV421 and anti-CD20 BV510 for 30 min on ice. Beads were washed once in FACS buffer, and helix green (1 µM) was added. For sorting with RBD and S1, hydrogel beads were washed once in FACS buffer and then stained using strep-S1 Alexa Fluor 647 (40 nM), strep-RBD Alexa Fluor 488 (40 nM), anti-IgG Fc PE, anti-IgA PE, anti-IgM BV785, anti-CD38 BV510 and anti-CD20 PE-Cy7 for 30 min on ice. Hydrogel beads were washed once in FACS buffer, and DAPI (Thermo Fisher Scientific; 1:50,000) was added to the sample. Beads were (index) sorted on a four-laser BD FACSAria III using a 100 μm nozzle into 96-well plates (Frame Star, 4titude) that contained 6 μl of lysis buffer (5% PEG 8000, 0.1% Triton X-100, 0.5 U μl^−1^ RNAseOUT, 0.5 μM OligoT30VN, 0.5 μM random hexamers and 0.5 mM/each dNTP). The antigen signal of hydrogel beads that showed neither the IgG/IgA nor the IgM signal was used as guidance for setting the antigen-positive gate. Plates were spun down for 30 s at 400*g* immediately after the sorting, placed on dry ice and then stored at −80 °C until processing. For antibody clones and dilutions, see Supplementary Table 6.

### Reverse transcription, PCR and sequencing (mouse)

Plates were thawed on ice for 5 min and spun down at 400*g* for 1 min at 4 °C. For lysis and reverse transcription, a combination of previously published protocols^87,88^ was used together with the template-switch oligo from the 10X Chromium Next GEM Single Cell V(D)J Reagent Kit v1.1 (without barcode and unique molecular identifier). See Supplementary Table 7 for primer sequences. Per well, 7 μl of lysis and annealing mix (0.6% IGEPAL, 5.5 mM random hexamer primers, 3.99 mM anchored oligo-dT and 0.9 U μl^−1^ RNaseOUT) were added, and the plate was incubated at 65 °C for 5 min. Plates were placed on ice for 5 min, and 10 μl of reverse transcription mix was added to each well (15% PEG 8000, 2× Maxima RT buffer, 2 mM dNTPs, 4 μM template-switch oligo (10X_TSO) and 4 U μl^−1^ Maxima H Minus Reverse Transcriptase). Plates were spun down and transferred to a thermocycler for reverse transcription (90 min at 42 °C, five cycles of 2 min at 50 °C followed by 2 min at 42 °C). Plates containing cDNA were stored at −20 °C. Leader peptide and variable regions of both the heavy chain and κ light chain were then amplified in two rounds of semi-nested PCR. For the first PCR, 8 μl of cDNA reaction mix was added to 17 μl of PCR mix using KAPA HiFi HotStart polymerase and KAPA HiFi Fidelity Buffer according to the manufacturer’s instructions (primer concentrations: 0.3 μM fw_TSO_PCR1, 0.3 μM mIgG12abc, 0.1 μM mIgG2b, 0.1 μM mIgG3-1, 0.1 μM mIgG3-2 and 0.2 μM mκ). The PCR was performed at 95 °C for 3 min, followed by 20 cycles of 20 s at 98 °C, 30 s at 67 °C and 70 s at 72 °C and a 5 min final elongation at 72 °C. From the first PCR reaction, 5 μl was transferred to the second PCR (final volume, 25 μl) for 22 amplification cycles. DNA obtained from anti-OVA hits was cut out of agarose gels, and amplicons from heavy and light chains were purified separately by gel extraction (Zymoclean Gel DNA Recovery Kit, Zymo Research).

For RBD experiments, PCRs were split after the first PCR, and heavy and light chains were amplified separately using final primer concentrations of 0.3 μM for the reverse κ primer and the same conditions for the heavy-chain primers as for the first PCR. Amplicons from anti-RBD hits were purified by SpeedBead (Sigma-Aldrich) purification. PCR reactions that resulted in sufficient material (>100 ng determined by absorbance measurement at 260 nm) were subjected to Sanger sequencing. We found that sequencing efficiency was suboptimal with this protocol and recommend following a published protocol that does not use a template-switch oligo^87^. We also advise decreasing the amount of PFO for demulsification or using chemical-free breaking of the droplet emulsion, as PFO can impact the efficiency of enzymatic reactions^89^.

### Reverse transcription, PCR and sequencing (human)

Reverse transcription in each well was performed based on a modified Smart-seq3 protocol using a template-switch approach^90^. Specifically, all volumes for the lysis and reverse transcription steps were doubled, and random hexamers (0.5 μM final concentration) were added to the lysis buffer mix. All other steps were performed according to the published protocol. All oligonucleotides are listed in Supplementary Table 8. For anti-RBD antibodies, 12 μl of PCR 1 mix (final concentrations in KAPA HiFi Fidelity Buffer: 0.3 mM/each dNTPs, 0.5 mM MgCl_2_, 0.5 μM fw_Read1/2 primer and 0.1 μM of each reverse primer (rv_IgG_HC_PCR1, rv_kappa_PCR1, rv_lambda_PCR1), 0.02 U μl^−1^ KAPA HiFi HotStart polymerase) were added to each well after reverse transcription. PCR reactions were performed at 95 °C for 3 min, 20 cycles of 20 s at 98 °C, 30 s at 65 °C and 2 min 30 s at 72 °C and a 5 min final elongation step at 72 °C. For the semi-nested second PCR, 5 μl were transferred from the first PCR, and PCRs were performed using KAPA HiFi HotStart polymerase and KAPA HiFi Fidelity Buffer. Because we initially planned to use Illumina sequencing^76^, we performed two sets of second PCRs, one that introduces the full-length Illumina Read 1 or Read 2 at the 5′ end and Read 2 or Read 1 at the 3′ end. For this reaction, fw_R1_PCR2 was combined with rv_R2 primers, and fw_R2_PCR2 was combined with rv_R1 primers at 0.3 μM final concentration each in 25 µl. PCR reactions were performed as previously but with 22 amplification cycles. PCR products were purified using SpeedBeads (Sigma-Aldrich) and eluted with 15 μl of nuclease-free water. For the addition of barcode indices and Illumina P5/P7 adapters, a third PCR was performed. For PCRs with 5′ Read 1, i5 primers (P5_1–P5_96, used as well barcodes) were used as forward primers, and i7 primers (P7_1–P7_20, used as plate barcodes) were used as reverse primers. For PCRs with 5′ Read 2, i5 primers were used as reverse primers and i7 primers as forward primers. PCRs were performed using KAPA HiFi HotStart polymerase and KAPA HiFi Fidelity Buffer in a 25 μl volume, with 0.3 μM of primers and 2 μl of PCR 2 as template with annealing at 65 °C and 22 cycles of amplification. PCR products were purified using SpeedBeads (Sigma-Aldrich). PCR reactions that resulted in sufficient material (>100 ng determined by absorbance measurement at 260 nm) were subjected to Sanger sequencing. We found that this protocol did not yield high sequencing efficiencies (≅17%) and therefore decided to amplify the cDNA from RBD/S1 hits with a different primer set that does not rely on template switching^91^. After reverse transcription, the cDNA was diluted by the addition of 27.4 µl of nuclease-free water. For the first PCR reaction, 4 µl of diluted cDNA were mixed with 21 µl of PCR 1 mix (final concentrations in KAPA HiFi Fidelity Buffer: 0.3 mM/each dNTP, 0.5 mM MgCl_2_, 0.2 µM oPR_IGHV mix, 0.2 µM oPR_IGKV mix, 0.2 µM oPR_IGLV mix, 0.2 µM oPR_1st_rv mix and 0.02 U μl^−1^ KAPA HiFi HotStart polymerase). PCR reactions were performed at 95 °C for 3 min, followed by 50 cycles of 30 s at 98 °C, 30 s at 62 °C and 45 s at 72 °C and a 5 min final elongation step at 72 °C. Three separate, semi-nested second PCRs were performed for the amplification of heavy chains, κ and λ light chains. For the second PCR, 4 µl of the first PCR was used directly and mixed with 21 µl of PCR 2 mix (final concentration in KAPA HiFi Fidelity Buffer: 0.3 mM/each dNTP, 0.3 µM forward primer mix (oPR_IGHV mix, oPR_IGKV mix or oPR_IGLV mix) with the respective 0.3 µM reverse primer mix (IgG/IgA rv mix, 3′ Cκ 494 (κ) or 3′ XhoI Cλ (λ)), 0.02 U μl^−1^ KAPA HiFi HotStart polymerase) with the same conditions as PCR 1. PCR products were purified using SpeedBeads (Sigma-Aldrich) and sequenced by Sanger sequencing, leading to an increase in matched heavy- and light-chain sequences (over 40%).

### Bioinformatic analysis

Antibody sequences were annotated using international ImMunoGeneTics information system (IMGT)/V-Quest from the IMGT database^92^. All clonotyping was performed using an in-house script (code available in the Supplementary Information), with antibodies greedily clustered if they had a common IGHV gene assignment, the same length CDR3 and within 80% CDR3 amino acid identity of the cluster representative. The CoV-AbDab^35^ (February 2022 version) was downloaded as a set of anti-coronavirus reference antibodies with functional annotations. Clonotyping was then applied to functionally cluster input files containing each set of ASC-derived antibodies concatenated with all CoV-AbDab antibodies with a complete IGHV and CDRH3 label. An alternative clustering method based on grouping antibodies by similar predicted 3D structure (SPACE) was also applied^51^, using a threshold structural similarity of 0.75 Å. This was performed over each set of ASC-derived antibodies coupled with the subset of antibodies in CoV-AbDab with full variable heavy (VH) and variable light (VL) chain sequences (as sequence coverage over all six CDRs is required by SPACE).

### Cloning of antibodies

For mouse antibodies, primers were designed to amplify the variable regions, including the leader peptides. To generate PCR amplicons, Q5 Polymerase High-Fidelity 2X Master Mix (New England Biolabs (NEB)) was used according to the manufacturer’s protocol using the second sequencing PCR as a template. For anti-OVA antibodies, variable regions and leader peptides were cloned into pVITRO1 (Invivogen) plasmids that contained the constant regions for mouse IgG1 κ heavy and light chains using Gibson assembly^83^. For anti-RBD antibodies, standard restriction cloning using the type IIS restriction enzyme BsmBI-v2 (NEB) was used. Antibodies were excluded from further testing if the plasmid sequencing did not match the expected sequence.

For human antibodies, primers were designed to amplify the variable regions, excluding the leader peptides. To generate PCR amplicons, Q5 Polymerase High-Fidelity 2X Master Mix (NEB) was used with the second or third PCR as a template. Variable regions were cloned into pVITRO1 plasmids that already contained the constant regions for human IgG1 heavy and light chains and leader peptides (κ or λ; Addgene, plasmids 61883 and 50366)^93^ using Gibson assembly^83^. For amino acid sequences of the variable regions of the antibodies, see Supplementary Tables 2 and 4.

### Antibody expression and purification

FreeStyle 293-F cells (Thermo Fisher Scientific) were maintained in shaking flasks in Freestyle 293 Expression Medium at 0.4–2 × 10^6^ cells per ml (incubator conditions: 37 °C, 70% humidity, 8% CO_2_ and 125 rpm). Per 10^6^ cells, 1.2 μg of DNA was mixed with 2.4 μl of 1 mg ml^−1^ polyethylenimine (linear PEI, MW 25,000, Polysciences) in one-tenth of the final culture volume of medium. The mixture was vortexed for 15 s followed by 15 min incubation at room temperature and added to a culture of 10^6^ cells per ml. Valproic acid (2-propyl-pentanoic acid; Sigma-Aldrich) was added 4 h post transfection to a final concentration of 3.5 mM. The culture supernatant was collected 1 week after transfection by centrifugation for 15 min at 4,000*g*, sterile filtered and stored at 4 °C. The supernatant was added to a gravity column containing 0.5 ml of Pierce Protein G Agarose (Thermo Fisher Scientific) pre-equilibrated with PBS. The column was washed with ten CVs of PBS, and the antibodies were eluted in several fractions of 250 μl with elution buffer (10 mM glycine, pH 2.7) into collection tubes containing 12.5 μl of neutralization buffer (1 M Tris–HCl, pH 9.0). The fractions containing protein (measured by absorbance at 280 nm) were pooled, and the buffer was exchanged to PBS. The purity of the proteins was confirmed by SDS–PAGE. Antibodies were excluded from further testing if it was not possible to express the antibody in a single attempt.

### ELISA

#### Anti-mouse IgG ELISA

The concentration of mouse IgG in the supernatant of encapsulated and non-encapsulated samples was determined with the IgG (total) Mouse Uncoated ELISA Kit (Invitrogen, 88-50400; assay range 1.56–100 ng ml^−1^). The assay was performed with 50 µl of undiluted supernatant, a standard concentration range of 0.78–100 ng ml^−1^ IgG, and with medium used as a negative control. Absorbance was read at 450 nm in duplicate in a Varioskan LUX plate reader (Thermo Fisher Scientific). The standard curve was created in GraphPad Prism using the Sigmoidal, 4PL, X is log(concentration) function. The mean signal of negative control wells was subtracted from all measurements.

#### Anti-RBD/S1 ELISA

Microtiter plates (384 well Nunc MaxiSorp, flat bottom; Thermo Fisher Scientific) were coated with 20 μl of 2 μg ml^−1^ RBD or S1 in PBS overnight at 4 °C. Plates were washed twice with 90 μl of PBS-T and incubated for 1 h at room temperature with 40 μl of blocking buffer (2.5% BSA in PBS-T). Plates were washed twice with PBS-T. Antibody samples were diluted in 1% BSA in PBS-T (final concentrations: 400 nM, 200 nM, 20 nM, 2 nM, 0.2 nM, 0.02 nM, 0.002 nM and 0.0002 nM), and 20 μl of the sample was added per well followed by incubation at room temperature for 1 h. Commercial anti-RBD antibodies (mouse IgG1κ, clone 1035753 (R&D Systems) or human IgG1κ, clone AM001414 (BioLegend)) and isotype controls (mouse IgG1κ, clone MG1-45 (BioLegend)) or human IgG1κ, clone QA16A12 (BioLegend)) were used as controls. Plates were incubated for 1 h at room temperature, washed four times with PBS-T and 20 μl of polyclonal HRP-conjugated detection antibodies (goat anti-mouse (Thermo Fisher, G-21040) at 1:1,000 dilution or goat anti-human (Abcam, ab7153) at 1:10,000 dilution) were added to each well and incubated for 30 min (anti-mouse) or 45 min (anti-human) at room temperature. The plate was washed three times with PBS-T and one time with PBS. 3,3′,5,5′-tetramethylbenzidine (Thermo Fisher Scientific; 20 μl per well) was added followed by the addition of 0.16 M H_2_SO_4_ (20 μl per well) after 5 min. Absorbance was read at 450 nm and 540 nm in a Tecan Infinite 200 PRO plate reader. The absorbance at 540 nm was subtracted from the signal at 450 nm. To obtain EC_50_ values, titration curves were plotted as absorbance versus log (antibody concentration) and then analyzed by nonlinear regression using the Sigmoidal, 5PL, X is log(concentration) function in GraphPad Prism^94^. For the purpose of visualization, mouse antibodies with no quantifiable ELISA signal at the tested concentrations were assigned an arbitrary EC_50_ of 1,000 nM. Mouse antibodies that did not show binding to RBD under these conditions were also tested in an anti-streptavidin ELISA (under the same conditions as described previously except that plates were coated with 20 μl of 2 μg ml^−1^ streptavidin). Under the tested conditions, no binding to streptavidin was observed.

### Determination of VHH and antibody binding affinities

Binding affinities were determined using the Octet RED96 BLI system. VHH binding affinities were measured by immobilizing recombinant mouse and human isotype control antibodies (10 μg ml^−1^) of human IgG1λ (clone AbD00264_hIgG1, BioRad) or mouse IgG1κ (clone P3.6.2.8.1, Thermo Fisher Scientific) on preconditioned anti-human or anti-mouse IgG Fc-capture biosensors (AMC and AHC; Sartorius) for 600 s. Control sensors were incubated in kinetics buffer (0.1% BSA and 0.02% Tween in PBS) during the antibody loading step. Biosensors were then probed with different concentrations of VHH. Mouse mAbs (7.5 μg ml^−1^ anti-OVA and 5 μg ml^−1^ anti-RBD) were captured on preconditioned anti-mouse IgG Fc-capture biosensors (AMC; Sartorius) for 600 s. Human mAbs (5 μg ml^−1^ or 7.5 µg ml^−1^) were captured on preconditioned anti-human IgG Fc-capture biosensors (AHC; Sartorius) for 600 s. Control sensors were incubated in kinetics buffer during the antibody loading step. Biosensors were then probed with different concentrations of the corresponding antigens. All dilutions were performed in kinetics buffer. Sensors were regenerated with 10 mM glycine (pH 1.7). Data were analyzed using Octet data analysis software using a 1:1 binding model. For the purpose of visualization, one human antibody with no quantifiable *K*_*D*_ at the tested concentrations was assigned an arbitrary *K*_*D*_ value of 10^−7^ M. For individual binding curves of human RBD and S1 binders, see Supplementary Fig. 2.

### Live virus neutralization assays

The SARS-CoV-2 viruses used in this study were a WT (lineage B) isolate (SARS-CoV-2/human/Liverpool/REMRQ0001/2020), a kind gift from I. Goodfellow (University of Cambridge), isolated by L. Turtle (University of Liverpool), D. Matthews and A. Davidson (University of Bristol)^95,96^, and an Omicron (lineage B.1.1.529) variant, a kind gift from R. Gupta (University of Cambridge)^97^. Half-maximal inhibitory concentrations (IC_50_) for indicated mAbs were measured essentially as previously described^37–39^. In brief, luminescent reporter cells expressing SARS-CoV-2 papain-like protease-activatable circularly permuted firefly luciferase (FFluc) were seeded in flat-bottomed 96-well plates. HEK293T-ACE2-30F-PLP2 cells (clone B7; available from the National Institute for Biological Standards and Control, 101062) were used to test mouse antibodies^37^, and A549-ACE2-TMPRSS2-30F-PLP2 cells (clone E8) were used to test human antibodies^97^. The next day, SARS-CoV-2 viral stock (multiplicity of infection (MOI) = 0.01) was pre-incubated with a threefold dilution series of each antibody for 1 h at 37 °C, then added to the cells. Twenty-four hours post infection, cells were lysed in Bright-Glo Luciferase Buffer (Promega) diluted 1:1 with 1% NP-40 in PBS, and FFluc activity was measured by luminometry. Experiments were conducted in duplicate or triplicate, as indicated. To obtain IC_50_ values, titration curves were plotted as FFluc versus log (antibody concentration) and then analyzed by nonlinear regression using the Sigmoidal, 4PL, X is log(concentration) function in GraphPad Prism. IC_50_ values were quantified when (1) at least 50% inhibition was observed at the highest antibody concentration tested (100 μg ml^−1^), and (2) a sigmoidal curve with a good fit was generated. For purposes of visualization and ranking, antibodies with no quantifiable neutralizing capacity were assigned an arbitrary IC_50_ of 1,000 μg ml^−1^. For individual neutralization curves, see Supplementary Fig. 3.

### Epitope binning of mAbs

For in-tandem assays of mouse antibodies, biotinylated RBD (10 μg ml^−1^) was immobilized on streptavidin BLI biosensors (Sartorius) for 300 s. The biosensors were then probed with 100 nM of the first antibody for 600 s followed by 100 nM of the second antibody for 300 s. Independent binding of both clones was verified with a buffer control. For classical sandwich assays of human antibodies, 10 µg ml^−1^ of the first antibody was immobilized on IgG Fc-capture biosensors for 600 s. After blocking of free capture sites on the biosensors with isotype control (10 µg ml^−1^; human IgG1κ, clone QA16A12; BioLegend) for 600 s, sensors were incubated with 20 nM RBD for 300 s. Biosensors were then probed with the second antibody (10 µg ml^−1^) for 300 s. All dilutions were performed in kinetics buffer (0.1% BSA and 0.02% Tween in PBS). For individual binding curves, see Supplementary Fig. 4.

### Purification of mRBD2 Fab and RBD–Fab complex

DNA encoding for the heavy and light chains of the Fab region of mRBD2 was cloned into a bi-cistronic expression cassette in the modified pExp vector. The light chain was under the control of a T7 promoter followed by a short intergenic region containing a favorable ribosomal binding site and the heavy chain with an added C-terminal His-tag. The pExp-Fab expression vector was cotransformed with plasmid pRARE2 (extracted from Rosetta2 (DE3) cells; Novagen) into Shuffle T7 Express cells (NEB). Cells were grown in 2xYT media with 100 µg ml^−1^ ampicillin and 15 µg ml^−1^ chloramphenicol to OD_600_ 0.8–1.0, and protein production was induced with 0.4 mM IPTG and proceeded for 16 h at 20 °C. Cells were collected, resuspended in lysis buffer (20 mM HEPES–NaOH (pH 7.2), 300 mM NaCl, 20 mM imidazole, 250 µg ml^−1^ of DNase I and 1 mM PMSF) and then lysed by sonication. Lysate was cleared by centrifugation, and protein was purified by Ni-affinity chromatography using PureCube Ni-NTA agarose resin. After washing the column with 20 CV of 20 mM HEPES–NaOH (pH 7.2), 300 mM NaCl and 20 mM imidazole, the protein was eluted in 5 CV of 20 mM HEPES–NaOH (pH 7.2), 300 mM NaCl and 250 mM imidazole. Next, the buffer of the eluted protein was exchanged to 20 mM HEPES–NaOH (pH 7.2) and 20 mM NaCl using a HiPrep 26/10 Desalting column (Cytiva), and the Fab was loaded onto the HiTrap Capto SP ImpRes column (Cytiva). The protein was eluted in a linear gradient of 20–1,000 mM NaCl buffer containing 20 mM HEPES–NaOH (pH 7.2). Peak fractions were combined, and the protein was concentrated using 30 kDa MWCO Amicon Ultra concentrators and further purified by size-exclusion chromatography using the HiLoad Superdex 75 pg 16/60 column (Cytiva) equilibrated in 20 mM Tris–HCl (pH 7.2) and 250 mM NaCl. For the RBD–Fab complex formation, an excess of RBD was incubated with concentrated purified Fab for 1 h at 22 °C, and then the complexes were separated by size-exclusion chromatography using the HiLoad Superdex 75 pg 16/60 column (Cytiva) equilibrated in 20 mM Tris–HCl (pH 7.2) and 250 mM NaCl. The RBD–Fab protein complex in peak fractions was concentrated to 10 mg ml^−1^ using 30 kDa MWCO Amicon Ultra concentrators and used for crystallization trials.

### RBD-mRBD2 Fab crystallization and structure determination

Crystallization of the RBD–Fab complex was performed in sitting drops using the mosquito liquid dispensing robotic system (SPT Labtech) by mixing 200 nl of protein solution with 200 or 100 nl of precipitant in 96-well 2-drop MRC crystallization plates. Crystals appeared in several conditions of the Ligand-Friendly Screen (Molecular Dimensions) after 3–8 days at 16 °C; they were cryo-cooled in liquid nitrogen without the need for additional cryoprotectant. The crystals resulting in the presented structural data grew in a condition containing 100 mM Bis-Tris propane (pH 6.5), 200 mM potassium thiocyanate, 20% wt/vol PEG 3350 and 10% vol/vol ethylene glycol. X-ray diffraction data were collected at Diamond Light Source on beamline I04 and processed by auto-processing pipelines using autoPROC and Staraniso (Global Phasing). The structure was solved using molecular replacement (Phaser^98^) with structures of the RBD (Protein Data Bank (PDB): 6YM0) and a Fab fragment (PDB: 3BGF) as search models. The structure was refined with phenix.refine^99^ and autoBuster (Global Phasing) with manual fixing using Coot^100^. The final structure (PDB: 8BE1) contains two RBD:Fab complexes per asymmetric unit. As the complex made from chains D, E and F is likely affected by crystal packing (ca. 14° shift in the RBDs relative to the Fab; Supplementary Fig. 5), we used the other complex for analysis. Crystallographic data collection, data reduction and refinement statistics are shown in Supplementary Table 9.

### Data analysis

Flow cytometry data were analyzed using FlowJo (version 10.7). Analysis of Sanger sequencing reads was performed using Geneious Prime (version 2019.2.1; Biomatters). Antibody sequences (V gene, J gene, nt identity, CDR3) were analyzed with IMGT/V-Quest from the IMGT database^92^. All other bioinformatics were performed using Python. Clonotyping was performed using an in-house script (code available in Supplementary Information). Structural clustering was performed using the SPACE algorithm^51^ (codebase released with the original paper at 10.1371/journal.pcbi.1009675). All BLI data were analyzed using Octet data analysis software (version 11.0.0.4). Microscopy data were analyzed using Fiji^86^. X-ray diffraction data were processed using autoPROC (version 1.0.2) and Staraniso (version 1.0.2) (Global Phasing). XIA2 (version 3.8.dev0) and DIALS (version 3.dev.620-g8f4b6839d) were used for crystallographic diffraction data processing. Phenix (version 1.16), CCP4 package (version 7.1), Buster (version 2.10.4) and Coot (version 0.9.8.1) were used for structure determination. PyMOL (version 2.5.0) was used for structure analysis and to generate structure figures. Additional data were analyzed using GraphPad Prism 6 and Microsoft Excel. Adobe Illustrator (2015) was used to create all other figures and illustrations.

### Reporting summary

Further information on research design is available in the Nature Portfolio Reporting Summary linked to this article.

## Online content

Any methods, additional references, Nature Portfolio reporting summaries, source data, extended data, supplementary information, acknowledgements, peer review information; details of author contributions and competing interests; and statements of data and code availability are available at 10.1038/s41587-024-02346-5.

## Supplementary information

Supplementary Information Supplementary Figs. 1–9.

Reporting Summary

Supplementary Tables Supplementary Table 1 Characteristics of expressed mouse anti-RBD antibodies. Supplementary Table 2 Amino acid sequences of variable regions of expressed mouse antibodies. Supplementary Table 3 Characteristics of expressed human antibodies. Supplementary Table 4 Amino acid sequences of variable regions of expressed human antibodies. Supplementary Table 5 Amino acid sequences of expressed proteins (VHH–SNAP and RBD). Supplementary Table 6 Antibody clones and dilutions used in flow cytometry experiments. Supplementary Table 7 Oligonucleotide sequences for RT–PCR of antibody variable genes from mouse. Supplementary Table 8 Oligonucleotide sequences for RT–PCR of antibody variable genes from human. Supplementary Table 9 Crystallographic data collection and refinement statistics.

Supplementary Code Script used for clonotyping.

Supplementary Data Source data for Figs. 7 and 9.

## Source data

Source data for Figs. 2, 3 and 4 and Extended Data Figs. 1, 3, 6, 9 and 10. Statistical source data for Fig. 2c. Statistical source data for Fig. 3c. Statistical source data for Fig. 4d. Statistical source data for Extended Data Fig. 1b. Statistical source data for Extended Data Fig. 3a,b,d. Statistical source data for Extended Data Fig. 6c,d. Statistical source data for Extended Data Fig. 9c. Statistical source data for Extended Data Fig. 10a,b.

## Data Availability

All reference data used in clustering were sourced from publicly available studies via the CoV-AbDab (http://opig.stats.ox.ac.uk/webapps/covabdab/). Sequences of experimentally verified antigen-specific antibodies were deposited at Genbank (accessions OQ208846–OQ208931). Sequences of anti-SARS-CoV-2 antibodies were also deposited in the CoV-AbDab. Crystallographic data were accessed through RCSB PDB (accessions 6YM0, 3BGF and 6M0J). Crystallographic data were deposited at RCSB PDB under accession 8BE1. A file of the microfluidic chip design is available on DropBase (https://openwetware.org/wiki/DropBase:agarose_bead_generator). The plasmid for His-Zbasic-TEV-RBD-Avi is available on Addgene (195000). Flow cytometry raw data files and other plasmids generated in this study are available on request. All other data are available in the main text or in the Supplementary Information/source data. Source data are provided with this paper.

## Code availability

The code of the in-house clonotyping script can be found in the Supplementary Information.

## References

[1] Crowe, J. E. Human antibodies for viral infections. *Annu. Rev. Immunol.* **40**, 349–386 (2022). 35113730 10.1146/annurev-immunol-042718-041309

[2] Pantaleo, G., Correia, B., Fenwick, C., Joo, V. S. & Perez, L. Antibodies to combat viral infections: development strategies and progress. *Nat. Rev. Drug Discov.* **21**, 676–696 (2022). 35725925 10.1038/s41573-022-00495-3 PMC9207876

[3] Pedrioli, A. & Oxenius, A. Single B cell technologies for monoclonal antibody discovery. *Trends Immunol.* **42**, 1143–1158 (2021). 34743921 10.1016/j.it.2021.10.008

[4] Hansen, J. et al. Studies in humanized mice and convalescent humans yield a SARS-CoV-2 antibody cocktail. *Science* **369**, 1010–1014 (2020). 32540901 10.1126/science.abd0827 PMC7299284

[5] Corti, D. et al. Protective monotherapy against lethal Ebola virus infection by a potently neutralizing antibody. *Science* **351**, 1339–1342 (2016). 26917593 10.1126/science.aad5224

[6] Scheid, J. F. et al. Broad diversity of neutralizing antibodies isolated from memory B cells in HIV-infected individuals. *Nature* **458**, 636–640 (2009). 19287373 10.1038/nature07930

[7] Weisel, F. J., Zuccarino-Catania, G. V., Chikina, M. & Shlomchik, M. J. A temporal switch in the germinal center determines differential output of memory B and plasma cells. *Immunity* **44**, 116–130 (2016). 26795247 10.1016/j.immuni.2015.12.004 PMC4724390

[8] Phan, T. G. et al. High affinity germinal center B cells are actively selected into the plasma cell compartment. *J. Exp. Med.* **203**, 2419–2424 (2006). 17030950 10.1084/jem.20061254 PMC2118125

[9] Viant, C. et al. Antibody affinity shapes the choice between memory and germinal center B cell fates. *Cell* **183**, 1298–1311 (2020). 33125897 10.1016/j.cell.2020.09.063 PMC7722471

[10] Nutt, S. L., Hodgkin, P. D., Tarlinton, D. M. & Corcoran, L. M. The generation of antibody-secreting plasma cells. *Nat. Rev. Immunol.* **15**, 160–171 (2015). 25698678 10.1038/nri3795

[11] Bondt, A. et al. Human plasma IgG1 repertoires are simple, unique, and dynamic. *Cell Syst.* **12**, 1131–1143 (2021). 34613904 10.1016/j.cels.2021.08.008 PMC8691384

[12] Pinto, D. et al. A functional BCR in human IgA and IgM plasma cells. *Blood* **121**, 4110–4114 (2013). 23550036 10.1182/blood-2012-09-459289

[13] Wrammert, J. et al. Rapid cloning of high-affinity human monoclonal antibodies against influenza virus. *Nature* **453**, 667–671 (2008). 18449194 10.1038/nature06890 PMC2515609

[14] Smith, K. et al. Rapid generation of fully human monoclonal antibodies specific to a vaccinating antigen. *Nat. Protoc.* **4**, 372–384 (2009). 19247287 10.1038/nprot.2009.3 PMC2750034

[15] Czerkinsky, C. C., Nilsson, L.-Å., Nygren, H., Ouchterlony, Ö. & Tarkowski, A. A solid-phase enzyme-linked immunospot (ELISPOT) assay for enumeration of specific antibody-secreting cells. *J. Immunol. Methods* **65**, 109–121 (1983). 6361139 10.1016/0022-1759(83)90308-3

[16] Shembekar, N., Hu, H., Eustace, D. & Merten, C. A. Single-cell droplet microfluidic screening for antibodies specifically binding to target cells. *Cell Rep.* **22**, 2206–2215 (2018). 29466744 10.1016/j.celrep.2018.01.071 PMC5842027

[17] Gérard, A. et al. High-throughput single-cell activity-based screening and sequencing of antibodies using droplet microfluidics. *Nat. Biotechnol.* **38**, 715–721 (2020). 32231335 10.1038/s41587-020-0466-7

[18] Mazutis, L. et al. Single-cell analysis and sorting using droplet-based microfluidics. *Nat. Protoc.* **8**, 870–891 (2013). 23558786 10.1038/nprot.2013.046 PMC4128248

[19] Ding, R. et al. Rapid isolation of antigen-specific B-cells using droplet microfluidics. *RSC Adv.* **10**, 27006–27013 (2020). 35515810 10.1039/d0ra04328a PMC9055518

[20] Broketa, M. & Bruhns, P. Single-cell technologies for the study of antibody-secreting cells. *Front. Immunol.* **12**, 1–9 (2022). 10.3389/fimmu.2021.821729 PMC8841722 35173713

[21] Wang, B. et al. Functional interrogation and mining of natively paired human V_H_:V_L_ antibody repertoires. *Nat. Biotechnol.* **36**, 152–155 (2018). 29309060 10.1038/nbt.4052 PMC5801115

[22] Dekosky, B. J. et al. High-throughput sequencing of the paired human immunoglobulin heavy and light chain repertoire. *Nat. Biotechnol.* **31**, 166–169 (2013). 23334449 10.1038/nbt.2492 PMC3910347

[23] Setliff, I. et al. High-throughput mapping of B cell receptor sequences to antigen specificity. *Cell* **179**, 1636–1646 (2019). 31787378 10.1016/j.cell.2019.11.003 PMC7158953

[24] Zost, S. J. et al. Rapid isolation and profiling of a diverse panel of human monoclonal antibodies targeting the SARS-CoV-2 spike protein. *Nat. Med.* **26**, 1422–1427 (2020). 32651581 10.1038/s41591-020-0998-x PMC8194108

[25] Keppler, A. et al. Labeling of fusion proteins of O6-alkylguanine-DNA alkyltransferase with small molecules in vivo and in vitro. *Methods* **32**, 437–444 (2004). 15003606 10.1016/j.ymeth.2003.10.007

[26] Pleiner, T., Bates, M. & Görlich, D. A toolbox of anti-mouse and anti-rabbit IgG secondary nanobodies. *J. Cell Biol.* **217**, 1143–1154 (2018). 29263082 10.1083/jcb.201709115 PMC5839796

[27] Ossipow, V. & Fischer, N. (eds). *Methods in Molecular Biology* Vol. 1131, pp. 297–314 (Humana Press, 2014).

[28] Pernodet, N., Maaloum, M. & Tinland, B. Pore size of agarose gels by atomic force microscopy. *Electrophoresis* **18**, 55–58 (1997). 9059821 10.1002/elps.1150180111

[29] Pluen, A., Netti, P. A., Jain, R. K. & Berk, D. A. Diffusion of macromolecules in agarose gels: comparison of linear and globular configurations. *Biophys. J.* **77**, 542–552 (1999). 10388779 10.1016/S0006-3495(99)76911-0 PMC1300351

[30] Hibi, T. & Dosch, H.-M. Limiting dilution analysis of the B cell compartment in human bone marrow. *Eur. J. Immunol.* **16**, 139–145 (1986). 2869953 10.1002/eji.1830160206

[31] Eyer, K. et al. Single-cell deep phenotyping of IgG-secreting cells for high-resolution immune monitoring. *Nat. Biotechnol.* **35**, 977–982 (2017). 28892076 10.1038/nbt.3964

[32] Manz, R., Assenmacher, M., Pflüger, E., Miltenyi, S. & Radbruch, A. Analysis and sorting of live cells according to secreted molecules, relocated to a cell-surface affinity matrix. *Proc. Natl Acad. Sci. USA* **92**, 1921–1925 (1995). 7892200 10.1073/pnas.92.6.1921 PMC42394

[33] Pinder, C. L. et al. Isolation and characterization of antigen-specific plasmablasts using a novel flow cytometry-based Ig capture assay. *J. Immunol.* **199**, 4180–4188 (2017). 29118244 10.4049/jimmunol.1701253 PMC5720343

[34] Pracht, K. et al. A new staining protocol for detection of murine antibody-secreting plasma cell subsets by flow cytometry. *Eur. J. Immunol.* **47**, 1389–1392 (2017). 28608550 10.1002/eji.201747019

[35] Raybould, M. I. J., Kovaltsuk, A., Marks, C. & Deane, C. M. CoV-AbDab: the coronavirus antibody database. *Bioinformatics* **37**, 734–735 (2021). 32805021 10.1093/bioinformatics/btaa739 PMC7558925

[36] Alsoussi, W. B. et al. A potently neutralizing antibody protects mice against SARS-CoV-2 infection. *J. Immunol.* **205**, 915–922 (2020). 32591393 10.4049/jimmunol.2000583 PMC7566074

[37] Gerber, P. P. et al. A protease-activatable luminescent biosensor and reporter cell line for authentic SARS-CoV-2 infection. *PLoS Pathog.* **18**, e1010265 (2022). 35143592 10.1371/journal.ppat.1010265 PMC8865646

[38] Bergamaschi, L. et al. Longitudinal analysis reveals that delayed bystander CD8^+^ T cell activation and early immune pathology distinguish severe COVID-19 from mild disease. *Immunity* **54**, 1257–1275 (2021). 34051148 10.1016/j.immuni.2021.05.010 PMC8125900

[39] Van der Klaauw, A. A. et al. Accelerated waning of the humoral response to COVID-19 vaccines in obesity. *Nat. Med.* **29**, 1146–1154 (2023). 37169862 10.1038/s41591-023-02343-2 PMC10202802

[40] Errico, J. M. et al. Structural mechanism of SARS-CoV-2 neutralization by two murine antibodies targeting the RBD. *Cell Rep.* **37**, 109881 (2021). 34655519 10.1016/j.celrep.2021.109881 PMC8498651

[41] Turner, J. S. et al. SARS-CoV-2 mRNA vaccines induce persistent human germinal centre responses. *Nature* **596**, 109–113 (2021). 34182569 10.1038/s41586-021-03738-2 PMC8935394

[42] Amanat, F. et al. SARS-CoV-2 mRNA vaccination induces functionally diverse antibodies to NTD, RBD, and S2. *Cell* **184**, 3936–3948 (2021). 34192529 10.1016/j.cell.2021.06.005 PMC8185186

[43] Ettinger, R. et al. IL-21 induces differentiation of human naive and memory B cells into antibody-secreting plasma cells. *J. Immunol.* **175**, 7867–7879 (2005). 16339522 10.4049/jimmunol.175.12.7867

[44] Kyu, S. Y. et al. Frequencies of human influenza-specific antibody secreting cells or plasmablasts post vaccination from fresh and frozen peripheral blood mononuclear cells. *J. Immunol. Methods* **340**, 42–47 (2009). 18996127 10.1016/j.jim.2008.09.025 PMC4690209

[45] Li, D. et al. In vitro and in vivo functions of SARS-CoV-2 infection-enhancing and neutralizing antibodies. *Cell* **184**, 4203–4219 (2021). 34242577 10.1016/j.cell.2021.06.021 PMC8232969

[46] Cho, A. et al. Anti-SARS-CoV-2 receptor-binding domain antibody evolution after mRNA vaccination. *Nature* **600**, 517–522 (2021). 34619745 10.1038/s41586-021-04060-7 PMC8674133

[47] Du, S. et al. Structurally resolved SARS-CoV-2 antibody shows high efficacy in severely infected hamsters and provides a potent cocktail pairing strategy. *Cell* **183**, 1013–1023 (2020). 32970990 10.1016/j.cell.2020.09.035 PMC7489885

[48] Wang, Z. et al. mRNA vaccine-elicited antibodies to SARS-CoV-2 and circulating variants. *Nature* **592**, 616–622 (2021). 33567448 10.1038/s41586-021-03324-6 PMC8503938

[49] Brouwer, P. J. M. M. et al. Potent neutralizing antibodies from COVID-19 patients define multiple targets of vulnerability. *Science* **369**, 643–650 (2020). 32540902 10.1126/science.abc5902 PMC7299281

[50] Reincke, M. S. et al. SARS-CoV-2 Beta variant infection elicits potent lineage-specific and cross-reactive antibodies. *Science* **375**, 782–787 (2022). 35076281 10.1126/science.abm5835 PMC8939768

[51] Robinson, S. A. et al. Epitope profiling using computational structural modelling demonstrated on coronavirus-binding antibodies. *PLoS Comput. Biol.* **17**, 1–20 (2021). 10.1371/journal.pcbi.1009675 PMC8700021 34898603

[52] Dejnirattisai, W. et al. SARS-CoV-2 Omicron-B.1.1.529 leads to widespread escape from neutralizing antibody responses. *Cell* **185**, 467–484 (2022). 35081335 10.1016/j.cell.2021.12.046 PMC8723827

[53] Planas, D. et al. Considerable escape of SARS-CoV-2 Omicron to antibody neutralization. *Nature* **602**, 671–675 (2022). 35016199 10.1038/s41586-021-04389-z

[54] Sheward, D. J. et al. Structural basis of broad SARS-CoV-2 cross-neutralization by affinity-matured public antibodies. *Cell Rep. Med.* **5**, 101577 (2024). 38761799 10.1016/j.xcrm.2024.101577 PMC11228396

[55] Shiakolas, A. R. et al. Efficient discovery of SARS-CoV-2-neutralizing antibodies via B cell receptor sequencing and ligand blocking. *Nat. Biotechnol.* **40**, 1270–1275 (2022). 35241839 10.1038/s41587-022-01232-2 PMC9378442

[56] Ellebedy, A. H. et al. Defining antigen-specific plasmablast and memory B cell subsets in human blood after viral infection or vaccination. *Nat. Immunol.* **17**, 1226–1234 (2016). 27525369 10.1038/ni.3533 PMC5054979

[57] Rouers, A. et al. CD27hiCD38hi plasmablasts are activated B cells of mixed origin with distinct function. *iScience* **24**, 102482 (2021). 34113823 10.1016/j.isci.2021.102482 PMC8169951

[58] Garimilla, S. et al. Differential transcriptome and development of human peripheral plasma cell subsets. *JCI Insight* **4**, e126732 (2019). 31045577 10.1172/jci.insight.126732 PMC6538338

[59] Jourdan, M. et al. An in vitro model of differentiation of memory B cells into plasmablasts and plasma cells including detailed phenotypic and molecular characterization. *Blood* **114**, 5173–5181 (2009). 19846886 10.1182/blood-2009-07-235960 PMC2834398

[60] Caraux, A. et al. Circulating human B and plasma cells. Age-associated changes in counts and detailed characterization of circulating normal CD138^−^ and CD138^+^ plasma cells. *Haematologica* **95**, 1016–1020 (2010). 20081059 10.3324/haematol.2009.018689 PMC2878802

[61] Ekiert, D. C. et al. A highly conserved neutralizing epitope on group 2 influenza A viruses. *Science* **333**, 843–850 (2011). 21737702 10.1126/science.1204839 PMC3210727

[62] Farady, C. J., Sellers, B. D., Jacobson, M. P. & Craik, C. S. Improving the species cross-reactivity of an antibody using computational design. *Bioorg. Med. Chem. Lett.* **19**, 3744–3747 (2009). 19477127 10.1016/j.bmcl.2009.05.005 PMC2724971

[63] Proetzel, G. & Ebersbach, H. (eds). *Methods in Molecular Biology*, pp. 117–135 (Humana Press, 2012).

[64] Kenney, J. S., Gray, F., Ancel, M.-H. & Dunne, J. F. Production of monoclonal antibodies using a secretion capture report web. *Biotechnology* **13**, 787–790 (1995). 9634809 10.1038/nbt0895-787

[65] Powell, K. T. & Weaver, J. C. Gel microdroplets and flow cytometry: rapid determination of antibody secretion by individual cells within a cell population. *Nat. Biotechnol.* **8**, 333–337 (1990). 10.1038/nbt0490-333 1366534

[66] de Rutte, J. et al. Suspendable hydrogel nanovials for massively parallel single-cell functional analysis and sorting. *ACS Nano* **16**, 7242–7257 (2022). 35324146 10.1021/acsnano.1c11420 PMC9869715

[67] Akselband, Y., Moen, P. T. & McGrath, P. Isolation of rare isotype switch variants in hybridoma cell lines using an agarose gel microdrop-based protein secretion assay. *Assay Drug Dev. Technol.* **1**, 619–626 (2003). 15090234 10.1089/154065803770380977

[68] Gaa, R. et al. Versatile and rapid microfluidics-assisted antibody discovery. *MAbs* **13**, 1978130 (2021). 34586015 10.1080/19420862.2021.1978130 PMC8489958

[69] Winters, A. et al. Rapid single B cell antibody discovery using nanopens and structured light. *MAbs* **11**, 1025–1035 (2019). 31185801 10.1080/19420862.2019.1624126 PMC6748590

[70] Santra, T. & Tseng, F. (eds). *Handbook of Single Cell Technologies*, pp. 1–23 (Springer, 2020).

[71] Ogunniyi, A. O., Story, C. M., Papa, E., Guillen, E. & Love, J. C. Screening individual hybridomas by microengraving to discover monoclonal antibodies. *Nat. Protoc.* **4**, 767–782 (2009). 19528952 10.1038/nprot.2009.40 PMC4034573

[72] Asrat, S. et al. TRAPnSeq allows high-throughput profiling of antigen-specific antibody-secreting cells. *Cell Rep. Methods* **3**, 100522 (2023). 37533642 10.1016/j.crmeth.2023.100522 PMC10391570

[73] Im, M. et al. Comparative quantitative analysis of cluster of differentiation 45 antigen expression on lymphocyte subsets. *Korean J. Lab. Med.* **31**, 148–153 (2011). 21779186 10.3343/kjlm.2011.31.3.148 PMC3129343

[74] Raybould, M. I. J., Rees, A. R. & Deane, C. M. Current strategies for detecting functional convergence across B-cell receptor repertoires. *MAbs* **13**, 1996732 (2021). 34781829 10.1080/19420862.2021.1996732 PMC8604390

[75] Jennewein, M. F. & Alter, G. The immunoregulatory roles of antibody glycosylation. *Trends Immunol.* **38**, 358–372 (2017). 28385520 10.1016/j.it.2017.02.004

[76] Turchaninova, M. A. et al. High-quality full-length immunoglobulin profiling with unique molecular barcoding. *Nat. Protoc.* **11**, 1599–1616 (2016). 27490633 10.1038/nprot.2016.093

[77] Zurek, P. J., Knyphausen, P., Neufeld, K., Pushpanath, A. & Hollfelder, F. UMI-linked consensus sequencing enables phylogenetic analysis of directed evolution. *Nat. Commun.* **11**, 6023 (2020). 33243970 10.1038/s41467-020-19687-9 PMC7691348

[78] Walker, L. M. et al. High-throughput B cell epitope determination by next-generation sequencing. *Front. Immunol.* **13**, 855772 (2022). 35401559 10.3389/fimmu.2022.855772 PMC8984479

[79] Khetan, R. et al. Current advances in biopharmaceutical informatics: guidelines, impact and challenges in the computational developability assessment of antibody therapeutics. *MAbs* **14**, 2020082 (2022). 35104168 10.1080/19420862.2021.2020082 PMC8812776

[80] Carlson, C. J. et al. Climate change increases cross-species viral transmission risk. *Nature* **607**, 555–562 (2022). 35483403 10.1038/s41586-022-04788-w

[81] Xia, Y. & Whitesides, G. M. Soft lithography. *Angew. Chem. Int. Ed. Engl.* **37**, 550–575 (1998). 29711088 10.1002/(SICI)1521-3773(19980316)37:5<550::AID-ANIE550>3.0.CO;2-G

[82] Hermans, W. J. J., Ten Haaft, M. R. & Overweel, A. Method for affinity purification. World patent WO2006059904A1. patents.google.com/patent/WO2006059904A1/enIt (2004).

[83] Gibson, D. G. et al. Enzymatic assembly of DNA molecules up to several hundred kilobases. *Nat. Methods* **6**, 343–345 (2009). 19363495 10.1038/nmeth.1318

[84] Haughton, G., Lanier, L. L. & Babcock, G. F. The murine kappa light chain shift. *Nature* **275**, 154–157 (1978). 99664 10.1038/275154a0

[85] Lambert, C. R., Nijsure, D., Huynh, V. & Wylie, R. G. Hydrogels with reversible chemical environments for in vitro cell culture. *Biomed. Mater.* **13**, 045002 (2018). 29508767 10.1088/1748-605X/aab45d

[86] Schindelin, J. et al. Fiji: an open-source platform for biological-image analysis. *Nat. Methods* **9**, 676–682 (2012). 22743772 10.1038/nmeth.2019 PMC3855844

[87] Von Boehmer, L. et al. Sequencing and cloning of antigen-specific antibodies from mouse memory B cells. *Nat. Protoc.* **11**, 1908–1923 (2016). 27658009 10.1038/nprot.2016.102

[88] Bagnoli, J. W. et al. Sensitive and powerful single-cell RNA sequencing using mcSCRB-seq. *Nat. Commun.* **9**, 2937 (2018). 30050112 10.1038/s41467-018-05347-6 PMC6062574

[89] Karbaschi, M., Shahi, P. & Abate, A. R. Rapid, chemical-free breaking of microfluidic emulsions with a hand-held antistatic gun. *Biomicrofluidics* **11**, 044107 (2017). 28794817 10.1063/1.4995479 PMC5519397

[90] Hagemann-Jensen, M. et al. Single-cell RNA counting at allele and isoform resolution using Smart-seq3. *Nat. Biotechnol.* **38**, 708–714 (2020). 32518404 10.1038/s41587-020-0497-0

[91] Gieselmann, L. et al. Effective high-throughput isolation of fully human antibodies targeting infectious pathogens. *Nat. Protoc.* **16**, 3639–3671 (2021). 34035500 10.1038/s41596-021-00554-w

[92] Brochet, X., Lefranc, M.-P. & Giudicelli, V. IMGT/V-QUEST: the highly customized and integrated system for IG and TR standardized V–J and V–D–J sequence analysis. *Nucleic Acids Res.* **36**, W503–W508 (2008). 18503082 10.1093/nar/gkn316 PMC2447746

[93] Dodev, T. S. et al. A tool kit for rapid cloning and expression of recombinant antibodies. *Sci. Rep.* **4**, 5885 (2015). 10.1038/srep05885 PMC4115235 25073855

[94] Gottschalk, P. G. & Dunn, J. R. The five-parameter logistic: a characterization and comparison with the four-parameter logistic. *Anal. Biochem.* **343**, 54–65 (2005). 15953581 10.1016/j.ab.2005.04.035

[95] Daly, J. L. et al. Neuropilin-1 is a host factor for SARS-CoV-2 infection. *Science* **370**, 861–865 (2020). 33082294 10.1126/science.abd3072 PMC7612957

[96] Patterson, E. I. et al. Methods of inactivation of SARS-CoV-2 for downstream biological assays. *J. Infect. Dis.* **222**, 1462–1467 (2020). 32798217 10.1093/infdis/jiaa507 PMC7529010

[97] Meng, B. et al. Altered TMPRSS2 usage by SARS-CoV-2 Omicron impacts infectivity and fusogenicity. *Nature* **603**, 706–714 (2022). 35104837 10.1038/s41586-022-04474-x PMC8942856

[98] McCoy, A. J. et al. Phaser crystallographic software. *J. Appl. Crystallogr.* **40**, 658–674 (2007). 19461840 10.1107/S0021889807021206 PMC2483472

[99] Afonine, P. V. et al. Towards automated crystallographic structure refinement with phenix.refine. *Acta Crystallogr. D Biol. Crystallogr.* **68**, 352–367 (2012). 22505256 10.1107/S0907444912001308 PMC3322595

[100] Emsley, P., Lohkamp, B., Scott, W. G. & Cowtan, K. Features and development of Coot. *Acta Crystallogr. D Biol. Crystallogr.* **66**, 486–501 (2010). 20383002 10.1107/S0907444910007493 PMC2852313

