# Supplementary material for: Rapid discovery of monoclonal antibodies by microfluidics-enabled FACS of single pathogen-specific antibody-secreting cells
Source: Nat Biotechnol. 2024 Aug 14;43(6):960–70. doi: 10.1038/s41587-024-02346-5 (PMC12167710; doi:10.1038/s41587-024-02346-5)
Supplement: Supplementary file 1 — Supplementary Figs. 1–9. [file 41587_2024_2346_MOESM1_ESM.pdf]

nature biotechnology

Supplementary information https://doi.org/10.1038/s41587-024-02346-5

Rapid discovery of monoclonal antibodies by microfluidics-enabled FACS of single pathogen-specific antibody-secreting cells

In the format provided by the authors and unedited

Table of Contents

Supplementary Fig. 1 Design of microfluidic chip. 2

Supplementary Fig. 2 Gating strategy for viability time course experiments. 3

Supplementary Fig. 3 Gating strategy for secretion time course experiments........ 4

Supplementary Fig. 4 Confocal analysis of encapsulated OVA-specific mouse plasma cells. . 5

Supplementary Fig. 5 Conformational changes in RBD and in the RBD:Fab complexes. 6

Supplementary Fig. 6 Binding affinities of human anti-RBD and anti-S1 antibodies........ 7

Supplementary Fig. 7 Neutralisation of SARS-CoV-2 by human antibodies........ 8

Supplementary Fig. 8 Epitope binning of selected human antibodies. 9

Supplementary Fig. 9: Considerations for adjusting the stringency of the screen based on the antigen concentration. 10

References........ 11

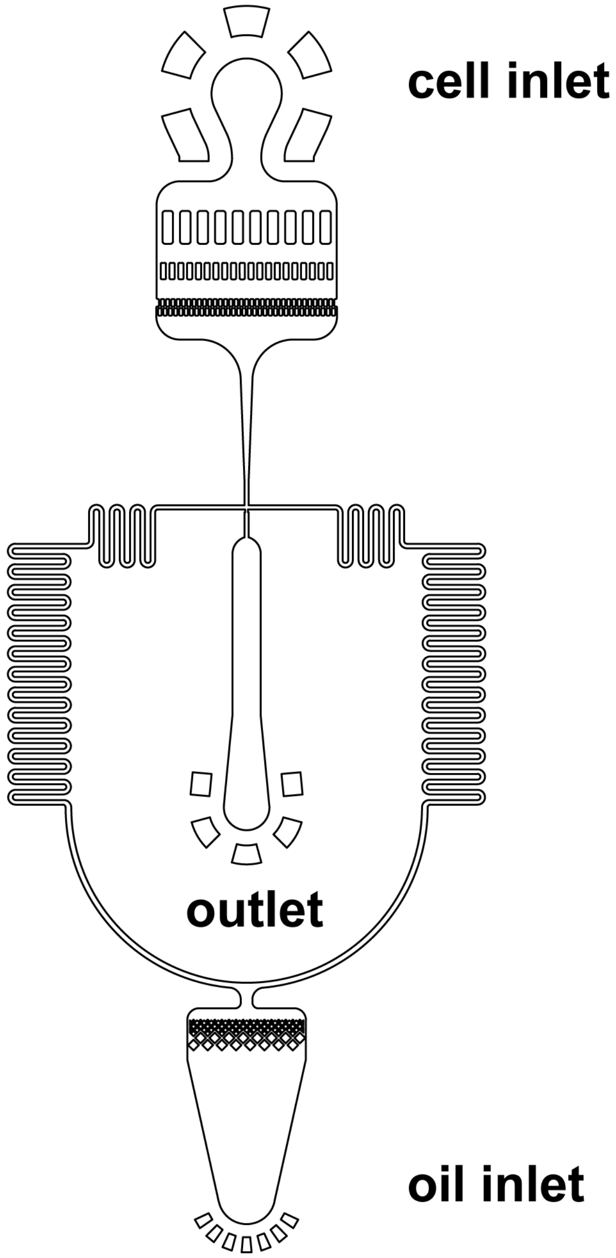

Supplementary Fig. 1 Design of microfluidic chip.

The channel layout is available as a .dxf file from https://openwetware.org/wiki/DropBase.

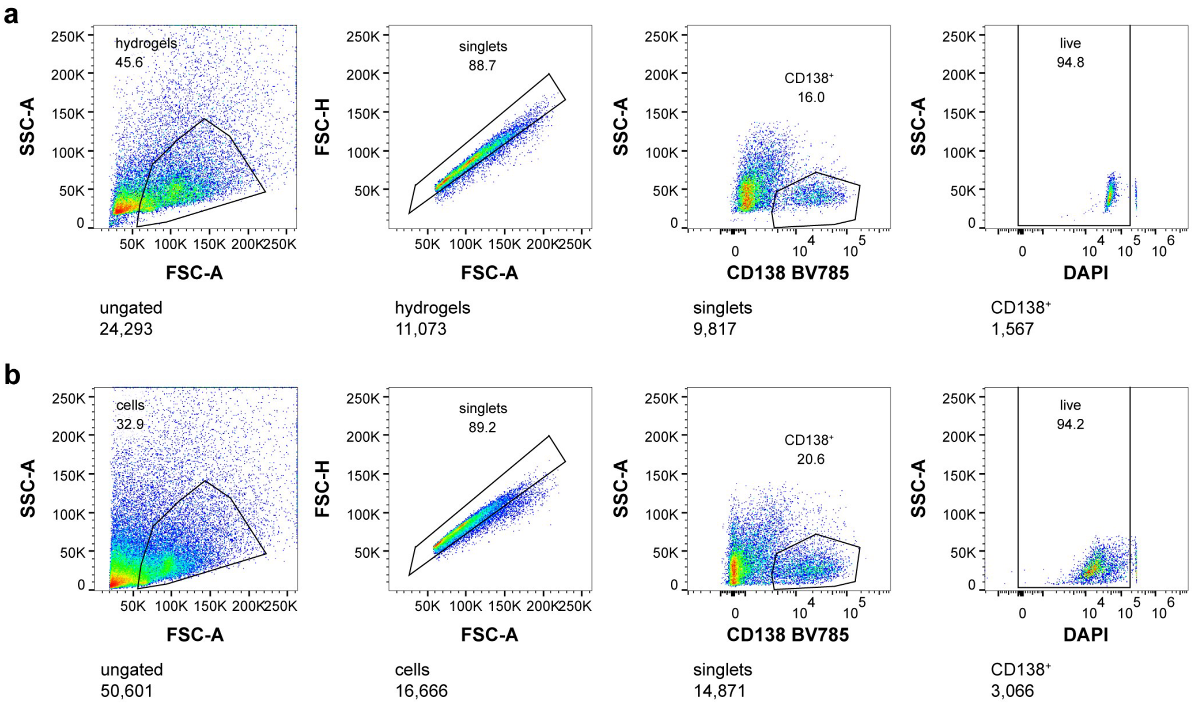

Supplementary Fig. 2 Gating strategy for viability time course experiments.

(a) Encapsulated cells. (b) Non-encapsulated cells. The fraction of live cells in the plasma cell population was gated as $CD138^{+}/DAPI^{-}$. In contrast to the sorting experiments, encapsulated cells were gated on linear scatter parameters to enable better comparison with non-encapsulated cells.

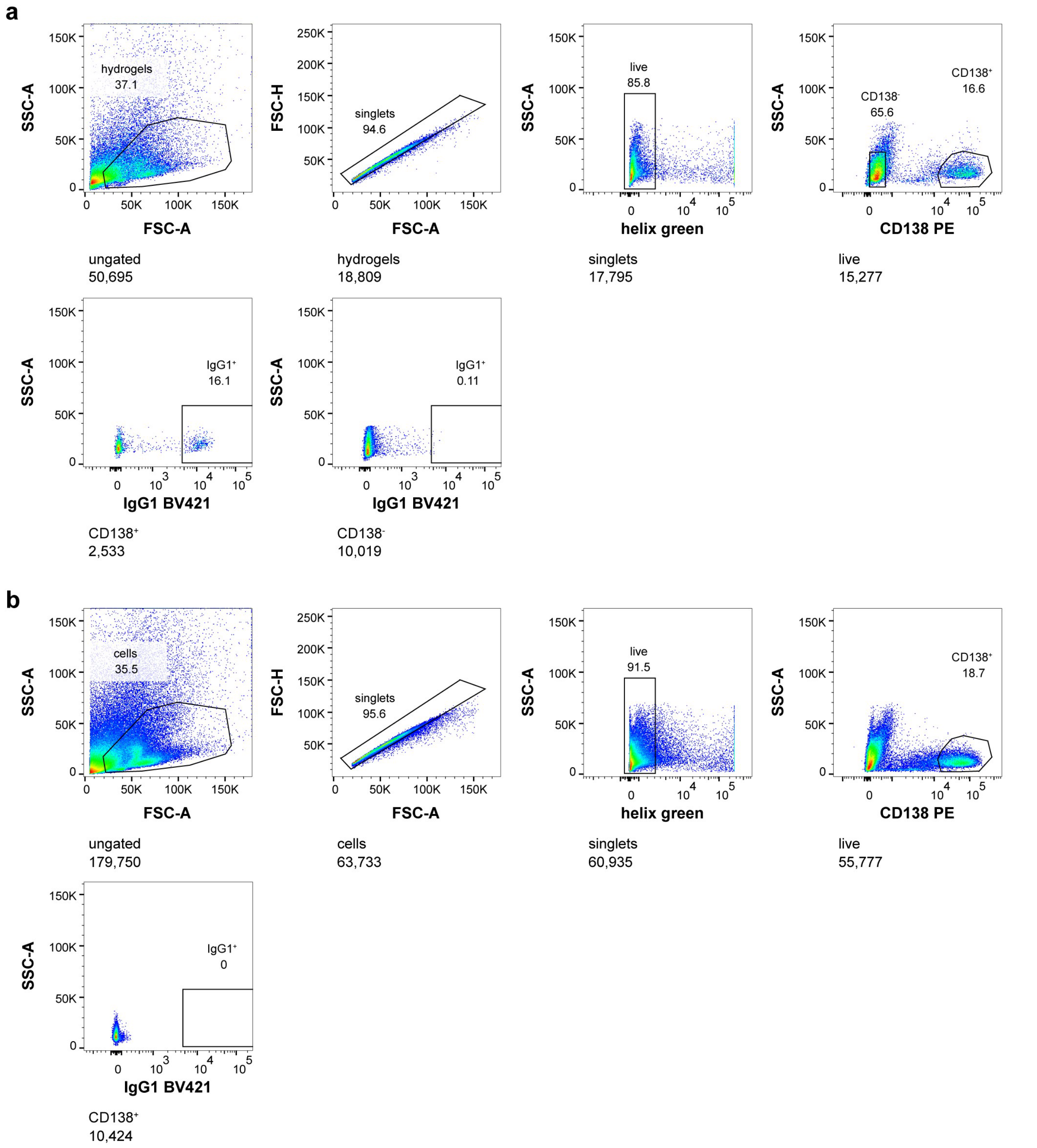

Supplementary Fig. 3 Gating strategy for secretion time course experiments.

(a) Encapsulated cells after 1 h 45 min of incubation. (b) Non-encapsulated cells after 1h 45 min of incubation. IgG1-secreting plasma cells were gated as (live/CD138$^+$/IgG1$^+$). To assess background levels, the IgG1 signal of the CD138$^-$ population was also analysed. In contrast to the sorting experiments, hydrogels were gated on linear scatter parameters to enable better comparison with non-encapsulated cells.

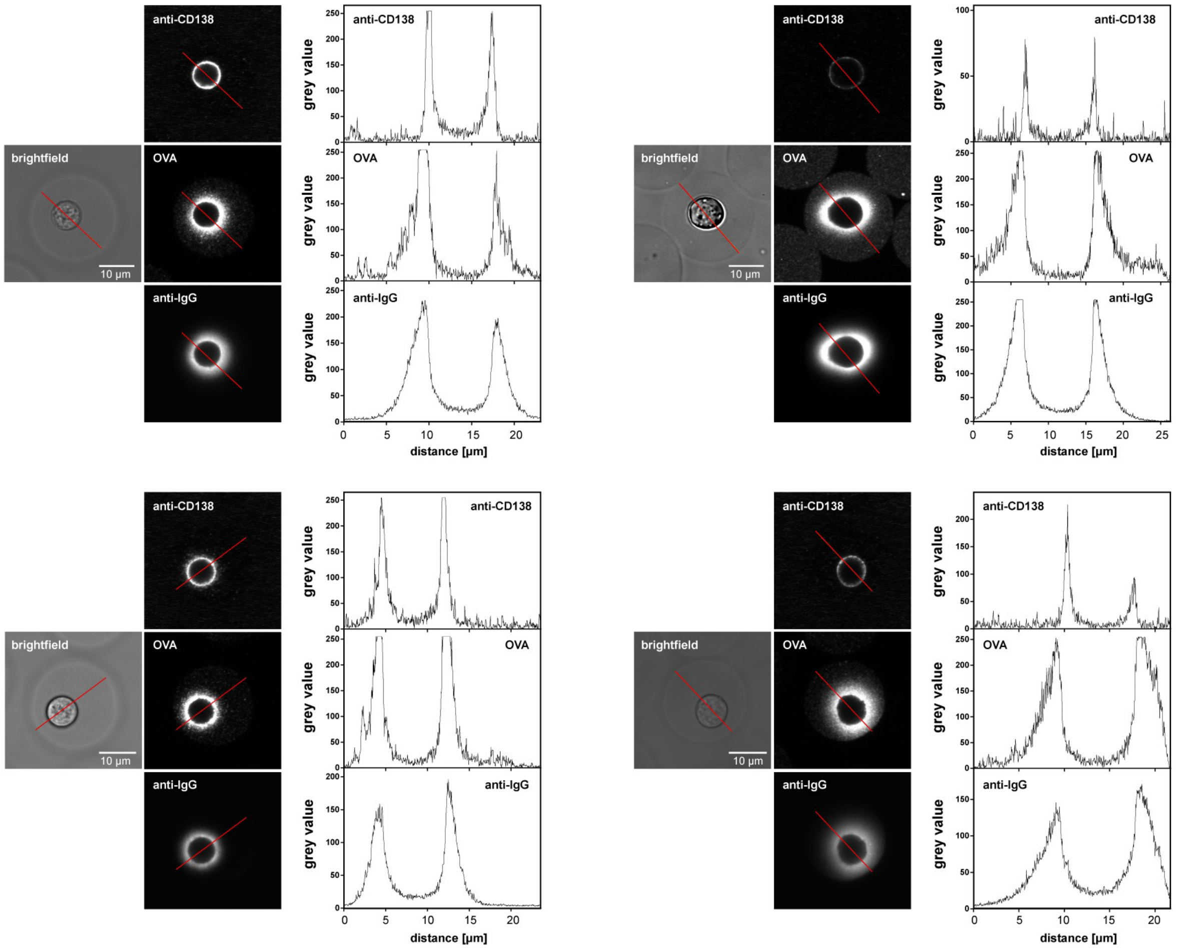

Supplementary Fig. 4 Confocal analysis of encapsulated OVA-specific mouse plasma cells.

Additional confocal images of encapsulated plasma cells showing the spatial distribution of CD138 (AF647), IgG (AF405) and OVA (AF55) within the VHH-functionalised BG-agarose hydrogel. The cross section for analysis is indicated by a red line. The graphs display the pixel intensity along the red line from left to right. Representative images of over 40 images obtained from two biological replicates are shown.

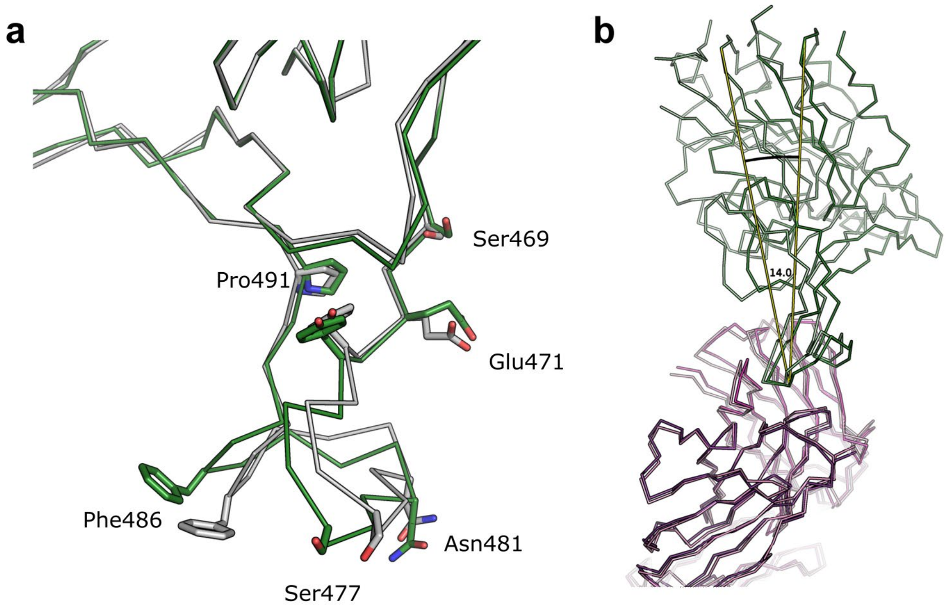

Supplementary Fig. 5 Conformational changes in RBD and in the RBD:Fab complexes.

(a) Superposition of RBD (chain C, green) from our Fab complex and RBD in an ACE2 complex (PDB: 6m0j, grey), showing how the loop is shifted by a few ångströms in the Fab complex with some of the sidechains from the moved loop shown as sticks. (b) Superposition of the two Fab fragments (dark and light purple) found in the asymmetric unit of our structure highlighting the circa 14° tilt of RBD relative to the Fab fragment in the second complex (RBD chain F, light green), in comparison with the other RBD (which is used in the analysis of the main paper; dark green), most likely due to crystal contacts.

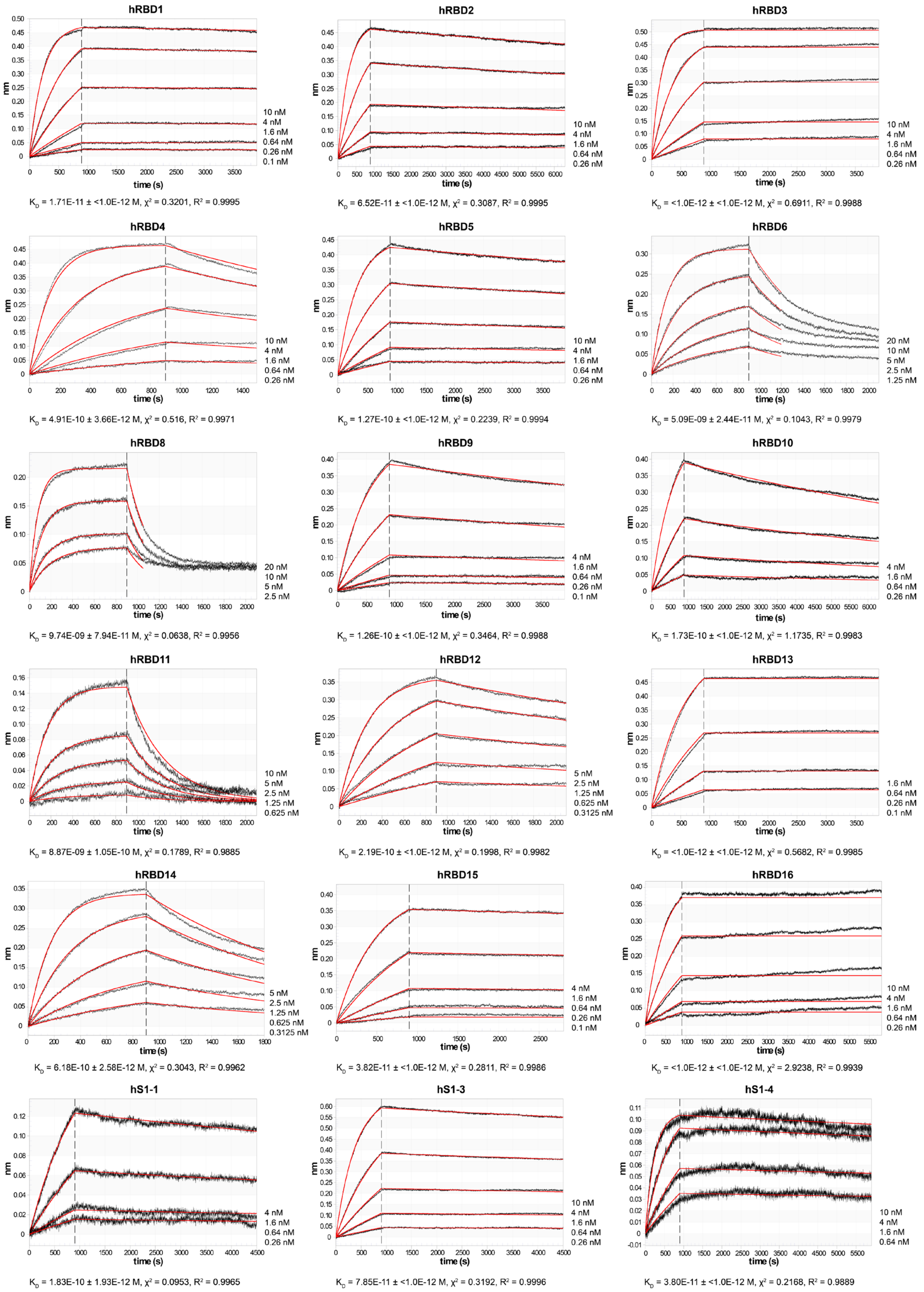

Supplementary Fig. 6 Binding affinities of human anti-RBD and anti-S1 antibodies.

Dissociation constant (K_D) and fitting parameters (χ^2, R^2) are given below the respective sensorgrams.

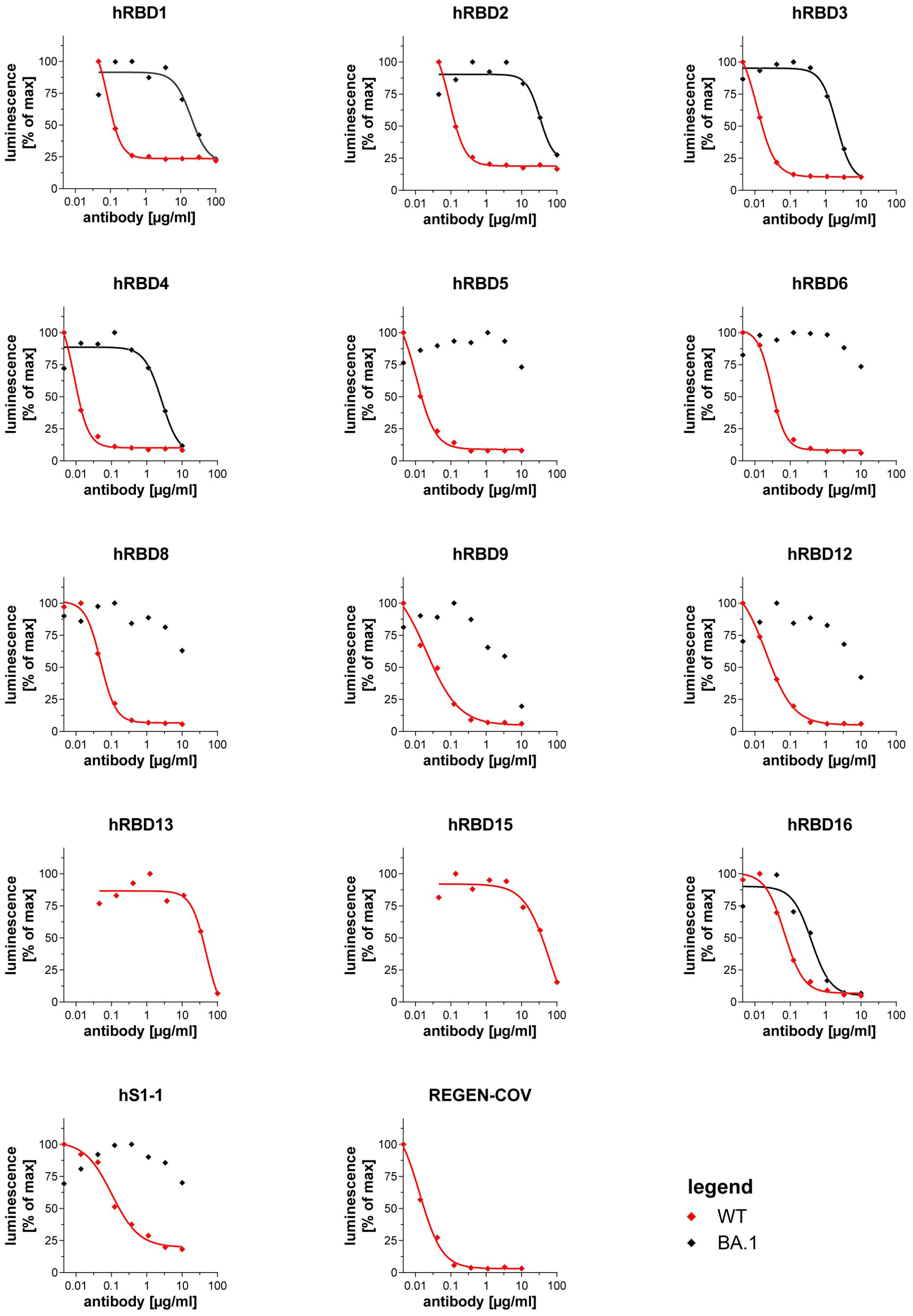

Supplementary Fig. 7 Neutralisation of SARS-CoV-2 by human antibodies.

Wildtype or Omicron BA.1 SARS-CoV-2 (MOI=0.01) was pre-incubated with a 3-fold dilution series of each antibody or the REGEN-COV (Ronapreve) antibody cocktail, then used to infect luminescent reporter cells. Levels of infection after 24 h were quantified as % of maximum luminescence. Mean values of two technical replicates are shown. IC_{50}s calculated from these titration curves are depicted in **Fig. 3e**. The titration curve for hS1-1 against WT SARS-CoV-2 is also shown in **Extended Data Fig. 9c**.

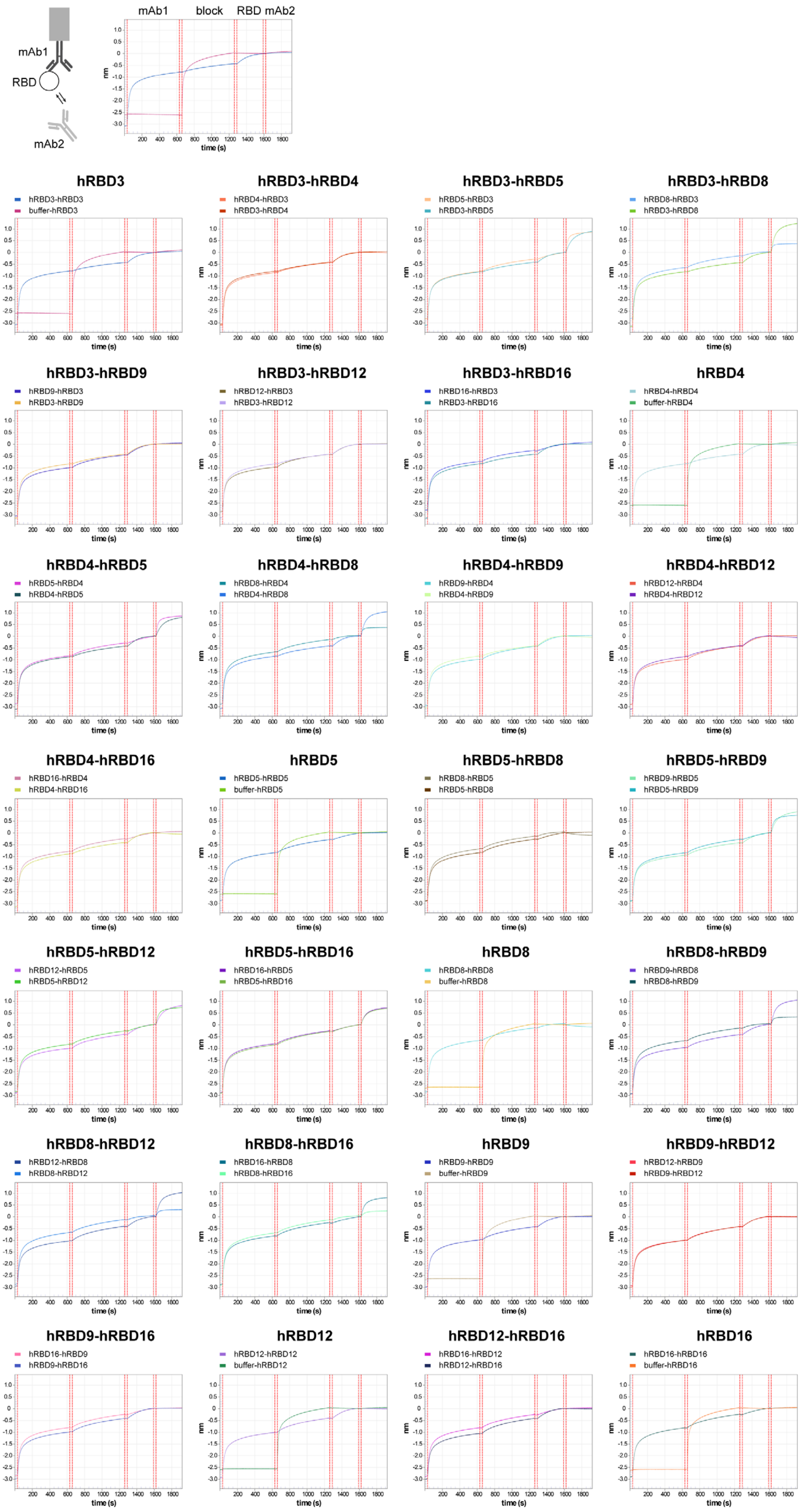

Supplementary Fig. 8 Epitope binning of selected human antibodies.

Antibodies were immobilised on anti-human IgG Fc-capture biosensors followed by blocking of free capture sites with a human IgG1 isotype control and incubation with RBD. Biosensors were then probed with the second antibody.

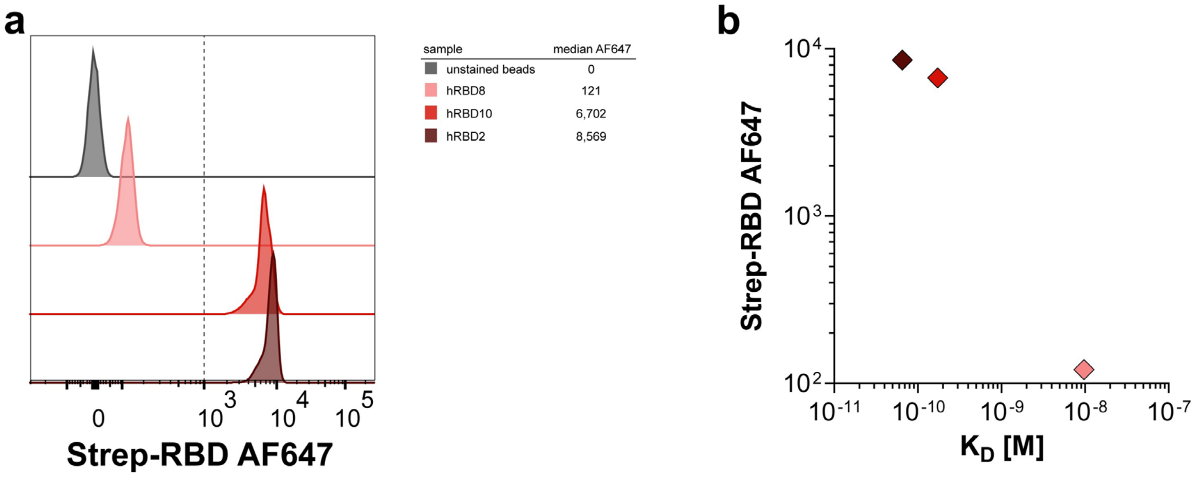

Supplementary Fig. 9: Considerations for adjusting the stringency of the screen based on the antigen concentration.

(a) Flow cytometry experiment to determine the fluorescence signal generated by human anti-RBD antibodies with different affinities. VHH-functionalised BG-agarose beads were incubated with recombinant human anti-RBD antibodies and stained with 1 nM of strep-RBD AF647 prior to analysis. The same number of antibodies was immobilised per bead. Antigen tetramers were chosen to reflect the conditions used in the initial screen. The dotted line represents a hypothetical threshold for selection; hRBD8 (lower affinity antibody) would not meet this threshold at this antigen concentration. (b) Relationship between antibody affinity and antigen signal at 1 nM of antigen. Median fluorescence values were plotted against the K_D values determined by BLI. At least 8,767 beads were analysed per condition.

To select a certain range of antibody affinities in the screen, the concentration of antigen can be adjusted. At higher antigen concentrations, a broader range of affinities will be selected while lower antigen concentrations will lead to a more stringent selection. Further stringency adjustments can be made by employing monomeric (selection of antibodies with higher affinity) or multimeric (selection of antibodies with higher avidity) antigens in the screen. To correct for different levels of antibody secretion, the antigen signal can be normalised to the IgG signal. In the above example, we chose an antigen concentration of 1 nM (compared to 40 nM used in the initial screen) to show that discrimination between the antibody with the lowest affinity (hRBD8, K_D = 9.7 nM) and the two higher affinity binders (hRBD2, K_D = 0.07 nM and hRBD10, K_D = 0.17 nM)) is possible under these conditions. At these sub-saturating conditions (antigen concentration 10x lower than its K_D), the fluorescence signal generated by hRBD8 would probably not have been enough for the antibody to be selected in the screen and it would have been difficult to distinguish between the two higher affinity binders. To fine-tune affinity selections, we refer to considerations put forward for affinity screens with yeast surface display[1].

References

1. Boder, E. T. & Wittrup, K. D. Yeast surface display for directed evolution of protein expression, affinity, and stability. *Methods Enzymol.* **328**, 430–444 (2000).
