# Supplementary material for: Rapid discovery of monoclonal antibodies by microfluidics-enabled FACS of single pathogen-specific antibody-secreting cells
Source: Nat Biotechnol. 2024 Aug 14;43(6):960–70. doi: 10.1038/s41587-024-02346-5 (PMC12167710; doi:10.1038/s41587-024-02346-5)
Supplement: Supplementary file 2 — Reporting Summary [file 41587_2024_2346_MOESM2_ESM.pdf]

nature portfolio

Corresponding author(s): Florian Hollfelder

Last updated by author(s): Jul 16, 2024

# Reporting Summary

Nature Portfolio wishes to improve the reproducibility of the work that we publish. This form provides structure for consistency and transparency in reporting. For further information on Nature Portfolio policies, see our Editorial Policies and the Editorial Policy Checklist.

## Statistics

For all statistical analyses, confirm that the following items are present in the figure legend, table legend, main text, or Methods section.

| n/a | Confirmed | |
|---|---|---|
| ☐ | ☒ | The exact sample size (*n*) for each experimental group/condition, given as a discrete number and unit of measurement |
| ☒ | ☐ | A statement on whether measurements were taken from distinct samples or whether the same sample was measured repeatedly |
| ☐ | ☒ | The statistical test(s) used AND whether they are one- or two-sided<br>*Only common tests should be described solely by name; describe more complex techniques in the Methods section.* |
| ☒ | ☐ | A description of all covariates tested |
| ☒ | ☐ | A description of any assumptions or corrections, such as tests of normality and adjustment for multiple comparisons |
| ☐ | ☒ | A full description of the statistical parameters including central tendency (e.g. means) or other basic estimates (e.g. regression coefficient) AND variation (e.g. standard deviation) or associated estimates of uncertainty (e.g. confidence intervals) |
| ☐ | ☒ | For null hypothesis testing, the test statistic (e.g. *F*, *t*, *r*) with confidence intervals, effect sizes, degrees of freedom and *P* value noted<br>*Give P values as exact values whenever suitable.* |
| ☒ | ☐ | For Bayesian analysis, information on the choice of priors and Markov chain Monte Carlo settings |
| ☒ | ☐ | For hierarchical and complex designs, identification of the appropriate level for tests and full reporting of outcomes |
| ☒ | ☐ | Estimates of effect sizes (e.g. Cohen's *d*, Pearson's *r*), indicating how they were calculated |

*Our web collection on statistics for biologists contains articles on many of the points above.*

## Software and code

Policy information about availability of computer code

| | |
|---|---|
| Data collection | Sorts on the Aria III: FACSDiva version 9.0.1 (BD), analysis on AttuneNxt: Attune NxT software version 3.1.2 (Thermo Fisher), analysis on BD LSRFortessa: FACSDiva version 9.0.1 (BD), Sanger sequencing: 3730xl DNA Analyser software (Applied Biosystems), BLI data collection: Octet software (Sartorius), ELISA measurements on Infinite 200 PRO Tecan: i-control software 1.10 (Tecan) and on Varioskan LUX: SkanIt software 6.1.0.5.1 (Thermo Fisher), luminescence measurements: ClarioStar software (BMG Labtech). |
| Data analysis | Flow cytometry data were analysed using FlowJo (version 10.7). Analysis of Sanger sequencing reads was performed using Geneious Prime (version 2019.2.1, Biomatters). Antibody sequences (V gene, J gene, nt identity, CDR3) were analysed with IMGT/V-Quest from the IMGT database (Brochet at al., 2008). All other bioinformatics were performed using Python. Clonotyping was performed using an in-house script (code available in Supplementary Information). Structural clustering was performed using the SPACE algorithm51 (codebase released with the original paper at https://doi.org/10.1371/journal.pcbi.1009675). All BLI data were analysed using the inbuilt Octet data analysis software (version 11.0.0.4). Microscopy data were analysed using Fiji (Schindelin et al., 2012). X-ray diffraction data were processed using autoPROC (version 1.0.2) and Staraniso (version 1.0.2). Global Phasing Ltd). XIA2 (ver. 3.8.dev0) and DIALS (ver. 3.dev.620-g8f4b6839d) were used for crystallographic diffraction data processing, Phenix (version 1.16), CCP4 package (version 7.1), Buster (version 2.10.4) and Coot (version 0.9.8.1) were used for structure determination. PyMOL (version 2.5.0) was used for structure analysis and for figures. Additional data were analysed using GraphPad Prism 6 and Microsoft Excel. Adobe Illustrator (2015) was used to create figures and illustrations. |

For manuscripts utilizing custom algorithms or software that are central to the research but not yet described in published literature, software must be made available to editors and reviewers. We strongly encourage code deposition in a community repository (e.g. GitHub). See the Nature Portfolio guidelines for submitting code & software for further information.

## Data

Policy information about availability of data

All manuscripts must include a data availability statement. This statement should provide the following information, where applicable:

- Accession codes, unique identifiers, or web links for publicly available datasets
- A description of any restrictions on data availability
- For clinical datasets or third party data, please ensure that the statement adheres to our policy

All reference data used in clustering were sourced from publicly available studies via the Coronavirus Antibody Database (CoV-AbDab, http://opig.stats.ox.ac.uk/webapps/covabdab/). Sequences of experimentally verified antigen-specific antibodies were deposited at Genbank (accession numbers OQ208846 - OQ208931). Sequences of anti-SARS-CoV-2 antibodies were also deposited in the CoV-AbDab. Crystallographic data were accessed through RCSB PDB (accession numbers: 6YM0, 3BGF, 6M0J). Crystallographic data were deposited at RCSB PDB under accession number 8BE1. A file of the microfluidic chip design is available on DropBase (https://openwetware.org/wiki/DropBase:agarose_bead_generator). The plasmid for His-Zbasic-TEV-RBD-Avi is available on Addgene (no. 195000). Flow cytometry raw data files and other plasmids generated in this study are available on request. All other data are available in the main text or the Supplementary Information/Source Data.

## Human research participants

Policy information about studies involving human research participants and Sex and Gender in Research.

| | |
|---|---|
| Reporting on sex and gender | Two individuals were sampled: one male one female. Sex was not considered in the study design and would not effect any of the significant results presented in this report. |
| Population characteristics | The research participants were 38 (female) and 48 (male) years old. Both individuals were healthy with no known medical diagnosis. |
| Recruitment | These subjects were recruited as study participants involved in COVID-19 vaccine studies within the host laboratory. They were enrolled based on their clinically healthy status and previous BNT162b2 vaccination. We are not aware of any biases in recruitment. |
| Ethics oversight | Samples were collected with the written informed consent of all study participants under the NIHR National BioResource-Research Tissue Bank (NBR-RTB) ethics (REC:17/EE/0025). |

Note that full information on the approval of the study protocol must also be provided in the manuscript.

# Field-specific reporting

Please select the one below that is the best fit for your research. If you are not sure, read the appropriate sections before making your selection.

☒ Life sciences ☐ Behavioural & social sciences ☐ Ecological, evolutionary & environmental sciences

For a reference copy of the document with all sections, see nature.com/documents/nr-reporting-summary-flat.pdf

# Life sciences study design

All studies must disclose on these points even when the disclosure is negative.

| | |
|---|---|
| Sample size | No sample size calculation was performed. We wanted to highlight that even from a small number of mouse and human samples, we can generate potent antibody reagents. Participants or mice were not selected based on other immunological analyses (e.g. ELISPOT). No conclusions are drawn regarding a larger population. Antibodies for expression were selected based on the criteria outlined in the manuscript, the number of antibodies chosen for expression was limited by the ability of a single researcher to express, purify and test them within a short time frame. |
| Data exclusions | Antibodies were excluded from further testing if the plasmid sequencing did not match the expected sequence or if it was not possible to express the antibody in a single attempt. |
| Replication | Two independent experiments were performed in technical duplicate for all ELISAs for monoclonal antibody testing and neutralization assays. Experiments were performed in technical duplicate for ELISA for IgG quantification in culture supernatant. Confocal imaging was performed for two independent experiments. FACS of antigen-specific ASCs was performed for two independent experiments except for sorting of SARS-CoV-2 S1-specific human ASCs which was performed once. Only cells from one of the two independent experiments were sorted and sequenced. Antibody affinity measurements by BLI were performed once but using at least four different analyte concentrations. All experiments were replicated successfully. |
| Randomization | Non-interventional study. No experimental groups were employed in this work and randomization was therefore neither possible nor relevant. |

Blinding

Non-interventional study. No experimental groups were employed in this work and randomization was therefore neither possible nor relevant.

# Reporting for specific materials, systems and methods

We require information from authors about some types of materials, experimental systems and methods used in many studies. Here, indicate whether each material, system or method listed is relevant to your study. If you are not sure if a list item applies to your research, read the appropriate section before selecting a response.

## Materials & experimental systems

| n/a | Involved in the study |
|---|---|
| ☐ | ☒ Antibodies |
| ☐ | ☒ Eukaryotic cell lines |
| ☒ | ☐ Palaeontology and archaeology |
| ☐ | ☒ Animals and other organisms |
| ☒ | ☐ Clinical data |
| ☒ | ☐ Dual use research of concern |

## Methods

| n/a | Involved in the study |
|---|---|
| ☒ | ☐ ChIP-seq |
| ☐ | ☒ Flow cytometry |
| ☒ | ☐ MRI-based neuroimaging |

## Antibodies

Antibodies used

Antibody capture reagents
Anti-mouse kappa VHH (TP1170, Pleiner et al., 2018), anti-human kappa VHH (HuFab kappa 1, Hermans et al., 2004, patent WO2006/059904A1) and anti-human lambda VHH (HuFab lambda 1, Hermans et al., 2004, patent WO2006/059904A1) were expressed as described in the manuscript.

Antibodies used in flow cytometry and microscopy
Anti-streptavidin antibody (clone 3A20.2, BioLegend, cat# 410501, RRID: AB_2564569)
anti-mouse CD138 PE (clone 281-2, BioLegend, cat#: 142504, RRID: AB_10916119)
anti-mouse CD138 BV785 (clone 281-2, BioLegend, cat# 142534, RRID: AB_2814047)
anti-mouse CD138 Alexa Flour 647 (clone 281-2, BioLegend, cat# 142526, RRID: AB_2566239)
anti-FLAG PE-Cy7 (clone L5, BioLegend, cat#: 637324, RRID: AB_2750063)
anti-mouse IgG1 BV421 (clone RMG1-1, BioLegend, cat#: 406616, RRID: AB_2562234)
anti-mouse IgG2a/2b BV421 (clone R2-40, BD, cat# 744292, RRID: AB_2742122)
anti-mouse IgG2a BV421 (clone RMG2a-62, BioLegend, cat# 407117, RRID: AB_2687343)
anti-mouse IgG3 BV421 (clone R40-82, BD, cat# 565808, RRID: AB_2739364)
anti-mouse IgM BV605 (clone RMM-1, BioLegend, cat# 406523, RRID: AB_2563358)
anti-mouse/human CD45R/B220 BV785 (clone RA3-6B2, BioLegend, cat# 103246, RRID: AB_2563256)
anti-mouse CD138 BV785 (clone 281-2, BioLegend, cat# 142534, RRID: AB_2814047)
anti-mouse IgG1 PerCP-Cy5.5 (clone RMG1-1, BioLegend, cat# 406612, RRID: AB_2562000)
anti-mouse/human CD45R/B220 AF647 (clone RA3-6B2, BioLegend, cat# 103226, RRID: AB_389330)
donkey anti-mouse IgG Alexa Fluor Plus 405 (polyclonal, Thermo Fisher, cat# A48257, RRID: AB_2884884)
anti-human IgM antibody (polyclonal, Jackson ImmunoResearch, cat# 109-006-129, RRID: AB_2337553),
anti-human CD14 VioBlue (clone TÜK4, Miltenyi, cat# 130-113-152, RRID: AB_2725980)
anti-human CD3 VioBlue (clone BW264/56, Miltenyi, cat# 130-113-695, RRID:AB_2726236)
anti-human IgD VioBlue (clone IgD26, Miltenyi, cat# 130-123-258, RRID: AB_2802018)
anti-human CD19 VioBright-FITC (clone LT19, Miltenyi, cat# 130-113-173, RRID: AB_2726000)
anti-human CD27 APC-Vio770 (clone M-T271, Miltenyi, cat# 130-113-627, RRID: AB_2751155)
anti-human CD20 PE-Vio770 (clone LT20, Miltenyi, cat# 130-113-937, RRID: AB_2733214)
anti-human CD38 APC (clone IB6, Miltenyi, cat# 130-113-424, RRID: AB_2733375)
anti-human IgG Fc PE (clone HP6017, BioLegend, cat# 409304 (discontinued), RRID: AB_10895907)
anti-human IgM BV711 (clone MHM-88, BioLegend, cat# 314540, RRID: AB_2687215)
anti-FLAG Alexa Fluor 594 (clone L5, BioLegend, cat# 637314, RRID: AB_2810689)
anti-human IgM BV785 (clone MHM-88, BioLegend, cat# 314544, RRID: AB_2800832)
anti-human CD38 BV421 (clone HB-7, BioLegend, cat# 356618, RRID: AB_2566231)
anti-human CD20 BV510 (clone 2H7, BioLegend, cat# 302340, RRID: AB_2561941)
anti-human IgA PE (clone IS11-8E10, Miltenyi, cat# 130-114-002, RRID: AB_2733860)
anti-human CD38 BV510 (clone HB-7, BioLegend, cat# 356612, RRID: AB_2563875)
anti-human CD20 PE-Cy7 (clone 2H7, BioLegend, cat# 302312, RRID: AB_314260)

Antibodies for ELISA and biolayer interferometry
mouse anti-SARS-CoV-2 Spike RBD (clone 1035753, R&D systems, cat# MAB105808, RRID: AB_2927628)
human anti-SARS-CoV-2 S1/RBD antibody (clone AM001414, BioLegend, cat# 938702, RRID: AB_2876764)
mouse IgG1κ isotype control (clone MG1-45, BioLegend, cat# 401402, RRID: AB_2801451)
human IgG1κ isotype control (clone QA16A12, BioLegend, cat# 403501, RRID: AB_2927629)
goat anti-mouse IgG (H+L) HRP conjugated secondary antibody (polyclonal, Thermo Fisher, cat# G-21040, RRID: AB_2536527)
goat anti-human IgG (H+L) HRP conjugated secondary antibody (polyconal, Abcam, cat# ab7153, RRID: AB_955414)
IgG (Total) Mouse Uncoated ELISA Kit (#88-50400, Invitrogen)
biotinylated anti-mouse IgG1 antibody (clone RMG1-1, BioLegend, cat# 406604, RRID: AB_315063)

human IgG1λ isotype control (anti-GFP, clone AbD00264_hIgG1, BioRad, cat#HCA049, RRID: AB_1102929)
mouse IgG1κ isotype control (clone P3.6.2.8.1, Thermo Fisher, cat# 14-4714-85, RRID: AB_470112)

Antibodies used for crosslinking and stimulation
anti-human IgM antibody (F(ab')$_2$ Fragment, goat polyclonal, Jackson ImmunoResearch, cat# 109-006-129, RRID: AB_2337553),
anti-HA tag antibody (clone 543851, R&D Systems, cat# MAB060, RRID: AB_10719128)

Antibodies used for viral neutralization assays
REGEN-COV (Ronapreve) monoclonal antibody cocktail (casirivimab and imdevimab, Regeneron Pharmaceuticals)

All other used antibodies were generated in this study, sequences can be found in the Supplementary Information.

Validation

Antibody capture reagents (VHHs) were characterised in the publications cited in the manuscript and were tested for binding in BLI experiments. Commercially obtained antibodies were QC tested and validated by the respective suppliers (see below).

Antibodies from Biolegend
All of our products undergo industry-leading rigorous quality control (QC) testing to ensure the highest level of performance and reproducible results. Each lot is compared to an internally established "gold standard" to maintain lot-to-lot consistency. We also conduct wide-scale stability studies to guarantee an accurate shelf-life for our products. Additionally, we test the majority of our products on endogenous cells rather than transfected or immortal cells that may overexpress the analyte.
Validation of flow cytometry reagents: Specificity testing of 1-3 target cell types with either single- or multi-color analysis (including positive and negative cell types). Once specificity is confirmed, each new lot must perform with similar intensity to the in-date reference lot. Brightness (MFI) is evaluated from both positive and negative populations. Each lot product is validated by QC testing with a series of titration dilutions. Each lot of the antibodies is quality control tested by immunofluorescent staining with flow cytometric analysis.

anti-streptavidin antibody (clone 3A20.2, BioLegend, cat# 410501, RRID: AB_2564569), validated for flow cytometry
anti-mouse CD138 PE (clone 281-2, BioLegend, cat#: 142504, RRID: AB_10916119), validated for flow cytometry, verified reactivity: mouse
anti-mouse CD138 BV785 (clone 281-2, BioLegend, cat# 142534, RRID: AB_2814047), validated for flow cytometry, verified reactivity:mouseanti-mouse CD138 Alexa Flour 647 (clone 281-2, BioLegend, cat# 142526, RRID: AB_2566239), validated for flow cytometry, additional reported applications include: immunofluorescent staining (Miettinen H, et al. 1994. J. Cell. Sci. 107:1571, Li Q, et al. 2002. Cell 111:635), verified reactivity: mouse
anti-mouse CD138 BV785 (clone 281-2, BioLegend, cat# 142534, RRID: AB_2814047), validated for flow cytometry, verified reactivity: mouse
anti-FLAG PE-Cy7 (clone L5, BioLegend, cat#: 637324, RRID: AB_2750063), validated for flow cytometry
anti-FLAG Alexa Fluor 594 (clone L5, BioLegend, cat# 637314, RRID: AB_2810689), validated for flow cytometry
anti-mouse IgG1 BV421 (clone RMG1-1, BioLegend, cat#: 406616, RRID: AB_2562234), verified reactivity: mouse, validated for flow cytometry.
anti-mouse IgG1 PerCP-Cy5.5 (clone RMG1-1, BioLegend, cat# 406612, RRID: AB_2562000), validated for flow cytometry, verified reactivity: mouse
biotinylated anti-mouse IgG1 antibody (clone RMG1-1, BioLegend, cat# 406604, RRID: AB_315063), validated for ELISA, verified reactivity: mouse. We used this antibody in a biolayer interferometry competition assay as it is the same clone as we used in flow cytometry stainings.
anti-mouse IgG2a BV421 (clone RMG2a-62, BioLegend, cat# 407117, RRID: AB_2687343), validated for flow cytometry. The RMG2a-62 monoclonal antibody reacts with immunoglobulin G2a and G2c (IgG2a, IgG2c) and in all tested mouse haplotype (Igh-a and b).
anti-mouse IgM BV605 (clone RMM-1, BioLegend, cat# 406523, RRID: AB_2563358), verified reactivity: mouse, validated for flow cytometry
anti-mouse/human CD45R/B220 BV785 (clone RA3-6B2, BioLegend, cat# 103246, RRID: AB_2563256), validated for flow cytometry, verified reactivity: mouse and human
anti-mouse/human CD45R/B220 AF647 (clone RA3-6B2, BioLegend, cat# 103226, RRID: AB_389330), validated for flow cytometry, verified reactivity: mouse and human
anti-human IgG Fc PE (clone HP6017, BioLegend, cat# 409304 (discontinued), RRID: AB_10895907), verified reactivity: human, validated for flow cytometry
anti-human IgM BV711 (clone MHM-88, BioLegend, cat# 314540, RRID: AB_2687215), validated for flow cytometry, verified reactivity: human
anti-IgM BV785 (clone MHM-88, BioLegend, cat# 314544, RRID: AB_2800832), validated for flow cytometry, verified reactivity: human
anti-human CD38 BV421 (clone HB-7, BioLegend, cat# 356618, RRID: AB_2566231), validated for flow cytometry, verified reactivity: human
anti-human CD38 BV510 (clone HB-7, BioLegend, cat# 356612, RRID: AB_2563875), validated for flow cytometry, verified reactivity: human
anti-CD20 BV510 (clone 2H7, BioLegend, cat# 302340, RRID: AB_2561941), validated for flow cytometry, verified reactivity: human
anti-CD20 PE-Cy7 (clone 2H7, BioLegend, cat# 302312, RRID: AB_314260), validated for flow cytometry, verified reactivity: human
human anti-SARS-CoV-2 S1/RBD antibody (clone AM001414, BioLegend, cat# 938702, RRID: AB_2876764), validated for ELISA, verified reactivity: SARS-CoV-2. Each lot is quality control tested by its ability to block the binding between recombinant human ACE2 (Cat. No. 792002) and SARS-CoV-2 S protein S1-Fc chimera (Cat. No. 793004).
mouse IgG1κ isotype control (clone MG1-45, BioLegend, cat# 401402, RRID: AB_2801451). The MG1-45 immunoglobulin is useful as an isotype-matched control for Western blotting, immunoprecipitation, immunohistochemistry, functional assay, immunofluorescence microscopy, immunocytochemistry and immunofluorescent staining (surface or intracellular) for flow cytometric analysis. It was chosen as an isotype control after screening on a variety of resting, activated, live, and fixed mouse, rat and human tissues. Each lot of this antibody is quality control tested by immunofluorescent staining with flow cytometric analysis as negative control
human IgG1κ isotype control (clone QA16A12, BioLegend, cat# 403501, RRID: AB_2927629). QA16A12 is a full length recombinant human IgG1 consisting of both heavy and light chains. It can be used as an isotype control when evaluating fully human, humanized,

or chimeric antibodies. This product is suitable as an isotype-matched control for ELISA, immunoblotting, FACS, IHC, and functional studies. Each lot of this antibody is quality control tested by immunofluorescent staining with flow cytometric analysis as negative control.

Antibodies from BD Biosciences
The specificity is confirmed by using multiple applications that may include a combination of flow cytometry, immunofluorescence, immunohistochemistry or western blot to test a combination of primary cells, cell lines or transfectant models. All flow cytometry reagents are titrated on the relevant positive or negative cells.

anti-mouse IgG2a/2b BV421 (clone R2-40, BD, cat# 744292, RRID: AB_2742122), validated for flow cytometry, verified reactivity: mouse. The R2-40 monoclonal antibody specifically recognizes a common epitope, probably located in the CH1 domain, shared by mouse IgG2a, and IgG2b, of Igh-Ca and Igh-Cb haplotypes. It does not react with other Ig isotypes.
anti-mouse IgG3 BV421 (clone R40-82, BD, cat# 565808, RRID: AB_2739364), validated for flow cytometry, verified reactivity: mouse

Antibodies from Miltenyi Biotec
We follow a certified quality management system in compliance with international standards. Every step during production of an antibody is rigorously monitored and controlled by adequate measures that follow predefined standard operating procedures (SOPs). This includes entry controls of all raw materials, in-process controls, and quality assays of pure antibody batches, as well as final products.
After a product has passed all in-process control steps, it is finally filled into vials. At this stage, it goes through rigorous QC testing to guarantee high quality and consistent product performance from lot to lot. The final quality control is also performed to ensure that every product sent to customers has been tested in its final formulation and filling. This is performed for a given application on suitable tissue samples with internally defined specifications. Some of our antibodies can be used for several applications, although they will have been initially tested and released for only one of these. In such cases, not every application has been run through our final QC testing procedure, but has been instead reported or tested during development. You can easily find this information on every product page within the application section (reported, during development, or QC tested).

anti-human CD14 VioBlue (clone TÜK4, Miltenyi, cat# 130-113-152, RRID: AB_2725980), validated for flow cytometry, verified reactivity: human
anti-human CD3 VioBlue (clone BW264/56, Miltenyi, cat# 130-113-695, RRID: AB_2726236), validated for flow cytometry, verified reactivity: human
anti-human IgD VioBlue (clone IgD26, Miltenyi, cat# 130-123-258, RRID: AB_2802018), validated for flow cytometry, verified reactivity: human
anti-human CD19 VioBright-FITC (clone LT19, Miltenyi, cat# 130-113-173, RRID: AB_2726000), validated for flow cytometry, verified reactivity: human
anti-human CD27 APC-Vio770 (clone M-T271, Miltenyi, cat# 130-113-627, RRID: AB_2751155), validated for flow cytometry, verified reactivity: human
anti-human CD20 PE-Vio770 (clone LT20, Miltenyi, cat# 130-113-937, RRID: AB_2733214), validated for flow cytometry, verified reactivity: human
anti-human CD38 APC (clone IB6, Miltenyi, cat# 130-113-424, RRID: AB_2733375), validated for flow cytometry, verified reactivity: human
anti-IgA PE (clone IS11-8E10, Miltenyi, cat# 130-114-002, RRID: AB_2733860), validated for flow cytometry, verified reactivity: human. The Anti-IgA antibody clone IS11-8E10 detects both subclasses of human IgA. IgA is present either as monomer or in a secreted form as a multimer of 2–4 molecules, connected by the J-chain and the so-called secretory component.

Antibodies from Thermo Fisher/Invitrogen
Each Invitrogen antibody that is indicated for immunocytochemistry applications has undergone functional application testing.
Each Invitrogen ELISA kit meets rigorous specifications and is manufactured with stringent quality controls to help ensure excellent quality and reproducibility. Invitrogen ELISA kits are tested for the following factors—intra-assay precision, inter-assay precision, linearity of dilution, ELISA parallelism, recovery, sensitivity, and specificity. Data acquired from these ELISA validation tests are typically available in each kit's manual—found either in the kit packaging or on the product webpage.
donkey anti-mouse IgG Alexa Fluor Plus 405 (polyclonal, Thermo Fisher, cat# A48257, RRID: AB_2884884), validated for immunocytochemistry/immunofluorescence, verified reactivity: mouse. Cross adsorption against serum proteins from bovine, goat, chicken, guinea pig, hamster, horse, sheep, rabbit, rat, and human was performed. This antibody binds to heavy chains on mouse IgG and light chains on all mouse immunoglobulins. This antibody does not bind non-immunoglobulin mouse serum proteins or IgG from bovine, chicken, goat, guinea pig, hamster, horse, human, rabbit, rat or sheep.
goat anti-mouse IgG (H+L) HRP conjugated secondary antibody (polyclonal, Thermo Fisher, cat# G-21040, RRID: AB_2536527), validated for ELISA, verified reactivity: mouse. Cross adsorption against human IgG and human serum was performed.
mouse IgG1κ isotype control (clone P3.6.2.8.1, Thermo Fisher, cat# 14-4714-85, RRID: AB_470112). Mouse IgG1 K Isotype Control has been tested by flow cytometric analysis of normal human peripheral blood cells and mouse spleen cells. It has been reported for use in surface and intracellular flow cytometric analysis, immunohistochemistry, immunocytochemistry, immunoprecipitation and immunoblotting (WB).
The IgG (Total) Mouse Uncoated ELISA Kit (#88-50400, Invitrogen) was used according to the manufacturer's instructions.

Antibodies from other vendors
anti-HA tag antibody (clone 543851, R&D Systems, cat# MAB060, RRID: AB_10719128). Specifically detects N-terminal HA-tagged proteins with a free N-terminus. The antibody was used for crosslinking of HA-tagged CD40 ligand in several publications (Price et al., eLife (2017), doi: 10.7554/eLife.22509; Grossman et al., mSphere (2017), doi: 10.1128/msphere.00305-17; Seifert et al., PNAS (2015), doi: 10.1073/pnas.1416276112; Nikitin et al., PLOS One (2014), doi: 10.1371/journal.pone.0087299).
mouse anti-SARS-CoV-2 Spike RBD (clone 1035753, R&D systems, cat# MAB105808, RRID: AB_2927628), validated in ELISA. In a functional flow cytometry assay, this antibody binds and blocks SARS-CoV-2 Spike RBD interaction with human ACE-2 transfectants. Detects SARS-CoV-2 Spike RBD and SARS-CoV-2 Spike S1 in sandwich ELISAs when used in the indicated ELISA antibody pairings. Detects SARS-CoV-2 B.1.1.529 S RBD (Omicron Variant) in direct ELISAs.
anti-human IgM antibody (F(ab')$_2$ Fragment, goat polyclonal, Jackson ImmunoResearch, cat# 109-006-129, RRID: AB_2337553). Based on immunoelectrophoresis and/or ELISA, the antibody reacts with the Fc5μ portion of the human IgM heavy chain but not with human IgG, IgA, or the light chains of human immunoglobulins. No antibody was detected against non-immunoglobulin serum

proteins. The antibody has been tested by ELISA and/or solid-phase adsorbed to ensure minimal cross-reaction with bovine serum proteins. The antibody has been used previously for BCR crosslinking (e.g. Espinoza et al., JCI Insight (2023), doi: 10.1172/jci.insight.155199.)
goat anti-human IgG (H+L) HRP conjugated secondary antibody (polyconal, Abcam, cat# ab7153, RRID: AB_955414), validated in ELISA. No reaction was observed against Bovine, Chicken, Goat, Guinea Pig, Hamster, Horse, Mouse, Rabbit, Rat and Sheep Serum Proteins.
human IgG1λ isotype control (anti-GFP, clone AbD00264_hIgG1, BioRad, cat# HCA049, RRID: AB_1102929), validated in ELISA. This product is a recombinant antibody with specificity for green fluorescent protein (GFP). It has no known reactivity with mammalian proteins or other antigens. It is therefore recommended as a control reagent for assays where this immunoglobulin is measured, or when using other human IgG1 antibodies of the same isotype or allotype.
The clinically approved REGEN-COV (Ronapreve, Regeneron Pharmaceuticals) monoclonal antibody cocktail (casirivimab and imdevimab) was used as a positive control in live virus neutralisation assays against wildtype SARS-CoV-2

## Eukaryotic cell lines

Policy information about cell lines and Sex and Gender in Research

| | |
|---|---|
| Cell line source(s) | FreeStyle 293-F Cells (Thermo Fisher Scientific, Cat#R790-07), HEK293T-ACE2-30F-PLP2 cells (clone B7, available from the National Institute for Biological Standards and Control (NIBSC), catalogue number 101062), A549-ACE2-TMPRSS2-30F-PLP2 cells (clone E8). These cell lines were generated in the Matheson lab as previously described (Pereyra Gerber et al., 2022 and Meng et al., 2022). |
| Authentication | HEK293T-ACE2-30F-PLP2 cells were previously authenticated by STR profiling. A549-ACE2-TMPRSS2-30F-PLP2 and FreeStyle 293-F cells were not authenticated. |
| Mycoplasma contamination | Cells for neutralisation assays (HEK293T-ACE2-30F-PLP2, A549-ACE2-TMPRSS2-30F-PLP2) were regularly screened and confirmed to be mycoplasma negative (Lonza MycoAlert and IDEXX BioAnalytics).<br>FreeStyle 293-F Cells used for antibody expressions were not screened for mycoplasma contamination. |
| Commonly misidentified lines<br>(See ICLAC register) | None. |

## Animals and other research organisms

Policy information about studies involving animals; ARRIVE guidelines recommended for reporting animal research, and Sex and Gender in Research

| | |
|---|---|
| Laboratory animals | Mus musculus C57BL/6, 8 - 14 weeks and 33 - 38 weeks. |
| Wild animals | No wild animals were used in this study. |
| Reporting on sex | Sex was not considered in the study design and would not effect any of the significant results presented in this report. |
| Field-collected samples | No field-collected samples were used in this study. |
| Ethics oversight | Animal experiments were licensed by the UK Home Office according to the Animals Scientific Procedures Act 1986 (License PP6047951) and approved by the local ethics committee from the University of Cambridge. |

Note that full information on the approval of the study protocol must also be provided in the manuscript.

## Flow Cytometry

### Plots

Confirm that:

☒ The axis labels state the marker and fluorochrome used (e.g. CD4-FITC).

☒ The axis scales are clearly visible. Include numbers along axes only for bottom left plot of group (a 'group' is an analysis of identical markers).

☒ All plots are contour plots with outliers or pseudocolor plots.

☒ A numerical value for number of cells or percentage (with statistics) is provided.

### Methodology

| | |
|---|---|
| Sample preparation | Mouse splenocytes and bone marrow<br>A single cell suspension of splenocytes was prepared by mashing the spleens through a 100 µm cell strainer. After washing, cells were then filtered again through a 70 µm strainer.<br>For bone marrow extraction, femur and tibias were removed from both legs, the ends of the bones were cut open with scissors and the bone marrow was flushed out using a 23-gauge needle and a syringe. A single cell suspension was prepared by filtering through a 100 µm cell strainer.<br>Red blood cells of both spleen and bone marrow were lysed, cells were washed once and then used for ASC enrichment (anti- |

CD138 microbeads, Miltenyi, according to the manufacturer's instructions). Cells were then subjected to microfluidic encapsulation followed by FACS as described in the manuscript.

Human PBMCs
B cells were negatively selected from normal cryopreserved human PBMCs (10 million cells/vial, Lonza, 4W-270) with the human pan-B cell isolation kit (Miltenyi) according to the manufacturer's instructions. Enriched B cells were then stimulated with CD40L, IL-21 and anti-IgM for 96 h. After stimulation, CD38+ cells were positively selected using the CD38 MicroBead kit (Miltenyi) according to the manufacturer's instructions. Cells were then subjected to microfluidic encapsulation followed by FACS as described in the manuscript.
Blood was collected from study participants 7 – 9 days post the second BNT162b2 vaccine dose and PBMCs were isolated by density gradient centrifugation. B cells were negatively enriched from freshly isolated PBMCs or thawed cryopreserved PBMCs with the human pan-B cell isolation kit (Miltenyi) according to the manufacturer's instructions. Cells were then subjected to microfluidic encapsulation followed by FACS as described in the manuscript.

| | |
|---|---|
| Instrument | For analysis experiments, a 4-laser AttuneNxT instrument (Thermo Fisher) and a 4-laser LSRFortessa (BD) were used.<br>A 4-laser FACS Aria III (BD) with a 100 µm nozzle was used for sorting experiments. |
| Software | For data collection during sorts on the Aria III and on the LSRFortessa, FACSDiva version 9.0.1 (BD) was used.<br>For data collection on the AttuneNxt, the Attune NxT software version 3.1.2 (Thermo Fisher) was used.<br>Data were analysed using FlowJo (version 10.7, BD Biosciences). |
| Cell population abundance | Cells were sorted into single wells of 96 well plates using a strict single cell sort mask (yield mask: 0, purity mask: 32, phase mask: 8 or yield mask: 0, purity mask: 32, phase mask: 16). We therefore assume that only single cells were sorted. |
| Gating strategy | For mouse anti-OVA sorts, live plasma cells inside hydrogel beads were gated on SSC-A vs. FSC-A (on log-scale), singlets (FSC-H vs FSC-A on log scale) and then as live/FLAG+/CD138+/ B220- (SNAP-VHH contains a FLAG tag). IgG-secreting cells were then gated as IgG+ but IgM-. For secretion controls, the FLAG gate was left out as no FLAG-SNAP-VHH was added. For mouse anti-RBD sorts, live plasma cells inside hydrogel beads were gated on SSC-A vs. FSC-A (on log-scale) and then as live/CD138+. IgG-secreting cells were then gated as IgG+ but IgM-. The antigen signals (OVA or RBD) of hydrogel beads that were IgG-/IgM- were used as guidance for setting the antigen positive sort gate. For the viability time course, live plasma cells were gated as on SSC-A vs. FSC-A (on linear scale), singlets (FSC-H vs FSC-A on linear scale) and then as CD138+ and live (DAPI-). For the IgG secretion time course, cells were gated on SSC-A vs. FSC-A (on linear scale), singlets (FSC-H vs FSC-A on linear scale) and then as live/CD138+ or /CD138- (empty beads and encapsulated non-plasma cells) followed by gating as IgG+. The IgG-positive population after 1 h was used to set the IgG gate.<br>For human sorts, live plasmablasts inside hydrogel beads were gated on SSC-A vs. FSC-A (on log-scale) and then as live/CD20-/CD38+. IgG/IgA secreting cells were then gated as IgG/IgA+ but IgM-. The antigen signal of hydrogel beads that were IgG/A-/IgM- were used as guidance for setting the antigen positive sort gate.<br><br>Gating strategies are shown in detail in the extended data figures 4 - 9 and Supplementary Figures 2 - 3. |

☒ Tick this box to confirm that a figure exemplifying the gating strategy is provided in the Supplementary Information.
